# Benchmark of long non-coding RNA quantification for RNA sequencing of cancer samples

**DOI:** 10.1093/gigascience/giz145

**Published:** 2019-12-06

**Authors:** Hong Zheng, Kevin Brennan, Mikel Hernaez, Olivier Gevaert

**Affiliations:** 1 Stanford Center for Biomedical Informatics Research, Department of Medicine, Stanford University, 1265 Welch Road, Stanford, 94305, CA, USA; 2 Carl R. Woese Institute for Genomic Biology, University of Illinois at Urbana-Champaign, 1206 W. Gregory Dr, Urbana, 61805, IL, USA; 3 Department of Biomedical Data Science, Stanford University, 1265 Welch Road, Stanford, 94305, CA, USA

**Keywords:** long non-coding RNA, RNA sequencing, pseudoalignment

## Abstract

**Background:**

Long non-coding RNAs (lncRNAs) are emerging as important regulators of various biological processes. While many studies have exploited public resources such as RNA sequencing (RNA-Seq) data in The Cancer Genome Atlas to study lncRNAs in cancer, it is crucial to choose the optimal method for accurate expression quantification.

**Results:**

In this study, we compared the performance of pseudoalignment methods Kallisto and Salmon, alignment-based transcript quantification method RSEM, and alignment-based gene quantification methods HTSeq and featureCounts, in combination with read aligners STAR, Subread, and HISAT2, in lncRNA quantification, by applying them to both un-stranded and stranded RNA-Seq datasets. Full transcriptome annotation, including protein-coding and non-coding RNAs, greatly improves the specificity of lncRNA expression quantification. Pseudoalignment methods and RSEM outperform HTSeq and featureCounts for lncRNA quantification at both sample- and gene-level comparison, regardless of RNA-Seq protocol type, choice of aligners, and transcriptome annotation. Pseudoalignment methods and RSEM detect more lncRNAs and correlate highly with simulated ground truth. On the contrary, HTSeq and featureCounts often underestimate lncRNA expression. Antisense lncRNAs are poorly quantified by alignment-based gene quantification methods, which can be improved using stranded protocols and pseudoalignment methods.

**Conclusions:**

Considering the consistency with ground truth and computational resources, pseudoalignment methods Kallisto or Salmon in combination with full transcriptome annotation is our recommended strategy for RNA-Seq analysis for lncRNAs.

## Background

Long non-coding RNAs (lncRNAs) are a diverse class of RNA molecules that are >200 nucleotides in length and do not encode proteins [1]. While functional classification is lacking for most lncRNAs, on the basis of their genomic proximity to protein-coding genes and the direction of transcription, lncRNAs are often classified into antisense, intronic, bidirectional, intergenic, or overlapping RNAs [1]. GENCODE, the database that provides annotations for human genes and transcripts, defines >14,000 human lncRNA genes (release 27, https://www.gencodegenes.org). Other lncRNA databases including NONCODE [2] and MiTranscriptome [3] both collect >60,000 lncRNAs. Compared with protein-coding genes, lncRNAs are shorter, lower-expressed, less evolutionarily conserved, and expressed in a more tissue-specific manner [4]. lncRNAs have recently emerged as an essential class of regulatory elements for many biological processes including imprinting, cell differentiation, and development [5]. They are often disrupted in human diseases including cancer [6]. They may interact with DNA, RNA, and proteins, and exert regulatory roles through a variety of mechanisms. Based on their molecular functions, lncRNA may act as (i) signals, which are indicators of transcriptional activity; (ii) decoys, which bind to and titrate away protein targets such as transcription factors; (iii) guides, which direct regulatory complexes or transcription factors to specific targets and regulate gene expression in *cis* or*trans*; and (iv) scaffolds, which serve as central platforms where relevant molecular components in cells are assembled [7].

lncRNAs have been shown to be important in the pathogenesis of human diseases, especially in cancer, and many cancer-relevant lncRNAs have been identified [8,9]. For example, Hox transcript antisense RNA (HOTAIR), one of the most well-characterized lncRNAs, promotes breast cancer metastasis through recruitment of Polycomb chromatin remodeling complex to silence the HOXD gene cluster [10]. In addition, HOTAIR is overexpressed in breast, liver, lung, and pancreatic cancers [11]. CDKN2B-AS1, an antisense lncRNA encoded by the CDKN2B locus, epigenetically silences nearby tumor suppresser genes and promotes oncogenesis [12]. Telomerase RNA component (TERC), the critical RNA component of telomerase polymerase, serves as a template for the enzyme telomerase reverse transcriptase (TERT) to elongate telomeres. Variants and copy number changes at the TERC locus have been associated with cancer risk and progression [8]. The lncRNA LINC01106 is shown to be differentially expressed in multiple cancer types including lung adenocarcinoma and nasopharyngeal carcinoma [13, 14]. Another lncRNA, LINC01123, is among the 5 most significantly up-regulated lncRNAs in intrahepatic cholangiocarcinoma [15].

The discovery of oncogenic and tumor suppressor lncRNAs has led to an increased interest in the investigation of lncRNAs as potential cancer drug targets and biomarkers. Hence, it is critical to accurately determine lncRNA expression in cancer research. RNA sequencing (RNA-Seq) has been widely used for massive-parallel gene expression quantification. There have been many studies that explore lncRNA expression profile in cancer using publicly available RNA-Seq datasets such as those generated by The Cancer Genome Atlas (TCGA), which provide a rich source of lncRNA expression data in large cancer patient populations [16,17]. Among those studies, the analysis of the lncRNA expression profile of breast cancer samples in TCGA revealed different subtypes of breast cancer and subtype-specific overexpression of HOTAIR [16]. The analysis of 13 cancer types in TCGA revealed highly cancer site–specific lncRNA expression and dysregulation [17].

There are 2 types of RNA-Seq protocols, depending on whether strand specificity information of transcripts is retained in the library preparation step [18]. The standard protocol loses the information regarding which strand the original mRNA template is coming from, which makes it difficult to accurately determine gene expression from overlapping genes. The strand-specific RNA-Seq protocol, such as the deoxyuridine triphosphate (dUTP) method, retains strand origin of transcripts by degrading the second strand in the complementary DNA synthesis step. It has been shown to be more reliable in gene expression quantification and is recommended over the standard protocol [19]. However, the majority of TCGA samples were prepared with non-standard RNA-Seq protocol.

Multiple tools for processing RNA-Seq data have been developed in recent years. While some studies have benchmarked RNA-Seq analysis workflows [20,21], their focus has been primarily on protein-coding genes. There is no accepted gold standard pipeline yet that shows which method performs best to quantify expression of lncRNAs. As the interest in studying lncRNAs in cancer grows, it is necessary to determine which algorithms perform best in lncRNA expression quantification because it is important to understand the differences and limitations of each of them and to follow the best practices of RNA-Seq analysis.

Because of the lower expression and different properties of lncRNAs with respect to protein-coding genes, we hypothesized that the processing and analysis of RNA-Seq data for lncRNA expression may be subjected to different technical biases and challenges, and that special considerations may be necessary to optimize the pipeline specifically for lncRNAs.

To investigate the performance of different methods on the quantification of lncRNAs as well as the effect of different RNA-Seq library preparation protocols, we applied 5 popular quantification methods, Kallisto [22], Salmon [23], RSEM [24], HTSeq [25], and featureCounts [26], on RNA-Seq samples prepared using a standard protocol (i.e., un-stranded) and a strand-specific protocol. Kallisto and Salmon are so-called pseudoalignment methods because they do not align sequencing reads to the reference genome; instead, they use an expectation maximization algorithm to iteratively assign reads to a set of compatible transcripts to obtain the estimated abundances for all transcripts. The alignment-free feature makes pseudoalignment methods much faster than alignment-based methods such as RSEM, HTSeq, and featureCounts because the latter require mapping of the sequencing reads to the genome or transcriptome, which takes substantial time and computational resources. Among the alignment-based methods, RSEM aligns reads to the transcriptome using bowtie as the default aligner and obtains transcript-level expression, while HTSeq and featureCounts use genome-aligned reads to obtain gene-level expression directly. We refer to RSEM as an “alignment-based transcript quantification method" and HTSeq and featureCounts as “alignment-based gene quantification methods." We used 3 aligners, STAR [27], Subread [28], and HISAT2 [29], to map the reads to the genome, before applying HTSeq and featureCounts to count the reads mapped to individual genes.

## Data Description

Both un-stranded and reverse-stranded RNA-Seq data from TCGA samples were downloaded from the ISB Cancer Genomics Cloud. The other reverse-stranded dataset was downloaded from NCBI SRA under the accession PRJEB11797. Read quality control was performed with Trim galore [30], with the setting "-q 20 -stringency 3 -gzip -length 20 -paired." Afterwards the reads were mapped to the human transcriptome (both GENCODE and GENCODE combined with NONCODE) by STAR and were further processed by RSEM [24] (version 1.3.0) to obtain gene and transcript expression. Stand-specific option was set as "–forward-prob 0.5" for un-stranded samples and "–forward-prob 0" for reverse-stranded samples. RSEM and Polyester [31] were then used to generate 2 sets of simulated RNA-Seq reads. In RSEM simulation, RNA-Seq reads were generated with the command "rsem-simulate-reads," which takes as input abundance estimates, sequencing model parameters, and reference transcripts. The abundance estimates and sequencing model are obtained by running RSEM on the real datasets mentioned above. The total number of simulated reads for each sample is 60 million. The simulated reads were 50 bp (simulated from TCGA samples) or 100 bp (simulated from PRJEB11797 data) paired-end reads. The fragment length distribution is 178 ± 60 (mean ± sd) bp for TCGA samples and 155 ± 51 bp for the other dataset. In Polyester estimation, RNA-Seq reads were generated with the command "simulate_experiment_countmat," which takes as input the count matrix of transcripts obtained from the real datasets. Both un-stranded and strand-specific RNA-Seq reads were generated in RSEM and Polyester simulation. The 2 sets of simulated samples with pre-defined gene expression levels serve as the "ground truth" for the evaluation of other pipelines.

## Analyses

### Full transcriptome annotation improves the specificity of RNA quantification

We used RSEM [24] to simulate RNA-Seq reads based on 3 RNA-Seq datasets: (i) 100 un-stranded samples from 10 cancer types in TCGA, (ii) 40 reverse-stranded samples in TCGA, and (iii) 62 reverse-stranded samples from a study of Barrett esophagus and esophageal adenocarcinoma (PRJEB11797) [32]. To evaluate the effect that different transcriptome annotations has on the quantification of gene expression, we built 3 transcriptome annotation sets: (i) full annotation with all 58,288 genes in GENCODE release 27, (ii) partial annotation containing only the 19,836 protein-coding genes, and (iii) partial annotation with only the 14,168 lncRNAs (Additional File 1).

**Figure 1:**
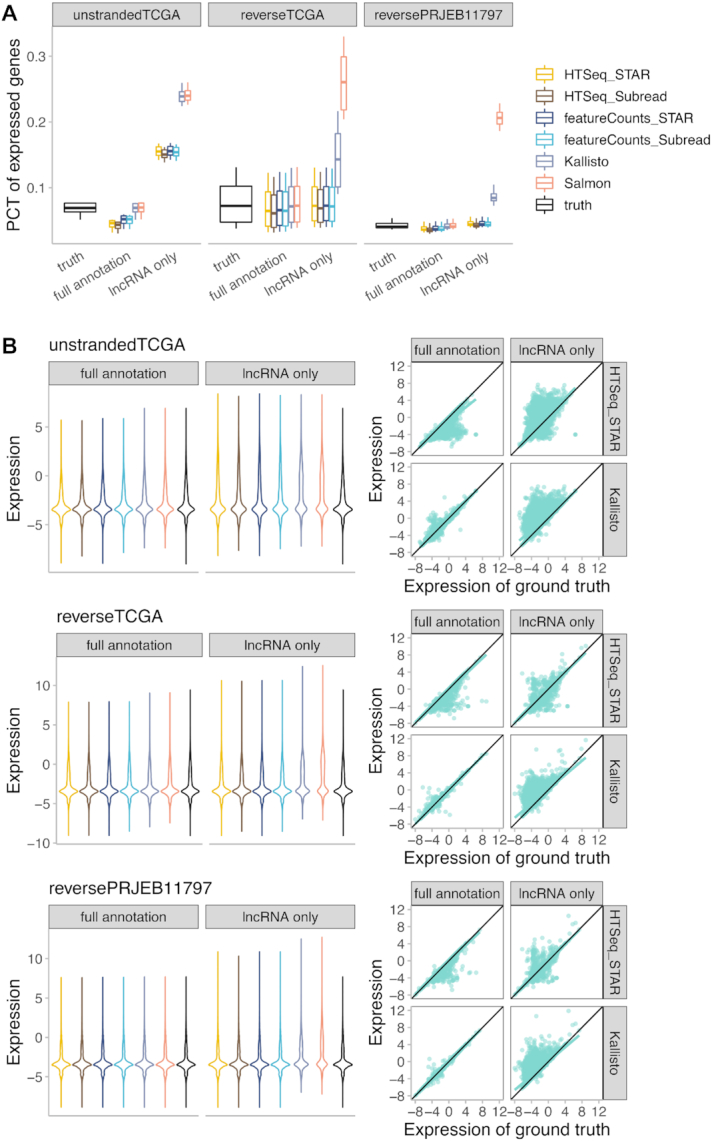
The effect of incomplete transcriptome annotation on the expression quantification of lncRNAs. (A) Box plot of the percentage (PCT) of expressed lncRNAs (fragments per kilobase million [FPKM] ≥ 1) detected with each tool, using full annotation or lncRNA-only annotation. The ground truth expression used for data simulation was also plotted for comparison. Each point in the box plot represents 1 sample. For each library type, 10 samples were included in the analysis. (B) The expression profile of lncRNAs in 1 representative sample from each of the datasets was shown with violin plot (left) and scatter plot (right), which demonstrates the overestimation effect using lncRNA-only annotation compared with full annotation, in all 3 samples for both pseudoalignment and alignment-based methods. In the box plots, the top and bottom of the rectangle represent the third and the first quartiles. The band inside the rectangle is the second quartile (the median). The whiskers above and below the box show the upper and lower fences, which are 1.5 times interquartile range above the third quartile, or 1.5 times interquartile range below the first quartile, respectively.

Using the lncRNA-only annotation overestimates lncRNA expression compared to full annotation (Fig. 1, Additional File 2). The overestimation effect using an incomplete transcriptome annotation set can be observed for all the methods when using either un-stranded or reverse-stranded RNA-Seq libraries, although the effect is less drastic for alignment-based methods when using reverse-stranded libraries. The effect of incomplete transcriptome annotation is less obvious for protein-coding genes, but there is still a slight increase of the percentage of expressed genes when using only protein-coding annotation, compared to full annotation (Additional Files 2 and 3). Thus, using a full annotation improves the specificity of RNA quantification; therefore, it was used in the following analysis.

### Pseudoalignment methods and RSEM outperform HTSeq and featureCounts for lncRNA expression quantification

**Figure 2:**
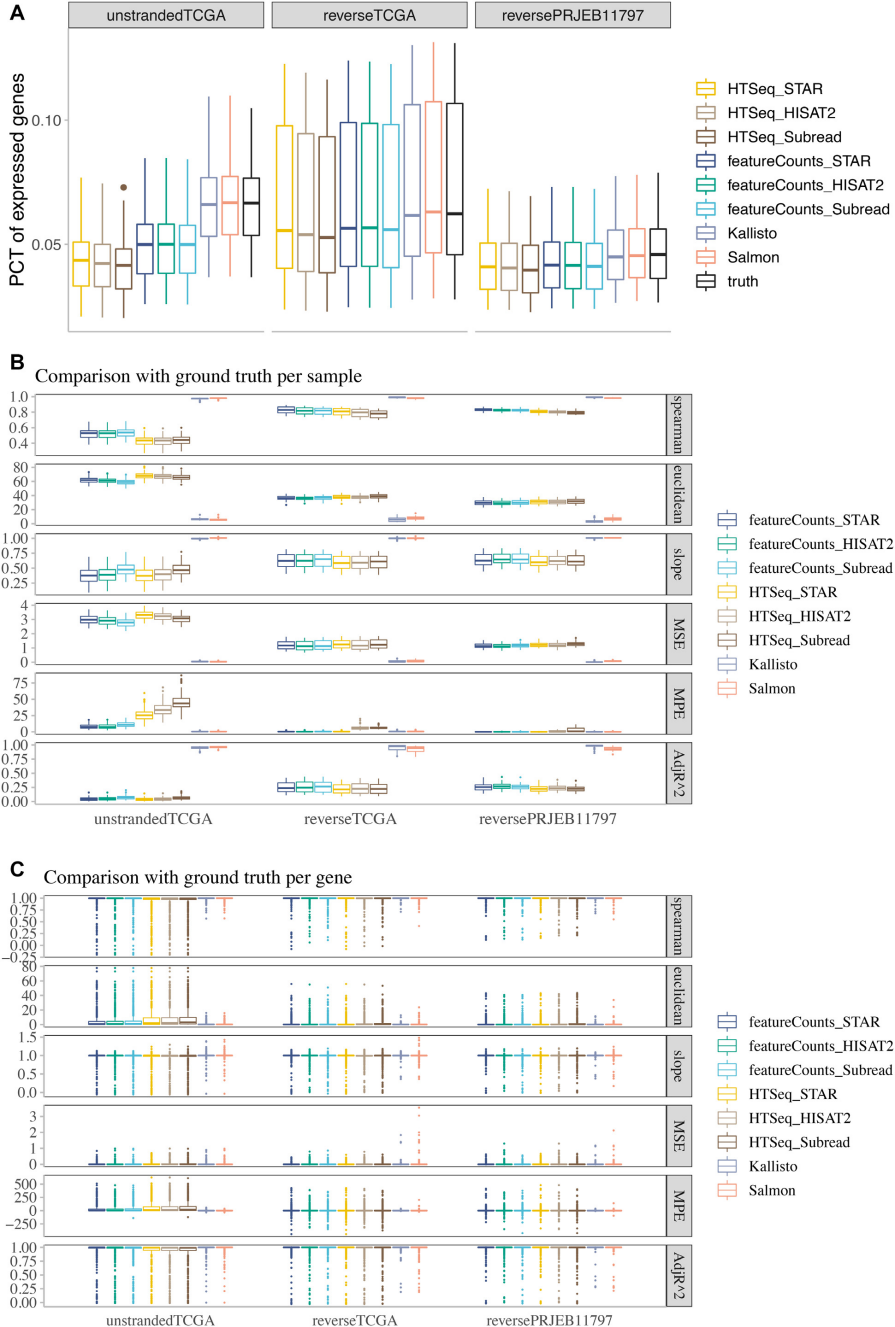
Pseudoalignment methods outperformed alignment-based methods in RSEM-simulated datasets. (A) Box plot of the percentage (PCT) of expressed lncRNAs detected with each tool. Each point in the box plot represents 1 sample. (B) Sample-level and (C) gene-level comparison of each tool with the ground truth. The calculation of Spearman’s correlation and Euclidean distance, and linear regression was performed using log-transformed FPKM values reported by each tool compared with the ground truth. In sample-level comparison, only expressed lncRNAs in each sample were included in the analysis. Each point in the box plot represents 1 sample. In gene-level comparison, lncRNAs with median FPKM > 1 in the corresponding dataset were included in the analysis. Each point in the box plot represents 1 gene. Spearman, Spearman’s rank-order correlation; MSE, mean squared error; MPE, median percent error; AdjR^2, adjusted *R*^2^. In the box plots, the top and bottom of the rectangle represent the third and the first quartiles. The band inside the rectangle is the second quartile (the median). The whiskers above and below the box show the upper and lower fences, which are 1.5 times interquartile range above the third quartile, or 1.5 times interquartile range below the first quartile, respectively.

Pseudoalignment methods detect expression of more genes than alignment-based methods (Fig. 2A, Additional File 4). The average percentage of expressed lncRNAs (fragments per kilobase million [FPKM] ≥ 1 in ground truth) in the simulated ground truth ranges between 4.7% and 7.4% for the 3 RNA-Seq datasets, which is very close to the output of Kallisto and Salmon. The alignment-based methods detect fewer lncRNAs compared to the ground truth, especially for un-stranded samples.

The performance of each method was further evaluated at both sample level (Fig. 2B) and gene level (Fig. 2C). For sample-level evaluation, only expressed lncRNAs (FPKM > 1 in the ground truth) were kept in each sample. The concordance of each method with the ground truth was measured by means of Spearman’s correlation, Euclidean distance, median percent error, and linear regression. Gene expression from Kallisto and Salmon yields the highest Spearman’s correlation, the lowest Euclidean distance, and the lowest median percent error with respect to the ground truth. The 2 pseudoalignment methods also have the highest level of fitness to the ground truth, in terms of the lowest mean squared error, the highest adjusted *R*^2^ value, and a slope value of close to 1 (Fig. 2B, Additional File 5). A similar trend can also be observed for protein-coding genes in GENCODE (Additional File 6A). For gene-level evaluation, a comparison was performed using, for each corresponding dataset, only those lncRNAs with median FPKM > 1 in the ground truth because genes with low read counts are likely to be noise and unlikely to yield reliable results. The number of lncRNAs examined ranges between 464 and 729 for the 3 RNA-Seq datasets. Kallisto and Salmon perform better than alignment-based methods in terms of higher Spearman’s correlation, lower Euclidean distance and median percent error with respect to the ground truth, linear regression slope closer to 1, and higher adjusted *R*^2^ value (Fig. 2C, Additional File 7). The fraction of genes for which the estimates are significantly different (percent error > 5%) from the ground truth is significantly larger in HTSeq and featureCounts than pseudoalignment methods. A similar trend can also be observed for protein-coding genes in GENCODE (Additional File 8A).

**Figure 3:**
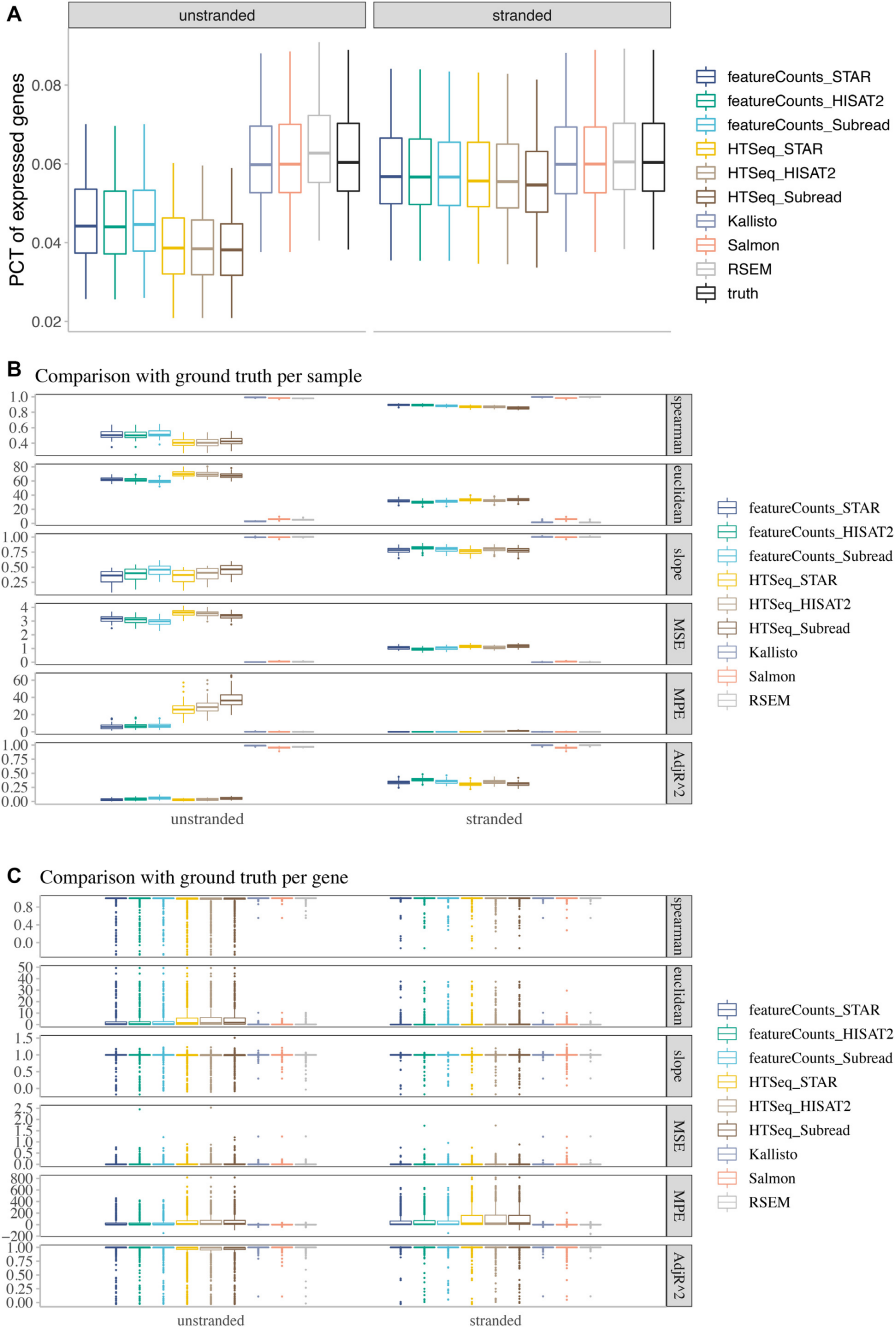
Pseudoalignment methods outperformed alignment-based methods in Polyester-simulated datasets. (A) Box plot of the percentage of expressed lncRNAs detected with each tool. Each point in the box plot represents 1 sample. (B) Sample-level and (C) gene-level comparison of each tool with the ground truth. The calculation of Spearman’s correlation and Euclidean distance, and linear regression was performed using log-transformed FPKM values reported by each tool compared with the ground truth. In sample-level comparison, only expressed lncRNAs in each sample were included in the analysis. Each point in the box plot represents 1 sample. In gene-level comparison, lncRNAs with median FPKM > 1 in the corresponding dataset were included in the analysis. Each point in the box plot represents 1 gene. Spearman, Spearman’s rank-order correlation; PCT, percentage; MSE, mean squared error; MPE, median percent error; AdjR^2, adjusted *R*^2^. In the box plots, the top and bottom of the rectangle represent the third and the first quartiles. The band inside the rectangle is the second quartile (the median). The whiskers above and below the box show the upper and lower fences, which are 1.5 times interquartile range above the third quartile, or 1.5 times interquartile range below the first quartile, respectively.

Because RSEM cannot be assessed in an unbiased manner using RSEM-simulated datasets, we later used the Polyester-simulated datasets to include RSEM in the benchmark. We simulated 40 samples for both un-stranded and strand-specific protocols, and compared RSEM, pseudoalignemnt methods, and alignment-based gene quantification methods with ground truth. The performance of RSEM was similar to pseudoalignment methods and outperformed HTSeq and featureCounts, in terms of the percentage of expressed lncRNAs detected (Fig. 3A) and concordance with ground truth in both sample-level (Fig. 3B) and gene-level (Fig. 3C) comparison.

**Figure 4:**
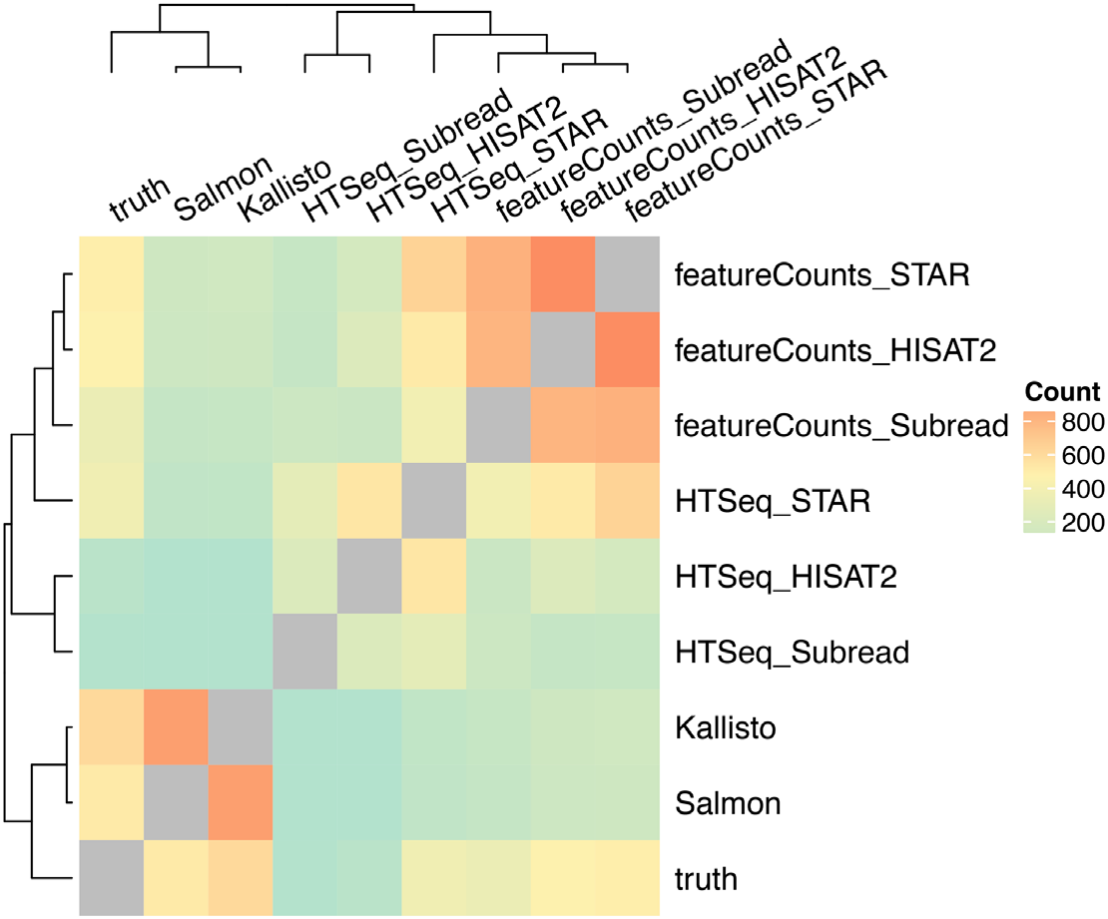
Similarity matrix of different methods. Each grid in the matrix is the number of times that 2 methods were clustered in the same group, which is counted from hierarchical clustering of 1,075 expressed lncRNAs in any of the 3 datasets. The group number for cutting the hierarchical clustering dendrogram was set as 4. Euclidean distance and average linkage were used for single-gene level clustering.

For each of the expressed lncRNAs in any of the 3 datasets, hierarchical clustering was performed to evaluate the similarity of each method’s measurement to the ground truth and between each other (Fig. 4). Kallisto and Salmon often clustered together with the ground truth. In addition, the 3 featureCounts pipelines (STAR+featureCounts, HISAT2+featureCounts, Subread+featureCounts) form another cluster, while pipelines using HTSeq loosely cluster together.

Next, we expanded our analysis and also included lncRNAs from NONCODE, a database collecting 172,216 transcripts from 96,308 lncRNA genes (version 5) [2]. We simulated RNA sequencing reads based on both GENCODE and NONCODE gene annotations and replicated the analysis for lncRNAs in NONCODE. Similar to the results from GENCODE annotation, the 2 pseudoalignment methods outperform alignment-based methods in both sample-level (Additional File 6B) and gene-level comparison (Additional File 8B).

### Characteristics of expressed and discordant lncRNAs

**Figure 5:**
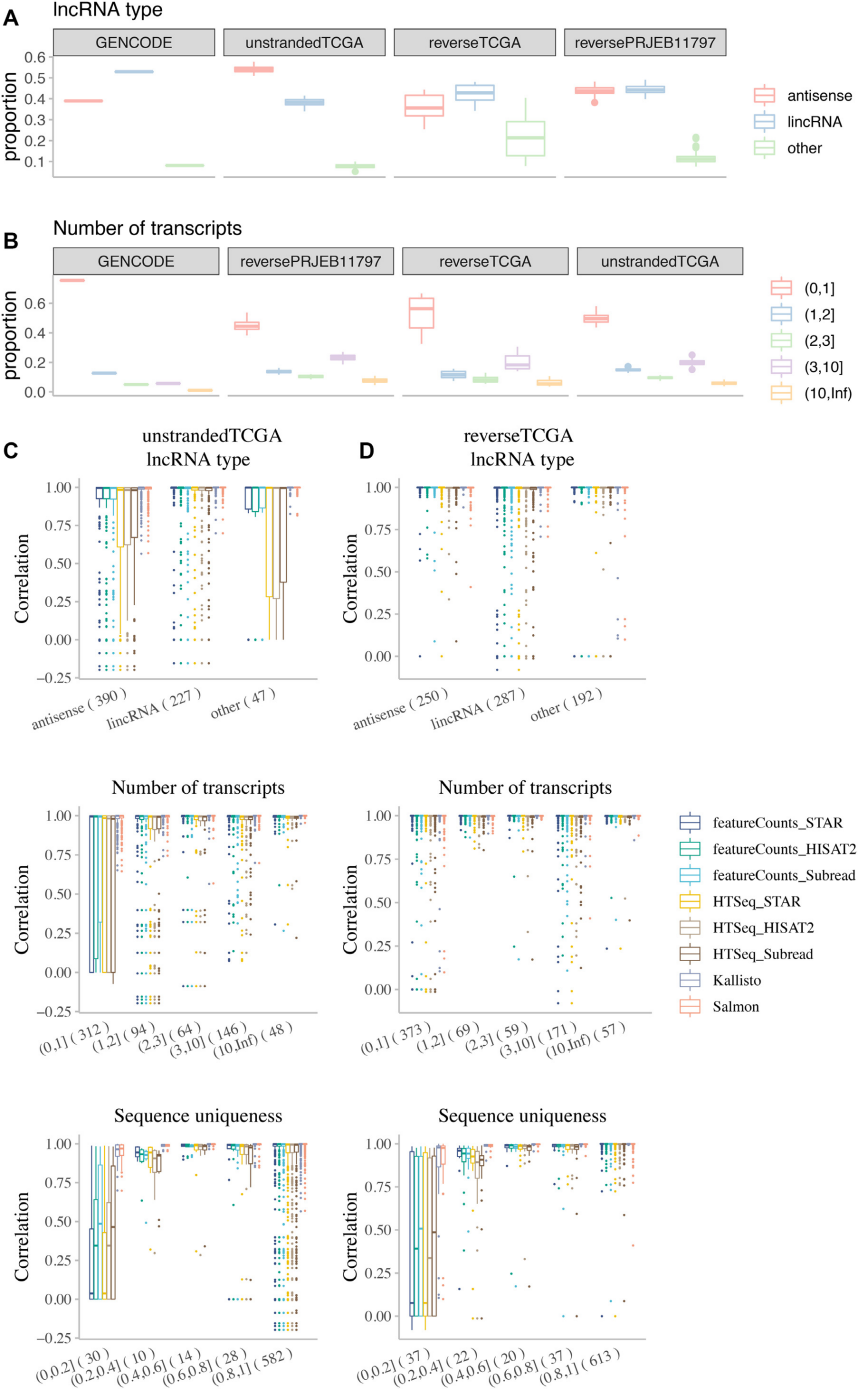
Features of total, expressed, and discordant lncRNAs. The proportion of (A) The lncRNA type and (B) the number of transcripts of lncRNAs in GENCODE and expressed lncRNAs in samples from the 3 datasets. Each point in the box plot represents 1 sample. (C and D) The lncRNA types, number of transcripts, and sequence uniqueness of expressed lncRNAs (median FPKM > 1 in the dataset). Spearman’s correlation was calculated comparing each method and ground truth. Each point in the box plot represents 1 gene. Numbers in brackets in x-axis labels are the number of genes in the category. In the box plots, the top and bottom of the rectangle represent the third and the first quartiles. The band inside the rectangle is the second quartile (the median). The whiskers above and below the box show the upper and lower fences, which are 1.5 times interquartile range above the third quartile, or 1.5 times interquartile range below the first quartile, respectively.

Antisense and long intergenic non-coding RNAs (lincRNAs) are the 2 major types of lncRNAs. In un-stranded samples, the mean proportion of antisense lncRNAs in the expressed lncRNAs is 54%, which is much higher than the proportion of antisense lncRNAs in GENCODE (39%) and the expressed antisense lncRNAs in reverse-stranded libraries (25–48%) (Fig. 5A). More than three-quarters of lncRNAs have only 1 isoform in GENCODE, while they only constitute approximately half of the expressed lncRNAs in the 3 datasets, indicating that lncRNAs with more isoforms are expressed at a higher percentage (Fig. 5B). In addition, shorter lncRNAs (≤1,000 nucleotides) and lncRNAs with 2 exons are expressed at a lower percentage, compared to the distribution in GENCODE (Additional File 9).

We further investigated the features of discordant lncRNAs (Spearman’s correlation <0.7 compared with respect to the ground truth), especially for alignment-based methods, because pseudoalignment methods are highly concordant with the ground truth. In un-stranded samples, the majority of discordant lncRNAs are antisense (Fig. 5C, Additional File 10). Approximately 20–26% of expressed antisense lncRNAs are discordant, while only 7–10% of expressed lincRNA are discordant, indicating that antisense lncRNAs are more susceptible to misquantification from alignment-based methods in un-stranded samples. However, in reverse-stranded samples, the percentage of discordant antisense lncRNAs is <2%, whereas the percentage of discordant lincRNAs is still as high as 4–7% (Fig. 5D, Additional Files 10 and 11A). Therefore, compared to un-stranded RNA-Seq, reversed-stranded protocols are better at the quantification of antisense lncRNAs. The inferior performance of lncRNA quantification in un-stranded samples is further reflected when comparing the number of transcripts, transcript length, number of exons, and sequence uniqueness of expressed and discordant lncRNAs among the 3 datasets (Figs 5C and D, Additional Files 11B–D and 12–15). The difference among the breakdown of these lncRNA features is largely due to inaccurate quantification of antisense lncRNAs in un-stranded samples (Additional File 16). For example, of the 102 discordant lncRNAs with only 1 isoform in un-stranded samples, three-quarters are antisense, and of the 105 discordant lncRNAs with high sequence uniqueness (>80% unique sequences), the majority are antisense. In both cases, the number discordant antisense lncRNAs is <3 in reverse-stranded samples. Nevertheless, lncRNAs with very low sequence uniqueness (<20% unique sequences) are quantified poorly in both un-stranded and reverse-stranded samples. To summarize, antisense RNAs and lncRNAs with very low sequence uniqueness are quantified poorly by alignment-based methods, especially in un-stranded RNA-Seq samples.

### Examples of concordant and discordant lncRNAs

**Figure 6:**
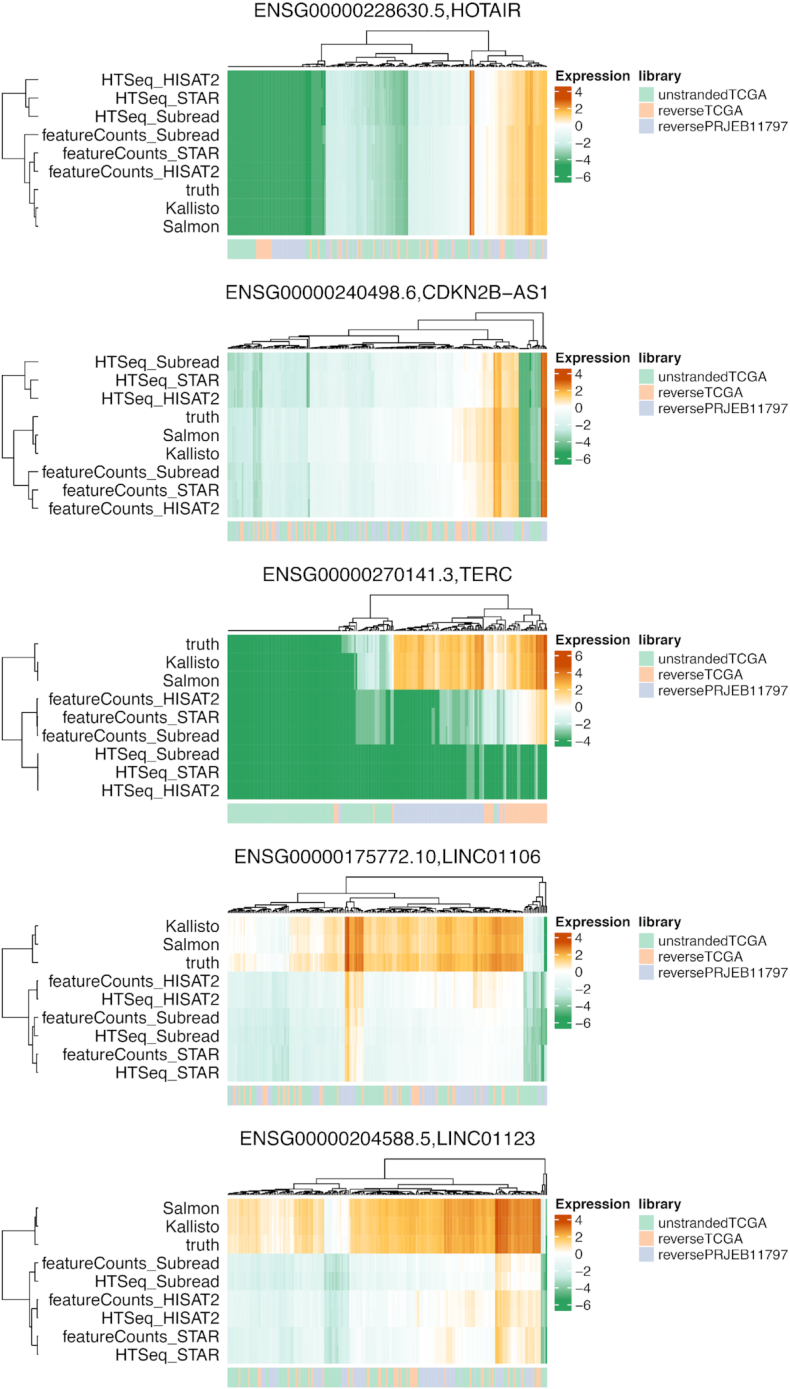
Examples of lncRNAs in cancer. The lncRNAs that were previously reported to play a role in cancer are shown in the 3 datasets. The heat maps show the FPKM value (log transformed) obtained from each method and the ground truth. The Euclidean distance and average linkage were used for clustering.

To demonstrate the importance of accurate lncRNA expression quantification, we investigated the expression profile of a number of well-known lncRNAs with important functions in cancer: HOTAIR, CDKN2B-AS1, TERC, LINC01106, and LINC01123 (Fig. 6). HOTAIR and CDKN2B-AS1 are 2 examples in which all methods perform equally or similarly well, although the expression levels called by Kallisto and Salmon are closer to the ground truth. Note that this is not the case for the other 3 lncRNAs. TERC, involved in cancer progression and risk, was accurately called by Kallisto and Salmon mostly in reverse-stranded datasets, whereas alignment-based methods using featureCounts and HTSeq did not correctly pick up this lncRNA. Similarly, the lincRNA LINC01106, differentially expressed in lung adenocarcinoma and nasopharyngeal carcinoma, and LINC01123, differentially expressed in intrahepatic cholangiocarcinoma, also showed a similar pattern, in which their expression was called accurately by Kallisto and Salmon in both un-stranded and reverse-stranded samples but not by the other methods.

## Discussion

In this work, we compared the performance of popular RNA-Seq processing pipelines for the quantification of gene expression. In particular, we focus on cancer samples and lncRNAs, which have not yet been studied thoroughly in previous RNA-Seq benchmarking studies. An increasing number of studies are using TCGA RNA-Seq data to study lncRNA expression profiles and identify potential lncRNA biomarkers [17,33]. These public resources provide rich opportunities for studying the expression and function of lncRNAs in cancer in a cost-effective way. It is thus critical to choose the right method for accurate expression quantification of lncRNAs.

The 2 pseudoalignment methods, Kallisto and Salmon, outperformed alignment-based gene quantification methods HTSeq and featureCounts at both sample-level and gene-level comparison, regardless of the choice of library type (un-stranded vs reverse-stranded), aligners (STAR, Subread, HISAT2) or transcriptome annotation (GENCODE and NONCODE). Further evaluation of the methods, including RSEM, on datasets generated by Polyester showed that RSEM's performance was similar to that of Kallisto and Salmon. Pseudoalignment methods detected more lncRNAs in each sample, which is similar to those levels in the ground truth for the simulated datasets. They were also highly concordant with the ground truth in terms of having the highest Spearman’s correlation and the lowest Euclidean distance, especially at sample-level comparisons. When each method was linearly regressed with the ground truth, almost all the points from Kallisto and Salmon fell on the diagonal line, with very few outliers (Additional File 5). The superior performance of Kallisto and Salmon could be observed for both un-stranded and reverse-stranded samples, for both lncRNAs and protein-coding genes in GENCODE. Furthermore, it also held true when different transcriptome annotation was used in the analysis, because a similar pattern was observed for lncRNAs in the analysis with GENCODE and NONCODE transcriptome annotation.

**Figure 7:**
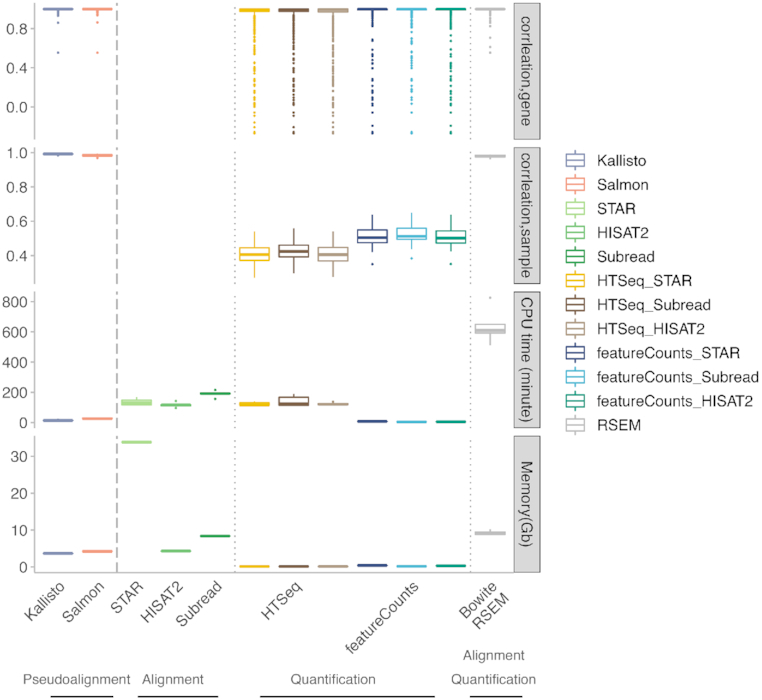
Computational resource use comparison of the tools. The box plot shows the accuracy (Spearman’s correlation in gene- and sample-level comparison with ground truth), CPU time (unit: minutes), and computer memory use (unit: GB) for each tool. The aligners (STAR, HISAT2, and Subread) and the quantification tools (HTSeq and featureCounts) are displayed separately. The CPU time and memory for RSEM count in bowtie, the default aligner integrated by RSEM. In the box plots, the top and bottom of the rectangle represent the third and the first quartiles. The band inside the rectangle is the second quartile (the median). The whiskers above and below the box show the upper and lower fences, which are 1.5 times interquartile range above the third quartile, or 1.5 times interquartile range below the first quartile, respectively.

Because both Kallisto and Salmon performed highly concordantly with the ground truth, they were also highly concordant with each other, as previously reported [34,35]. They cluster together in the method similarity matrix before clustering with the ground truth (Fig. 4). However, Kallisto was faster than Salmon, used less memory (Fig. 7), and performed better at sample- and gene-level comparison when examining Spearman’s correlation, Euclidean distance, mean squared error, and adjusted *R*^2^ values as compared with the ground truth, especially for the 2 reverse-stranded datasets (Additional File 7).

On the contrary, alignment-based methods HTSeq and featureCounts underestimated the expression of lncRNAs. They detected the expression of fewer lncRNA genes and they had far more discordant genes compared to the ground truth. In the simulated datasets, the expressed lncRNAs are mainly antisense and lincRNAs. There are more antisense lncRNAs expressed in the un-stranded samples, compared to the composition of lncRNA types in the GENCODE annotation (Fig. 5A). However, >20% of the expressed antisense lncRNAs in un-stranded samples are discordant, which is much higher than the discordance rate of expressed lincRNAs in the same dataset. This further confirms that un-stranded RNA-Seq protocols do not perform well for expression quantification for overlapping genomic features such as antisense lncRNAs. The quantification of antisense lncRNA expression is improved greatly in reverse-stranded samples, with <2% discordance rate of expressed antisense lncRNAs. The differences observed for the Spearman’s correlation in the breakdown of lncRNA features (e.g., number of transcripts, transcript length, number of exons, etc.) in un-stranded and reverse-stranded datasets can be largely ascribed to the features of the large numbers of discordant expressed antisense lncRNAs in the un-stranded dataset (Fig. 5, Additional File 16). Because antisense lncRNAs contribute to the majority of discordant lncRNAs in un-stranded samples, reverse-stranded protocol is recommended for future RNA-Seq experiments. Alternatively, if only un-stranded samples are available, it becomes vital to choose the right method such as Kallisto or Salmon for the analysis because these 2 pseudoalignment methods are seldom affected by the type of lncRNAs or the type of RNA-Seq protocols and have very few discordant lncRNAs, even for antisense lncRNAs, or lncRNAs with low sequence uniqueness.

When the comparison was restricted to only the alignment-based gene quantification methods (Figs 2 and 3), featureCounts performed slightly better than HTSeq and also took much shorter CPU time (Fig. 7). Subread performed slightly better than the other 2 aligners for un-stranded samples.

It is worth noting that HTSeq is the default workflow for TCGA data stored in the Genomic Data Commons data portal. Because the majority of TCGA RNA-Seq samples were prepared with un-stranded protocol, it is recommended to use pseudoalignment methods for analysis. We have reprocessed the TCGA RNA-Seq datasets using Kallisto with GENCODE annotation (version 27). The results are deposited online for the wider research community studying lncRNAs in TCGA samples, and we also provide a web interface to investigate and visualize gene expression in these samples.

The comparison in this analysis was performed at the gene level. If the goal is to examine transcript-level expression, HTSeq and featureCounts are not suitable for the purpose. They are developed explicitly for gene-level read counts. When they count the reads mapped to transcripts rather than genes, reads mapped to exons shared by several transcripts will then be considered ambiguous and discarded by default. Kallisto, Salmon, and RSEM are able to produce both transcript- and gene-level expression output.

We further addressed the problem of using partial transcriptome annotation (Fig. 1, Additional File 3). Full transcriptome annotation is always recommended for RNA-Seq analysis when it is available to improve accuracy. When studying organisms with poorly annotated transcriptome, it is advisable to assemble and reconstruct the transcriptome first. Furthermore, with the advent of long-read sequencing technologies, more novel transcripts are expected to be identified even for well-annotated transcriptome such as the human. Several methods have been developed for transcriptome assembly and reconstruction, including Cufflinks [36], Trinity [37], TransPS [38], and DRUT [39].

One limitation of this study is that the simulated datasets are based on polyA-selected RNA-Seq. It would be helpful to evaluate RNA-Seq methods that capture more lncRNAs. However, this study focuses on using existing RNA-seq datasets for profiling lncRNAs in cancer. More importantly, in cancer research most of the available datasets, including TCGA, were generated using polyA-selected RNA-Seq. Thus, our study still provides valuable guidelines for researchers studying lncRNAs in cancer. Another limitation is that only simulated data were used in the study. Simulated data may not capture the complexity of real data and true experimental variability. A more comprehensive approach to complement the simulated data with experimental data should be considered in further benchmark studies [40]. More vigorous evaluation using real expression data from other platforms and experimental validations such as reverse transcriptase PCR can be carried out in future.

Reads from the lncRNAs that cannot be quantified accurately by HTSeq and featureCounts are either aligned poorly to the genome, or they can be properly aligned, but HTSeq and featureCounts cannot determine where to assign the reads because of overlapping annotation with other genes (Additional File 17). The superior performance of pseudoalignment methods and RSEM might be due to the expectation maximization algorithm that they deploy, which focuses on the difficulty of accurate quantification for reads that cannot be uniquely aligned to the genome or cannot be uniquely assigned to genes. An RNA-seq experiment can be regarded as the statistical problem of random sampling of subsequences (i.e., reads) from spliced transcripts of different length. Several of these transcripts may share the same exact exons, bringing uncertainty to the reads drawn from those shared exons. Thus, it is important to properly model this statistical problem and to capture and resolve such uncertainty as accurately as possible. We speculate that pseudoalignment methods and RSEM methods are able to model this problem more accurately by iteratively assigning reads to a transcript or a set of transcripts with a certain probability. Other methods using an expectation maximization algorithm such as IsoEM [41] might achieve similar accuracy to pseudoalignment methods and RSEM. Furthermore, the speed improvement achieved by pseudoalignment rather than alignment-based methods (Fig. 7) allows the use of more robust statistical inference techniques such as bootstrapping.

## Potential Implications

In summary, considering the consistency with the ground truth, flexibility at both gene- and transcript-level analysis, and the computational resource use, pseudoalignment methods Kallisto and Salmon are recommended for RNA-Seq analysis for lncRNAs, with Kallisto performing slightly better than Salmon. The full transcriptome annotation including protein-coding genes, lncRNAs, and others is also the recommended strategy for RNA-Seq analysis.

The large amount of data produced by next-generation sequencing techniques has posed great challenges for fast and scalable data analysis. Our study findings imply that for RNA-Seq datasets, incorporating pseudoalignment methods into the analytical framework can achieve high accuracy with minimum computing requirements. Moreover, with more and more RNA-Seq datasets specifically for studying lncRNAs becoming available, our work also lays the basis for a more comprehensive evaluation of tools for lncRNA expression quantification.

## Methods

### Definitions

Here we clarify and define relevant terms. (i) Genes and transcripts: a transcript, sometimes also referred to as an isoform, is composed of exons. An exon is any part of a gene that will encode a part of the final mature RNA. A gene is a collection of transcripts. Transcripts of the same gene often share exons. In this study the analysis was performed at the gene level. (ii) Expressed genes: when describing a single sample, “expressed genes” refer to genes with FPKM ≥ 1 in that particular sample. When describing multiple samples in a dataset, “expressed genes” refer to the genes with median FPKM ≥ 1 across the cohort of samples. (iii) Discordant genes: these are defined as genes whose Spearman’s correlation of FPKM values with the ground truth is <0.7, when compared across a cohort of samples.

### Reference genome and transcriptome

Human transcriptome version GENCODE release 27 (GTF file and transcriptome fasta file) were downloaded from the GENCODE FTP site. The GENCODE release 27 collects 58,288 genes and 200,401 transcripts, among which are 19,836 protein-coding genes (Additional File 1). If lncRNA is defined as non-coding, 3′ overlapping non-coding RNA, antisense, bidirectional promoter lncRNA, lincRNA, macro lncRNA, sense intronic, and sense overlapping, there are 14,168 lncRNAs. The primary assembly of human genome GRCh38 was also downloaded from the same site.

The NONCODE database [2] collecting 172,216 transcripts from 96,308 lncRNA genes (version 5) was downloaded from their website and merged with GENCODE to create a new set of transcriptome annotation. Both the GENCODE version and GENCODE combined with NONCODE version were used to analyze the datasets and simulate 2 sets of ground truth for comparison.

The indexes for Kallisto and Salmon were built using the transcriptome fasta file. The indexes for RSEM and STAR were built using transcriptome GTF file and GRCh38 genome sequences. The indexes for Subread and HISAT2 were built using GRCh38 genome sequences.

Gene features (number of transcripts, number of exons, transcript length) were generated from GTF file using in-house script. Unique *k*-mers of genes were generated using script from Computational Genomics Analysis and Training (CGAT).

### Pipelines

Nine pipelines were applied to process the datasets, including 2 pseudoalignment methods, Kallisto and Salmon; RSEM with bowtie as the aligner; and a combination of read aligners (STAR, Subread, HISAT2) and quantification tools (HTSeq and featureCounts).

Kallisto, version 0.44.0, quant mode. Default parameters were applied. Stand-specific option was set as "–rf-stranded" for reverse-stranded samples.

Salmon, version 0.9.1, quant mode. Default parameters were applied. Stand-specific option: "-l A." Kallisto and Salmon measure the expression level of each transcript by default. To get gene-level expression results, the package tximport [42] was used.

STAR, version 2.5.4a. Two-pass mode was used for mapping.

Subread, version 1.6.1. Stand-specific option was set as "-S ff" for un-stranded samples and "-S rf" for reverse-stranded samples. Other settings: "–multiMapping -B 4 -t 0 ."

HISAT2, version 2.1.0. Stand-specific option was set as "–rna-strandness RF" for reverse-stranded samples.

HTSeq, version 0.7.2. Mapped reads from STAR, Subread, and HISAT2 were counted for each gene according to the GTF file from either GENCODE or GENCODE+NONCODE annotations. Stand-specific option was set as "-s no" for un-stranded samples and "-s yes" for reverse-stranded samples.

featureCounts, version 1.6.1. Mapped reads from STAR, Subread, and HISAT2 were counted for each gene according to the GTF file from either GENCODE or GENCODE+NONCODE annotations. Stand-specific option was set as "-s 2" for reverse-stranded samples. HTSeq and featureCounts output read count for each gene. FPKM values were generated from read counts with in-house scripts.

RSEM, version 1.3.0. The command "rsem-calculate-expression" was used together with bowtie aligner to obtain transcript- and gene-level quantification.

To compare the computational resources required by each tool, RNA-Seq reads from 5 original samples were chosen and processed with each tool, with the number of threads set at 4.

### Sample-level and gene-level comparison

The gene expression measured by each tool was compared with ground truth at both sample level and gene level. In sample-level comparison, gene expression from a single sample was compared with ground truth for each method. In gene-level comparison, gene expression for a single gene across the samples was compared with ground truth for each method.

### Clustering and heat maps

Hierarchical clustering was performed for the sample method matrix. Euclidean distance and average linkage were used for both the columns and rows. The clustering dendrogram was cut into 4 groups and the number of times that every 2 methods are clustered together was counted and used to construct a similarity matrix.

### Statistical tests

The Mann-Whitney *U* test was used to test the difference between 2 continuous variables, and χ^2^ test was used to test the difference of 2 ratios. Unless otherwise specified, all the tests have *P*-values <0.001; thus, they are not explicitly explained in the main text.

### Pan-Cancer RNA-Seq analysis

The raw RNA-Seq sequencing data of TCGA samples were downloaded from the ISB Cancer Genomics Cloud and processed with Kallisto, using GENCODE (version 27) as transcriptome reference.

## Availability of Source Code and Requirements

- Project name: RNASeq_pipeline
- Project home page: https://github.com/gevaertlab/RNASeq_pipeline (licence: MIT)
- Operating system(s): GNU/Linux
- Programming language: Linux/Bash, R, and Python

## Availability of Supporting Data and Materials

The TCGA RNA-Seq re-analysis results and the simulated datasets are available in the Stanford Medicine Box [43].

The web interface for investigating and visualizing individual gene expression can be found in Zheng and Gevaert [44].

Supporting data and code are also available via the *GigaScience* database, GigaDB [45].

## Additional Files

Additional File 1—The genes and transcripts in GENCODE release 27.

Additional File 2—The percentage of expressed genes using each method and different gene annotation sets.

Additional File 3—The effect of incomplete transcriptome annotation on the expression quantification of protein-coding genes.

Additional File 4—The percentage of expressed lncRNA genes using each method and full annotation.

Additional File 5—Examples of sample-level comparison of each method and the ground truth.

Additional File 6—Sample-level comparison of gene expression.

Additional File 7—Statistical tests for gene-level comparison between pseudoalignment methods and alignment-based methods.

Additional File 8—Gene-level comparison of gene expression.

Additional File 9—Features of total and expressed lncRNAs.

Additional File 10—Feature (lncRNA type) of discordant lncRNAs.

Additional File 11—Features of discordant lncRNAs.

Additional File 12—Number of transcripts of discordant lncRNAs.

Additional File 13—Transcript length of discordant lncRNAs.

Additional File 14—Number of exons of discordant lncRNAs.

Additional File 15—Sequence uniqueness of discordant lncRNAs.

Additional File 16—Overall feature breakdown of GENCODE, expressed, and discordant lncRNAs.

Additional File 17—Read mapping of discordant lncRNAs.

giz145_GIGA-D-19-00113_Original_Submission

giz145_GIGA-D-19-00113_Revision_1

giz145_GIGA-D-19-00113_Revision_2

giz145_GIGA-D-19-00113_Revision_3

giz145_GIGA-D-19-00113_Revision_4

giz145_Response_to_Reviewer_Comments_Original_Submission

giz145_Response_to_Reviewer_Comments_Revision_1

giz145_Response_to_Reviewer_Comments_Revision_2

giz145_Response_to_Reviewer_Comments_Revision_3

giz145_Reviewer_1_Report_Original_Submission Bo Li, Ph.D. -- 6/2/2019 Reviewed

giz145_Reviewer_1_Report_Revision_1 Bo Li, Ph.D. -- 10/5/2019 Reviewed

giz145_Reviewer_2_Report_Original_Submission Serghei Mangul -- 6/5/2019 Reviewed

giz145_Reviewer_2_Report_Revision_1 Serghei Mangul -- 10/23/2019 Reviewed

giz145_Reviewer_2_Report_Revision_2 Serghei Mangul -- 11/4/2019 Reviewed

giz145_Reviewer_3_Report_Original_Submission Andrey D. Prjibelski, M.Sc. -- 6/11/2019 Reviewed

giz145_Supplemental_File

## Abbreviations

bp: base pairs; CPU: central processing unit; FPKM: fragments per kilobase million; HOTAIR: Hox transcript antisense RNA; ISB: Institute for Systems Biology; lincRNA: long intergenic non-coding RNA; lncRNA: long non-coding RNA; NCBI: National Center for Biotechnology Information; NIH: National Institutes of Health; RNA-Seq: RNA sequencing; SRA: Sequence Read Archive; TCGA: The Cancer Genome Atlas; TERC: telomerase RNA component; TERT: telomerase reverse transcriptase.

## Competing Interests

The authors declare that they have no competing interests.

## Funding

This work was supported by the National Institute of Dental and Craniofacial Research (NIDCR) (U01 DE025188), the National Institute of Biomedical Imaging and Bioengineering (R01 EB020527), and the National Cancer Institute (U01 CA217851), all of the NIH. The content is solely the responsibility of the authors and does not necessarily represent the official views of the NIH. The funders did not play a role in the design and execution of this study.

## Authors' Contributions

H.Z. and O.G. conceived and designed the study. H.Z., K.B., and M.H. performed data analysis. H.Z. wrote the manuscript, and all authors participated in preparing the manuscript.

## References

[1] Mattick JS, Rinn JL Discovery and annotation of long noncoding RNAs. Nat Struct Mol Biol. 2015;22(1):5. 25565026 10.1038/nsmb.2942

[2] Fang S, Zhang L, Guo J, et al. NONCODEV5: A comprehensive annotation database for long non-coding RNAs. Nucleic Acids Res. 2018;46(D1):D308–14. 29140524 10.1093/nar/gkx1107 PMC5753287

[3] Iyer MK, Niknafs YS, Malik R, et al. The landscape of long noncoding RNAs in the human transcriptome. Nat Genet. 2015;47(3):199. 25599403 10.1038/ng.3192 PMC4417758

[4] Derrien T, Johnson R, Bussotti G, et al. The GENCODE v7 Catalog of Human Long Noncoding RNAs: Analysis of their gene structure, evolution, and expression. Genome Res. 2012;22(9):1775–89. 22955988 10.1101/gr.132159.111 PMC3431493

[5] Fatica A, Bozzoni I Long non-coding RNAs: New players in cell differentiation and development. Nat Rev Genet. 2014;15(1):7. 24296535 10.1038/nrg3606

[6] Esteller M Non-coding RNAs in human disease. Nat Rev Genet. 2011;12(12):861. 22094949 10.1038/nrg3074

[7] Wang KC, Chang HY Molecular mechanisms of long noncoding RNAs. Mol Cell. 2011;43(6):904–14. 21925379 10.1016/j.molcel.2011.08.018 PMC3199020

[8] Schmitt AM, Chang HY Long noncoding RNAs in cancer pathways. Cancer Cell. 2016;29(4):452–63. 27070700 10.1016/j.ccell.2016.03.010 PMC4831138

[9] Huarte M The emerging role of lncRNAs in cancer. Nat Med. 2015;21(11):1253–61. 26540387 10.1038/nm.3981

[10] Gupta RA, Shah N, Wang KC, et al. Long non-coding RNA HOTAIR reprograms chromatin state to promote cancer metastasis. Nature. 2010;464(7291):1071–6. 20393566 10.1038/nature08975 PMC3049919

[11] Zhang J, Zhang P, Wang L, et al. Long non-coding RNA HOTAIR in carcinogenesis and metastasis. Acta Biochim Biophys Sin (Shanghai). 2014;46(1):1–5. 24165275 10.1093/abbs/gmt117 PMC3869294

[12] Yu W, Gius D, Onyango P, et al. Epigenetic silencing of tumour suppressor gene P15 by its antisense RNA. Nature. 2008;451(7175):202–6. 18185590 10.1038/nature06468 PMC2743558

[13] Li XX, Liang XJ, Zhou LY, et al. Analysis of differential expressions of long non-coding RNAs in nasopharyngeal carcinoma using next-generation deep sequencing. J Cancer. 2018;9(11):1943–50. 29896278 10.7150/jca.23481 PMC5995947

[14] Tian Z, Wen S, Zhang Y, et al. Identification of dysregulated long non-coding RNAs/microRNAs/mRNAs in TNM I stage lung adenocarcinoma. Oncotarget. 2017;8(31):51703–18. 28881680 10.18632/oncotarget.18512 PMC5584281

[15] Yang W, Li Y, Song X, et al. Genome-Wide analysis of long noncoding RNA and mRNA co-expression profile in intrahepatic cholangiocarcinoma tissue by RNA sequencing. Oncotarget. 2017;8(16):26591–9. 28427159 10.18632/oncotarget.15721 PMC5432281

[16] Su X, Malouf GG, Chen Y, et al. Comprehensive analysis of long non-coding RNAs in human breast cancer clinical subtypes. Oncotarget. 2014;5(20):9864. 25296969 10.18632/oncotarget.2454 PMC4259443

[17] Yan X, Hu Z, Feng Y, et al. Comprehensive genomic characterization of long non-coding RNAs across human cancers. Cancer Cell. 2015;28(4):529–40. 26461095 10.1016/j.ccell.2015.09.006 PMC4777353

[18] Zhao S, Zhang Y, Gordon W, et al. Comparison of stranded and non-stranded RNA-Seq transcriptome profiling and investigation of gene overlap. BMC Genomics. 2015;16:675. 26334759 10.1186/s12864-015-1876-7 PMC4559181

[19] Sigurgeirsson B, Emanuelsson O, Lundeberg J Analysis of stranded information using an automated procedure for strand specific RNA sequencing. BMC Genomics. 2014;15:631. 25070246 10.1186/1471-2164-15-631 PMC4247151

[20] Everaert C, Luypaert M, Maag JLV, et al. Benchmarking of RNA-sequencing analysis workflows using whole-transcriptome RT-qPCR expression data. Sci Rep. 2017;7(1):1559. 28484260 10.1038/s41598-017-01617-3 PMC5431503

[21] Teng M, Love MI, Davis CA, et al. A benchmark for RNA-Seq quantification pipelines. Genome Biol. 2016;17:74. 27107712 10.1186/s13059-016-0940-1 PMC4842274

[22] Bray NL, Pimentel H, Melsted P, et al. Near-optimal probabilistic RNA-Seq ouantification. Nat Biotechnol. 2016;34(5):525–7. 27043002 10.1038/nbt.3519

[23] Patro R, Duggal G, Love MI, et al. Salmon provides fast and bias-aware quantification of transcript expression. Nat Methods. 2017;14(4):417–9. 28263959 10.1038/nmeth.4197 PMC5600148

[24] Li B, Dewey CN RSEM: Accurate transcript quantification from RNA-Seq data with or without a reference genome. BMC Bioinformatics. 2011;12:323. 21816040 10.1186/1471-2105-12-323 PMC3163565

[25] Anders S, Pyl PT, Huber W HTSeq–a Python framework to work with high-throughput sequencing data. Bioinformatics. 2015;31(2):166–9. 25260700 10.1093/bioinformatics/btu638 PMC4287950

[26] Liao Y, Smyth GK, Shi W featureCounts: An efficient general purpose program for assigning sequence reads to genomic features. Bioinformatics. 2014;30(7):923–30. 24227677 10.1093/bioinformatics/btt656

[27] Dobin A, Davis CA, Schlesinger F, et al. STAR: Ultrafast universal RNA-Seq aligner. Bioinformatics. 2013;29(1):15–21. 23104886 10.1093/bioinformatics/bts635 PMC3530905

[28] Liao Y, Smyth GK, Shi W The Subread Aligner: Fast, accurate and scalable read mapping by seed-and-vote. Nucleic Acids Res. 2013;41(10):e108. 23558742 10.1093/nar/gkt214 PMC3664803

[29] Kim D, Langmead B, Salzberg SL HISAT: A fast spliced aligner with low memory requirements. Nat Methods. 2015;12(4):357–60. 25751142 10.1038/nmeth.3317 PMC4655817

[30] Krueger F Trim Galore: A wrapper tool around Cutadapt and FastQC to consistently apply quality and adapter trimming to FastQ files. 2015; http://www.bioinformatics.babraham.ac.uk/projects/trim_galore/. Access date: April 13th, 2017

[31] Frazee AC, Jaffe AE, Langmead B, et al. Polyester: Simulating RNA-Seq datasets with differential transcript expression. Bioinformatics. 2015;31(17):2778–84. 25926345 10.1093/bioinformatics/btv272 PMC4635655

[32] Maag JL, Fisher OM, Levert-Mignon A, et al. Novel aberrations uncovered in Barrett’s esophagus and esophageal adenocarcinoma using whole transcriptome sequencing. Mol Cancer Res. 2017;15(11):1558–69. 28751461 10.1158/1541-7786.MCR-17-0332

[33] Zeng JH, Liang L, He RQ, et al. Comprehensive investigation of a novel differentially expressed lncRNA expression profile signature to assess the survival of patients with colorectal adenocarcinoma. Oncotarget. 2017;8(10):16811–28. 28187432 10.18632/oncotarget.15161 PMC5370003

[34] Zhang C, Zhang B, Lin LL, et al. Evaluation and comparison of computational tools for RNA-Seq isoform quantification. BMC Genomics. 2017;18(1):583. 28784092 10.1186/s12864-017-4002-1 PMC5547501

[35] Jin H, Wan YW, Liu Z. Comprehensive evaluation of RNA-Seq quantification methods for linearity. BMC Bioinformatics. 2017;18(4):117. 28361706 10.1186/s12859-017-1526-y PMC5374695

[36] Trapnell C, Roberts A, Goff L, et al. Differential gene and transcript expression analysis of RNA-Seq experiments with TopHat and Cufflinks. Nat Protoc. 2012;7(3):562–78. 22383036 10.1038/nprot.2012.016 PMC3334321

[37] Grabherr MG, Haas BJ, Yassour M, et al. Full-length transcriptome assembly from RNA-Seq data without a reference genome. Nat Biotechnol. 2011;29(7):644–52. 21572440 10.1038/nbt.1883 PMC3571712

[38] Liu M, Adelman ZN, Zhang L TransPS: A transcriptome post scaffolding method for assembling high quality contigs. Comput Biol J. 2014;2014, doi:10.1155/2014/961823. PMC5333928 28261602

[39] Mangul S, Caciula A, Glebova O, et al. Improved transcriptome quantification and reconstruction from RNA-Seq reads using partial annotations. In Silico Biol. 2011;11(5–6):251–61. 23202426 10.3233/ISB-2012-0459

[40] Mangul S, Martin LS, Hill BL, et al. Systematic benchmarking of omics computational tools. Nat Commun. 2019;10(1):1393. 30918265 10.1038/s41467-019-09406-4 PMC6437167

[41] Nicolae M, Mangul S, Măndoiu II, et al. Estimation of alternative splicing isoform frequencies from RNA-Seq data. Algorithms Mol Biol. 2011;6(1):9. 21504602 10.1186/1748-7188-6-9 PMC3107792

[42] Soneson C, Love MI, Robinson MD Differential analyses for RNA-Seq: Transcript-level estimates improve gene-level inferences. F1000Res. 2015;4, doi:10.12688/f1000research.7563.2. PMC4712774 26925227

[43] Zheng H, Gevaert O Supporting data for “Benchmark of long non-coding RNA quantification for RNA sequencing of cancer samples.”. Stanford Medicine Box 2019 https://stanfordmedicine.box.com/s/lu703xuaulfz02vgd2lunxnvt4mfvo3q.Access date: Dec. 3rd, 2019 10.1093/gigascience/giz145 PMC6897288 31808800

[44] Zheng H, Gevaert O Expression profiling and analysis of lncRNAs in TCGA. Gevaert Lab, Stanford University 2019 http://apps.gevaertlab.stanford.edu/. Access date: Dec. 3rd, 2019

[45] Zheng H, Brennan K, Hernaez M, et al. Supporting data for “Benchmark of long non-coding RNA quantification for RNA sequencing of cancer samples.”. GigaScience Database. 2019 10.5524/100671. PMC6897288 31808800

