# Supplementary material for: Benchmark of long non-coding RNA quantification for RNA sequencing of cancer samples
Source: Gigascience. 2019 Dec 6;8(12):giz145. doi: 10.1093/gigascience/giz145 (PMC6897288; doi:10.1093/gigascience/giz145)
Supplement: giz145_GIGA-D-19-00113_Original_Submission [file giz145_giga-d-19-00113_original_submission.pdf]

GigaScience

Benchmark of lncRNA Quantification for RNA-Seq of Cancer Samples

--Manuscript Draft--

Manuscript Number:	GIGA-D-19-00113
Full Title:	Benchmark of lncRNA Quantification for RNA-Seq of Cancer Samples
Article Type:	Research
Funding Information:	National Institute of Dental and Craniofacial Research (US) (U01 DE025188) — Dr Olivier Gevaert<br>National Institute of Biomedical Imaging and Bioengineering (R01 EB020527) — Dr Olivier Gevaert<br>National Cancer Institute (US) (U01 CA217851) — Dr Olivier Gevaert
Abstract:	Background:<br>Long non-coding RNAs (lncRNAs) are emerging as important regulators of various biological processes. While many studies have exploited public resources such as RNA-Seq data in The Cancer Genome Atlas (TCGA) to study lncRNAs in cancer, it is crucial to choose the optimal method for accurate expression quantification.<br><br>Results:<br>In this study, we compared the performance of pseudoalignment methods Kallisto and Salmon, and alignment-based methods HTSeq and featureCounts, in combination with read aligners STAR, Subread, and HISAT2, in lncRNA quantification, by applying them to both un-stranded and reversed-stranded RNA-Seq datasets. Full transcriptome annotation, including protein-coding and noncoding RNAs, greatly improves the specificity of lncRNA expression quantification. Pseudoalignment methods outperform alignment-based methods for lncRNA quantification at both sample- and gene-level comparison, regardless of RNA-Seq protocol type, choice of aligners and transcriptome annotation. Pseudoalignment methods detect more lncRNAs than alignment-based methods and correlate highly with simulated ground truth. On the contrary, alignment-based methods often underestimate lncRNA expression. Antisense lncRNAs are poorly quantified by alignment-based methods, which can be improved using reverse-stranded protocols and pseudoalignment methods.<br><br>Conclusions:<br>Considering the consistency with ground truth and computational resources, pseudoalignment methods Kallisto or Salmon in combination with full transcriptome annotation is our recommended strategy for RNA-Seq analysis for lncRNAs.
Corresponding Author:	Olivier Gevaert<br><br>UNITED STATES
Corresponding Author Secondary Information:	
Corresponding Author's Institution:	
Corresponding Author's Secondary Institution:	
First Author:	Hong Zheng
First Author Secondary Information:	
Order of Authors:	Hong Zheng<br>Kevin Brennan<br>Mikel Hernaez<br>Olivier Gevaert

Powered by Editorial Manager® and ProduXion Manager® from Aries Systems Corporation

Order of Authors Secondary Information:	
**Additional Information:**	
**Question**	**Response**
Are you submitting this manuscript to a special series or article collection?	No
**Experimental design and statistics**<br><br>Full details of the experimental design and statistical methods used should be given in the Methods section, as detailed in our Minimum Standards Reporting Checklist. Information essential to interpreting the data presented should be made available in the figure legends.<br><br>Have you included all the information requested in your manuscript?	Yes
**Resources**<br><br>A description of all resources used, including antibodies, cell lines, animals and software tools, with enough information to allow them to be uniquely identified, should be included in the Methods section. Authors are strongly encouraged to cite Research Resource Identifiers (RRIDs) for antibodies, model organisms and tools, where possible.<br><br>Have you included the information requested as detailed in our Minimum Standards Reporting Checklist?	Yes
**Availability of data and materials**<br><br>All datasets and code on which the conclusions of the paper rely must be either included in your submission or deposited in publicly available repositories (where available and ethically appropriate), referencing such data using a unique identifier in the references and in the “Availability of Data and Materials” section of your manuscript.	Yes

Powered by Editorial Manager® and ProduXion Manager® from Aries Systems Corporation

Have you have met the above requirement as detailed in our Minimum Standards Reporting Checklist?	

Powered by Editorial Manager® and ProduXion Manager® from Aries Systems Corporation

Click here to download Manuscript Template_for_GigaScience_Journal_Manuscript_Submissions.

OXFORD (GIGA)n SCIENCE

GigaScience, 2019, 1–8

doi: xx.xxxx/xxxx
Manuscript in Preparation
Paper

PAPER

Benchmark of lncRNA Quantification for RNA-Seq of Cancer Samples

Hong Zheng[1], Kevin Brennan[1], Mikel Hernaez[2] and Olivier Gevaert[1,3,*]

[1]Stanford Center for Biomedical Informatics Research, Stanford University and [2]Carl R. Woese Institute for Genomic Biology, University of Illinois at Urbana-Champaign and [3]Department of Medicine, and Department of Biomedical Data Science, Stanford University

[*]Correspondence: ogevaert@stanford.edu

Abstract

Background Long non-coding RNAs (lncRNAs) are emerging as important regulators of various biological processes. While many studies have exploited public resources such as RNA-Seq data in The Cancer Genome Atlas (TCGA) to study lncRNAs in cancer, it is crucial to choose the optimal method for accurate expression quantification.
Results In this study, we compared the performance of pseudoalignment methods Kallisto and Salmon, and alignment-based methods HTSeq and featureCounts, in combination with read aligners STAR, Subread, and HISAT2, in lncRNA quantification, by applying them to both un-stranded and reversed-stranded RNA-Seq datasets. Full transcriptome annotation, including protein-coding and noncoding RNAs, greatly improves the specificity of lncRNA expression quantification. Pseudoalignment methods outperform alignment-based methods for lncRNA quantification at both sample- and gene-level comparison, regardless of RNA-Seq protocol type, choice of aligners and transcriptome annotation. Pseudoalignment methods detect more lncRNAs than alignment-based methods and correlate highly with simulated ground truth. On the contrary, alignment-based methods often underestimate lncRNA expression. Antisense lncRNAs are poorly quantified by alignment-based methods, which can be improved using reverse-stranded protocols and pseudoalignment methods.
Conclusions Considering the consistency with ground truth and computational resources, pseudoalignment methods Kallisto or Salmon in combination with full transcriptome annotation is our recommended strategy for RNA-Seq analysis for lncRNAs.

Key words: lncRNA; RNA-Seq; Pseudoalignment

Background

Long non-coding RNAs (lncRNAs) are a diverse class of RNA molecules that are more than 200 nucleotides in length and do not encode proteins [1]. While functional classification is lacking for most lncRNAs, based on their genomic proximity to protein-coding genes and the direction of transcription, lncRNA are often classified into antisense, intronic, bidirectional, intergenic, or overlapping RNAs [1]. GENCODE, the database that provides annotations for human genes and transcripts, defines over 14,000 human lncRNA genes (release 27, https://www.gencodegenes.org). Other lncRNAs databases including NONCODE [2] and MiTranscriptome [3] both collect over 60k lncRNAs. Compared with protein-coding genes, lncRNAs are shorter, lower-expressed, less evolutionarily conserved, and expressed in a more tissue-specific manner [4]. lncRNAs have recently emerged as an essential class of regulatory elements for many biological processes including imprinting, cell differentiation, and development [5]. They are often disrupted in human diseases including cancer [6]. They may interact with DNA, RNA, and proteins, and exert regulatory roles through a variety of mechanisms. Based on their

Compiled on: April 9, 2019.
Draft manuscript prepared by the author.

molecular functions, lncRNA may act as: i) signals, which are indicators of transcriptional activity; ii) decoys, which bind to and titrate away protein targets such as transcription factors; iii) guides, which direct regulatory complexes or transcription factors to specific targets and regulate gene expression in cis or trans, and iv) scaffolds, which serve as central platforms where relevant molecular components in cells are assembled [7].

lncRNAs have been shown to be important in the pathogenesis of human diseases, especially in cancer, and many cancer-relevant lncRNAs have been identified [8, 9]. For example, Hox transcript antisense RNA (HOTAIR), one of the most well-characterized lncRNAs, promotes breast cancer metastasis through recruitment of Polycomb chromatin remodeling complex to silence the HOXD gene cluster [10]. In addition, HOTAIR is overexpressed in breast, liver, lung, and pancreatic cancers [11]. CDKN2B-AS1, an antisense lncRNA encoded by the CDKN2B locus, epigenetically silences nearby tumor suppresser genes and promotes oncogenesis [12]. Telomerase RNA component (TERC), the critical RNA component of telomerase polymerase, serves as a template for the enzyme telomerase reverse transcriptase (TERT) to elongate telomeres. Variants and copy number changes at the TERC locus have been associated with cancer risk and progression [8]. The lncRNA LINC01106 is shown to be differentially expressed in multiple cancer types including lung adenocarcinoma and nasopharyngeal carcinoma [13, 14]. Another lncRNA, LINC01123, is among the five most significantly up-regulated lncRNAs in intrahepatic cholangiocarcinoma [15].

The discovery of oncogenic and tumor suppressor lncRNAs has led to an increased interest in the investigation of lncRNAs as potential cancer drug targets and biomarkers. Hence, it is critical to accurately determine lncRNA expression in cancer research. RNA sequencing (RNA-Seq) has been widely used for massive-parallel gene expression quantification. There have been many studies that explore lncRNA expression profile in cancer using publically available RNA-Seq datasets such as those generated by The Cancer Genome Atlas (TCGA), which provide a rich source of lncRNA expression data in large cancer patient populations [16, 17]. Among those studies, the analysis of the lncRNA expression profile of breast cancer samples in TCGA revealed different subtypes of breast cancer and subtype-specific over-expression of HOTAIR [16]. The analysis of 13 cancer types in TCGA revealed highly cancer site-specific lncRNA expression and dysregulation [17].

There are two types of RNA-Seq protocols, depending on whether strand specificity information of transcripts is retained or not in the library preparation step [18]. The standard protocol loses the information on which strand the original mRNA template is coming from, which makes it difficult to accurately determine gene expression from overlapping genes. The strand specific RNA-Seq protocol, such as the dUTP method, retains strand origin of transcripts by degrading the second strand in the cDNA synthesis step. It has been shown to be more reliable in gene expression quantification and is recommended over the standard protocol [19]. However, the majority of TCGA samples were prepared with standard RNA-Seq protocol.

Multiple tools for processing RNA-Seq data have been developed in recent years. While some studies have benchmarked RNA-Seq analysis workflows [20, 21], their focus has been primarily on protein-coding genes. There is no accepted gold standard pipeline yet that shows which method performs best to quantify expression of lncRNAs. As the interest in studying lncRNAs in cancer grows, it is necessary to determine which algorithms perform best in lncRNA expression quantification, as it is important to understand the differences and limitations of each of them and to follow the best practice of RNA-Seq analysis.

Because of the lower expression and different properties of lncRNAs with respect to protein-coding genes, we hypothesized that the processing and analysis of RNA-Seq data for lncRNA expression may be subjected to different technical biases and challenges, and that special considerations may be necessary to optimize the pipeline specifically for lncRNAs.

To investigate the performance of different methods on the quantification of lncRNAs as well as the effect of different RNA-Seq library preparation protocols, we applied four popular quantification methods, Kallisto [22], Salmon [23], HTSeq [24], and featureCounts [25], on RNA-Seq samples prepared using a standard protocol (i.e. un-stranded) and a strand specific protocol (i.e. reverse-stranded). Kallisto and Salmon are so-called pseudoalignment methods as they do not align sequencing reads to the reference genome; instead, they use expectation-maximization algorithm to iteratively assign reads to a set of compatible transcripts to obtain the estimated abundances for all transcripts. The alignment-free feature makes pseudoalignment methods much faster than alignment-based methods like HTSeq and featureCounts, since the latter require mapping of the sequencing reads to the genome, which takes significant amount of time and computational resources. For alignment-based methods, we used three aligners, STAR [26], Subread [27], and HISAT2 [28] to map the reads to the genome, before applying HTSeq and featureCounts to count the reads mapped to individual genes.

Data Description

Both un-stranded and reverse-stranded RNA-Seq data from TCGA samples were downloaded from ISB Cancer Genomics Cloud (ISB-CGC). The other reverse-stranded dataset was downloaded from NCBI Sequence Read Archive (SRA) under the accession PRJEB11797. Reads QC were performed with Trim galore [29], with the setting '-q 20 -stringency 3 -gzip -length 20 -paired'. Afterwards the reads were mapped to the human transcriptome (both GENCODE and GENCODE combined with NONCODE) by STAR, and were further processed by RSEM [30] (version 1.3.0) to obtain gene and transcript expression. Stand-specific option was set as '-forward-prob 0.5' for un-stranded samples and '-forward-prob 0' for reverse-stranded samples. Simulation of RNA-Seq reads was performed with the RSEM command rsem-simulate-reads, which takes as input abundance estimates, sequencing model parameters, and reference transcripts. The abundance estimates and sequencing model are obtained by running RSEM on a real data set. The TPM values of the transcripts/isoforms are used for the simulation. The total number of simulated reads for each sample is 60 million. The simulated samples with pre-defined gene expression levels serve as the 'ground truth' for the evaluation of other pipelines.

Analyses

Full transcriptome annotation improves the specificity of RNA quantification

We used RSEM [30] to simulate RNA sequencing reads based on three RNA-Seq datasets: 1) 100 un-stranded samples from 10 cancer types in TCGA; 2) 40 reverse-stranded samples in TCGA; 3) 62 reverse-stranded samples from a study of Barrett's Esophagus and Esophageal Adenocarcinoma (PRJEB11797) [31]. To evaluate the effect that different transcriptome annotations has on the quantification of gene expression, we built three transcriptome annotation sets: 1) full annotation with all 58,288 genes in GENCODE release 27; 2) partial annotation

containing only the 19,836 protein-coding genes; and 3) partial annotation with only the 14,168 lncRNAs (Additional file 1).

Using the lncRNA-only annotation over-estimates lncRNA expression compared to full annotation (Figure 1, Additional file 2). The over-estimation effect using an incomplete transcriptome annotation set can be observed for all the methods when using either un-stranded or reverse-stranded RNA-Seq libraries, although the effect is less drastic for alignment-based methods when using reverse-stranded libraries. The effect of incomplete transcriptome annotation is less obvious for protein-coding genes, but there is still a slight increase of the percentage of expressed genes when using only protein-coding annotation, compared to full annotation (Additional file 2, Additional file 3). Thus, using a full annotation improves the specificity of RNA quantification; therefore, it was used in the following analysis.

Pseudoalignment methods outperform alignment-based methods for lncRNA expression quantification

Pseudoalignment methods detect expression of more genes than alignment-based methods (Figure 2A, Additional file 4). The average percentage of expressed lncRNAs (fragments per kilobase million (FPKM)>=1 in ground truth) in the simulated ground truth ranges between 4.68% and 7.4% for the three RNA-Seq datasets, which is very close to the output of Kallisto and Salmon. The alignment-based methods detect less lncRNAs compared to the ground truth, especially for un-stranded samples.

The performance of each method was further evaluated at both sample-level (Figure 2B) and gene-level (Figure 2C). For sample-level evaluation, only expressed lncRNAs (FPKM>=1 in the ground truth) were kept in each sample. The concordance of each method with the ground truth was measured by means of Spearman's correlation, Euclidean distance and linear regression. Gene expression from Kallisto and Salmon yields the highest Spearman's correlation and the lowest Euclidean distance with respect to the ground truth. The two pseudoalignment methods also have the highest level of fitness to the ground truth, in terms of the lowest mean squared error, the highest adjusted R-squared value, and having a slope value of close to one (Figure 2B, Additional file 5). A similar trend can also be observed for protein-coding genes in GENCODE (Additional file 6A). For gene-level evaluation, a comparison was performed using, for each corresponding dataset, only those lncRNAs with median FPKM larger than one in the ground truth, since genes with low read counts are likely to be noise and unlikely to yield reliable results, The number of lncRNAs examined ranges between 464 and 729 for the three RNA-Seq datasets. Kallisto and Salmon perform better than alignment-based methods in terms of higher Spearman's correlation, lower Euclidean distance to the ground truth, linear regression slope closer to one, and higher adjusted R-squared value (Figure 2C, Additional file 7). A similar trend can also be observed for protein-coding genes in GENCODE (Additional file 8A).

For each of the expressed lncRNAs in any of the three datasets, hierarchical clustering was performed to evaluate the similarity of each method's measurement to the ground truth and between each other (Figure 3). Kallisto and Salmon often clustered together with the ground truth. In addition, the three pipelines using featureCounts form another cluster, while pipelines using HTSeq loosely cluster together.

Next, we expanded our analysis and also included lncRNAs from NONCODE, a database collecting 172,216 transcripts from 96,308 lncRNA genes (version 5) [2]. We simulated RNA sequencing reads based on both GENCODE and NONCODE gene annotations and replicated the analysis for lncRNAs in NONCODE. Similar to the results from GENCODE annotation, the two pseudoalignment methods outperform alignment-based methods in both sample-level (Additional file 6B) and gene-level comparison (Additional file 8B).

Characteristics of expressed and discordant lncRNAs

Antisense and long intergenic noncoding RNAs (lincRNAs) are the two major types of lncRNAs. In un-stranded samples, the average proportion of antisense lncRNAs in the expressed lncRNAs is 54%, which is much higher than the proportion of antisense lncRNAs in GENCODE (39%) and the expressed antisense lncRNAs in reverse-stranded libraries (25-48%) (Figure 4A). Over three quarters of lncRNAs have only one isoform in GENCODE, while they only constitute approximately half of the expressed lncRNAs in the three datasets, indicating lncRNAs with more isoforms are expressed at a higher percentage (Figure 4B). In addition, shorter lncRNAs (<=1000nt) and lncRNAs with two exons are expressed at a lower percentage, compared to the distribution in GENCODE (Additional file 9).

We further investigated the features of discordant lncRNAs (spearman's correlation below 0.7 compared with respect to the ground truth), especially for alignment-based methods, since pseudoalignment methods are highly concordant with the ground truth. In un-stranded samples, the majority of discordant lncRNAs are antisense (Figure 4C, Additional file 10). About 20-26% of expressed antisense lncRNAs are discordant, while only 7-10% of expressed lincRNA are discordant, indicating that antisense lncRNAs are more susceptible to misquantification from alignment-based methods in un-stranded samples. However, in reverse-stranded samples, the percentage of discordant antisense lncRNAs is below 2%, whereas the percentage of discordant lincRNAs is still as high as 4-7% (Figure 4D, Additional file 11A, Additional file 10). Therefore, compared to un-stranded RNA-Seq, reversed-stranded protocols are better at the quantification of antisense lncRNAs. The inferior performance of lncRNA quantification in un-stranded samples is further reflected when comparing the number of transcripts, transcript length, number of exons, and sequence uniqueness of expressed and discordant lncRNAs among the three datasets (Figure 4C-D, Additional file 11B-D, Additional files 12-15). The difference among the breakdown of these lncRNA features is largely due to inaccurate quantification of antisense lncRNAs in un-stranded samples (Additional file 16). For example, out of the 102 discordant lncRNAs with only one isoform in un-stranded samples, three quarters of them are antisense, and out of the 105 discordant lncRNAs with high sequence uniqueness (over 80% unique sequences), the majority of them are antisense. In both cases, the number discordant antisense lncRNAs is below three in reverse-stranded samples. Nevertheless, lncRNAs with very low sequence uniqueness (less than 20% unique sequences) are quantified poorly in both un-stranded and reverse-stranded samples. To summarise, antisense RNAs and lncRNAs with very low sequence uniqueness are quantified poorly by alignment-based methods, especially in un-stranded RNA-Seq samples.

Examples of concordant and discordant lncRNAs

To demonstrate the importance of accurate lncRNA expression quantification, we investigated the expression profile of a number of well-known lncRNAs with important functions in cancer: HOTAIR, CDKN2B-AS1, TERC, LINC01106, and LINC01123 (Figure 5). HOTAIR and CDKN2B-AS1 are two examples where all methods perform equally or similarly well, although the expression levels called by Kallisto and Salmon are closer to

the ground truth. Note that this is not the case for the other three lncRNAs. TERC, involved in cancer progression and risk, was accurately called by Kallisto and Salmon mostly in reverse-stranded datasets, whereas alignment-based methods using featureCounts and HTSeq did not correctly pick up this lncRNA. Similarly, the lincRNA LINC01106, differentially expressed in lung adenocarcinoma and nasopharyngeal carcinoma, and LINC01123, differentially expressed in intrahepatic cholangiocarcinoma, also showed a similar pattern, where their expression was called accurately by Kallisto and Salmon in both un-stranded and reverse-stranded samples, but not by the other methods.

Discussion

In this work, we compared the performance of popular RNA-Seq processing pipelines for the quantification of gene expression. In particular, we focus on cancer samples and lncRNAs, which have not yet been studied thoroughly in previous RNA-Seq benchmarking studies. An increasing number of studies are utilizing TCGA RNA-Seq data to study lncRNA expression profiles and identify potential lncRNA biomarkers [17, 32]. These public resources provide rich opportunities for studying the expression and function of lncRNAs in cancer in a cost-effective way. It is thus critical to choose the right method for accurate expression quantification of lncRNAs.

In the three datasets analyzed in this study, the two pseudoalignment methods Kallisto and Salmon outperform alignment-based methods at both sample-level and gene-level comparison, regardless of the choice of library type (un-stranded vs. reverse-stranded), aligners (STAR, Subread, HISAT2) or transcriptome annotation (GENCODE and NONCODE). Kallisto and Salmon detect more lncRNAs in each sample, which is similar to those levels in the ground truth for the simulated datasets. They are also highly concordant with the ground truth in terms of having the highest Spearman's correlation and the lowest Euclidean distance, especially at sample-level comparisons. When linearly regressing each method with the ground truth, almost all the points from Kallisto and Salmon fall on the diagonal line, with very few outliers (Additional file 5). The superior performance of Kallisto and Salmon can be observed for both un-stranded and reverse-stranded samples, for both lncRNAs and protein-coding genes in GENCODE. Furthermore, it also holds true when different transcriptome annotation is used in the analysis, since a similar pattern was observed for lncRNAs in the analysis with GENCODE and NONCODE transcriptome annotation.

Since both Kallisto and Salmon perform highly concordantly with the ground truth, they are also highly concordant between each other, as previously reported [33, 34]. They cluster together in the method similarity matrix before clustering with the ground truth (Figure 3). However, Kallisto is faster than Salmon, utilizes less memory (Additional file 17) and performs better at sample- and gene-level comparison when examining Spearman's correlation, Euclidean distance, mean squared error and adjusted R squared values as compared with the ground truth, especially for the two reverse-stranded datasets (Additional file 7).

On the contrary, alignment-based methods HTSeq and featureCounts underestimate the expression of lncRNAs. They detect the expression of less lncRNA genes and they have a much higher number of discordant genes compared to the ground truth. In the simulated datasets, the expressed lncRNAs are mainly antisense and lincRNAs. There are more antisense lncRNAs expressed in the un-stranded samples, compared to the composition of lncRNA types in the GENCODE annotation (Figure 4A). However, over 20% of the expressed antisense lncRNAs in un-stranded samples are discordant, which is much higher than the discordance rate of expressed lincRNAs in the same dataset. This further confirms that un-stranded RNA-Seq protocols do not perform well for expression quantification for overlapping genomic features such as antisense lncRNAs. The quantification of antisense lncRNA expression is improved greatly in reverse-stranded samples, with less than 2% discordance rate of expressed antisense lncRNAs. The differences observed for the Spearman's correlation in the breakdown of lncRNA features (number of transcripts, transcript length, number of exons, etc.) in un-stranded and reverse-stranded datasets can be largely ascribed to the features of the large numbers of discordant expressed antisense lncRNAs in the un-stranded dataset (Figure 4, Additional file 16). Since antisense lncRNAs contribute to the majority of discordant lncRNAs in un-stranded samples, reverse-stranded protocol is recommended for future RNA-Seq experiments. Alternatively, if only un-stranded samples are available, it becomes vital to choose the right method like Kallisto or Salmon for the analysis, since these two pseudoalignment methods are seldom affected by the type of lncRNAs or the type of RNA-Seq protocols and have very few number of discordant lncRNAs, even for antisense lncRNAs, or lncRNAs with low sequence uniqueness.

It is worth noting that HTSeq is the default workflow for TCGA data stored in GDC data portal. Since the majority of TCGA RNA-Seq samples were prepared with un-stranded protocol, it is recommended to use pseudoalignment methods for analysis. We have reprocessed the TCGA RNA-Seq datasets using Kallisto with GENCDOE annotation (version 27). The results are deposited online for the wider research community studying lncRNAs in TCGA samples and we also provide a web interface to investigate and visualize gene expression in these samples.

Next, the comparison in this analysis was performed at the gene-level. If the goal is to examine transcript-level expression, HTSeq and featureCounts are not suitable for the purpose. They are developed explicitly for gene-level read counts. When they count the reads mapped to transcripts rather than genes, reads mapped to exons shared by several transcripts will then be considered ambiguous and discarded by default. Kallisto and Salmon are able to produce both transcript- and gene-level expression output. In addition, since the simulated data is generated using RSEM, we cannot fairly compare RSEM in the simulated data experiments.

One limitation of this study is that the simulated datasets are based on polyA-selected RNA-Seq. It would be helpful to evaluate RNA-Seq methods that capture more lncRNAs. However, this study focuses on using existing RNA-seq datasets for profiling lncRNAs in cancer. More importantly, in cancer research most of the available datasets, including TCGA, were generated using polyA-selected RNA-Seq. Thus, our study still provides valuable guidelines for researchers studying lncRNAs in cancer.

The discrepancy between alignment and pseudoalignment methods in our work is striking and we suspect that this is due to pseudoalignment methods' focus on the difficulty for accurate quantification for reads that cannot be uniquely aligned. An RNA-seq experiment can be regarded as the statistical problem of random sampling of subsequences (i.e., reads) from spliced transcripts of different length. Several of these transcripts may share the same exact exons bringing uncertainty to the reads drawn from those shared exons. Thus, it is important to properly model this statistical problem and to capture and resolve such uncertainty as accurately as possible. We speculate that pseudoalignment methods are able to model this problem more accurately by iteratively assigning reads to a transcript or a set of transcripts with a certain probability. Furthermore, the speed improvement achieved by pseudoalignment rather

than alignment-based methods (Additional file 17) allows using more robust statistical inference techniques such as bootstrapping.

Potential implications

In summary, considering the consistency with the ground truth, flexibility at both gene- and transcript-level analysis, and the computational resources, pseudoalignment methods Kallisto and Salmon are recommended for RNA-Seq analysis for lncRNAs, with Kallisto performing slightly better than Salmon. The full transcriptome annotation including protein coding genes, lncRNAs, and others, is also the recommended strategy for RNA-Seq analysis.

The large amount of data produced by the next-generation sequencing techniques has posed great challenges for fast and scalable data analysis. Our study implicates that for RNA-Seq datasets, incorporating pseudoalignment methods into the analytical framework can achieve high accuracy with minimum computing requirements. Moreover, with more and more RNA-Seq datasets specifically for studying lncRNAs becoming available, our work also lays the basis for a more comprehensive evaluation of tools for lncRNA expression quantification.

Methods

Definitions

Here we clarify and define relevant terms. i) Genes and transcripts: a transcript, sometimes also referred to as an isoform, is composed by exons. An exon is any part of a gene that will encode a part of the final mature RNA. A gene is a collection of transcripts. Transcripts of the same gene often share exons. In this study the analysis was performed at the gene-level. ii) Expressed genes: when describing a single sample, "expressed genes" refer to genes with FPKM (Fragments Per Kilobase per Million mapped reads) value equal or above one in that particular sample. When describing multiple samples in a dataset, "expressed genes" refer to the genes with median FPKM value equal or above one across the cohort of samples. iii) Discordant genes: these are defined as genes whose Spearman's correlation of FPKM values with the ground truth is less than 0.7, when compared across a cohort of samples.

Reference genome and transcriptome

Human transcriptome version GENCODE release 27 (GTF file and transcriptome fasta file) were downloaded from the GENCODE FTP site. The GENCODE release 27 collects 58,288 genes and 200,401 transcripts, among them there are 19,836 protein-coding genes (Additional file 1). If defining lncRNA as non-coding, 3-prime overlapping ncRNA, antisense, bidirectional promoter lncRNA, long intergenic noncoding RNAs (lincRNA), macro lncRNA, sense intronic, and sense overlapping, there are 14,168 lncRNAs. The primary assembly of human genome GRCh38 was also downloaded from the same site.

The NONCODE database [2] collecting 172,216 transcripts from 96,308 lncRNA genes (version 5) were downloaded from their website, and merged with GENCODE to create a new set of transcriptome annotation. Both the GENCODE version and GENCODE combined with NONCODE version were used to analyze the datasets and simulate two sets of ground truth for comparison.

The indexes for Kallisto and Salmon were built using the transcriptome fasta file. The indexes for RSEM and STAR were built using transcriptome GTF file and GRCh38 genome sequences. The indexes for Subread and HISAT2 were built using GRCh38 genome sequences.

Gene features (number of transcripts, number of exons, transcript length) were generated from GTF file using in-house script. Unique kmers of genes were generated using script from Computational Genomics Analysis and Training (CGAT).

Pipelines

Eight pipelines were applied to process the datasets, including two pseudoalignment methods Kallisto and Salmon, and a combination of read aligners (STAR, Subread, HISAT2) and quantification tools (HTSeq and featureCounts).

Kallisto, version 0.44.0, quant mode. Default parameters were applied. Stand-specific option was set as '–rf-stranded' for reverse-stranded samples.

Salmon, version 0.9.1, quant mode. Default parameters were applied. Stand-specific option: ' -l A'. Kallisto and Salmon measure the expression level of each transcript by default. To get gene-level expression results, the package tximport [35] was used.

STAR, version 2.5.4a. Two-pass mode were used for mapping.

Subread, version 1.6.1. Stand-specific option was set as ' -S ff ' for un-stranded samples and ' -S rf ' for reverse-stranded samples. Othersettings: ' –multiMapping -B 4 -t 0 '.

HISAT2, version 2.1.0. Stand-specific option was set as '–rna-strandness RF' for reverse-stranded samples.

HTSeq, version 0.7.2. Mapped reads from STAR, Subread and HISAT2 were counted for each gene according to the GTF file from either GENCODE or GENCODE+NONCODE annotations. Stand-specific option was set as '-s no' for un-stranded samples and '-s yes' for reverse-stranded samples.

featureCounts, version 1.6.1. Mapped reads from STAR, Subread and HISAT2 were counted for each gene according to the GTF file from either GENCODE or GENCODE+NONCODE annotations. Stand-specific option was set as '-s 2' for reverse-stranded samples. HTSeq and featureCounts output read count for each gene. FPKM values were generated from read counts with in-house scripts.

To compare the computational resources required by each tool, RNA-Seq reads from five original samples were chosen and processed with each tool, with the number of threads set at four.

Clustering and heat maps

Hierarchical clustering was performed for the sample-method matrix. Euclidean distance and average linkage were used for both the columns and rows. The clustering dendrogram was cut into four groups and the number of times that every two methods are clustered together were counted and used to construct a similarity matrix.

Statistical tests

Mann-Whitney U test was used to test the difference between two continuous variables, and chi-squared test was used to test the difference of two ratios. Unless otherwise specified, all the tests have p values less than 0.001, thus, they are not explicitly explained in the main text.

PANCAN RNA-Seq analysis

The raw RNA-Seq sequencing data of TCGA samples was downloaded from ISB-CGC and processed with Kallisto, using GEN-

CODE (version 27) as transcriptome reference.

Availability of source code and requirements

- Project name: RNASeq_pipeline
- Project home page: github.com/gevaertlab/RNASeq_pipeline
- Operating system(s): Platform independent
- Programming language: Linux

Availability of supporting data and materials

The TCGA RNA-Seq re-analysis results and the simulated datasets are available in the Stanford Medicine Box: stanfordmedicine.app.box.com/s/lu703xuaulfz02vgd2lunxnvt4mfvo3q

The web interface for investigating and visualizing individual gene expression can be found in http://apps.gevaertlab.stanford.edu.

Declarations

List of abbreviations

FPKM: Fragments per kilobase million
HOTAIR: Hox transcript antisense RNA
lncRNA: long non-coding RNA
RNA-Seq: RNA sequencing
TCGA: The Cancer Genome Atlas
TERC: Telomerase RNA component
TERT: Telomerase reverse transcriptase

Competing Interests

The authors declare that they have no competing interests.

Funding

This work was supported by National Institute of Dental & Craniofacial Research (NIDCR) (U01 DE025188), the National Institute of Biomedical Imaging and Bioengineering (R01 EB020527), and the National Cancer Institute (U01 CA217851), all of the National Institutes of Health. The content is solely the responsibility of the authors and does not necessarily represent the official views of the National Institutes of Health. The funders did not play a role in the design and execution of this study.

Author's Contributions

H.Z. and O.G. conceived and designed the study. H.Z., K.B. and M.H performed data analysis. H.Z. wrote the manuscript and all authors participated in preparing the manuscript.

References

1. Mattick JS, Rinn JL. Discovery and annotation of long noncoding RNAs. Nature structural & molecular biology 2015;22(1):5.
2. Fang S, Zhang L, Guo J, Niu Y, Wu Y, Li H, et al. NONCODEV5: a comprehensive annotation database for long non-coding RNAs. Nucleic Acids Res 2018 Jan;46(D1):D308–d314.
3. Iyer MK, Niknafs YS, Malik R, Singhal U, Sahu A, Hosono Y, et al. The landscape of long noncoding RNAs in the human transcriptome. Nature genetics 2015;47(3):199.
4. Derrien T, Johnson R, Bussotti G, Tanzer A, Djebali S, Tilgner H, et al. The GENCODE v7 catalog of human long noncoding RNAs: analysis of their gene structure, evolution, and expression. Genome Res 2012 Sep;22(9):1775–89.
5. Fatica A, Bozzoni I. Long non-coding RNAs: new players in cell differentiation and development. Nature Reviews Genetics 2014;15(1):7.
6. Esteller M. Non-coding RNAs in human disease. Nature reviews Genetics 2011;12(12):861.
7. Wang KC, Chang HY. Molecular mechanisms of long noncoding RNAs. Mol Cell 2011 Sep;43(6):904–14.
8. Schmitt AM, Chang HY. Long Noncoding RNAs in Cancer Pathways. Cancer Cell 2016 Apr;29(4):452–463.
9. Huarte M. The emerging role of lncRNAs in cancer. Nat Med 2015 Nov;21(11):1253–61.
10. Gupta RA, Shah N, Wang KC, Kim J, Horlings HM, Wong DJ, et al. Long non-coding RNA HOTAIR reprograms chromatin state to promote cancer metastasis. Nature 2010 Apr;464(7291):1071–6.
11. Zhang J, Zhang P, Wang L, Piao HL, Ma L. Long noncoding RNA HOTAIR in carcinogenesis and metastasis. Acta Biochim Biophys Sin (Shanghai) 2014 Jan;46(1):1–5.
12. Yu W, Gius D, Onyango P, Muldoon-Jacobs K, Karp J, Feinberg AP, et al. Epigenetic silencing of tumour suppressor gene p15 by its antisense RNA. Nature 2008 Jan;451(7175):202–6.
13. Li XX, Liang XJ, Zhou LY, Liu RJ, Bi W, Zhang S, et al. Analysis of Differential Expressions of Long Non-coding RNAs in Nasopharyngeal Carcinoma Using Next-generation Deep Sequencing. J Cancer 2018;9(11):1943–1950.
14. Tian Z, Wen S, Zhang Y, Shi X, Zhu Y, Xu Y, et al. Identification of dysregulated long non-coding RNAs/microRNAs/mRNAs in TNM I stage lung adenocarcinoma. Oncotarget 2017 Aug;8(31):51703–51718.
15. Yang W, Li Y, Song X, Xu J, Xie J. Genome-wide analysis of long noncoding RNA and mRNA co-expression profile in intrahepatic cholangiocarcinoma tissue by RNA sequencing. Oncotarget 2017 Apr;8(16):26591–26599.
16. Su X, Malouf GG, Chen Y, Zhang J, Yao H, Valero V, et al. Comprehensive analysis of long non-coding RNAs in human breast cancer clinical subtypes. Oncotarget 2014;5(20):9864.
17. Yan X, Hu Z, Feng Y, Hu X, Yuan J, Zhao SD, et al. Comprehensive genomic characterization of long non-coding RNAs across human cancers. Cancer Cell 2015;28(4):529–540.
18. Zhao S, Zhang Y, Gordon W, Quan J, Xi H, Du S, et al. Comparison of stranded and non-stranded RNA-seq transcriptome profiling and investigation of gene overlap. BMC Genomics 2015 Sep;16:675.
19. Sigurgeirsson B, Emanuelsson O, Lundeberg J. Analysis of stranded information using an automated procedure for strand specific RNA sequencing. BMC Genomics 2014 Jul;15:631.
20. Everaert C, Luypaert M, Maag JLV, Cheng QX, Dinger ME, Hellemans J, et al. Benchmarking of RNA-sequencing analysis workflows using whole-transcriptome RT-qPCR expression data. Sci Rep 2017 May;7(1):1559.
21. Teng M, Love MI, Davis CA, Djebali S, Dobin A, Graveley BR, et al. A benchmark for RNA-seq quantification pipelines. Genome Biol 2016 Apr;17:74.
22. Bray NL, Pimentel H, Melsted P, Pachter L. Near-optimal probabilistic RNA-seq quantification. Nat Biotechnol 2016 May;34(5):525–7.
23. Patro R, Duggal G, Love MI, Irizarry RA, Kingsford C.

Salmon provides fast and bias-aware quantification of transcript expression. Nat Methods 2017 Apr;14(4):417–419.
24. Anders S, Pyl PT, Huber W. HTSeq–a Python framework to work with high-throughput sequencing data. Bioinformatics 2015 Jan;31(2):166–9.
25. Liao Y, Smyth GK, Shi W. featureCounts: an efficient general purpose program for assigning sequence reads to genomic features. Bioinformatics 2014 Apr;30(7):923–30.
26. Dobin A, Davis CA, Schlesinger F, Drenkow J, Zaleski C, Jha S, et al. STAR: ultrafast universal RNA-seq aligner. Bioinformatics 2013 Jan;29(1):15–21.
27. Liao Y, Smyth GK, Shi W. The Subread aligner: fast, accurate and scalable read mapping by seed-and-vote. Nucleic Acids Res 2013 May;41(10):e108.
28. Kim D, Langmead B, Salzberg SL. HISAT: a fast spliced aligner with low memory requirements. Nat Methods 2015 Apr;12(4):357–60.
29. Krueger F. Trim Galore. A wrapper tool around Cutadapt and FastQC to consistently apply quality and adapter trimming to FastQ files 2015;.
30. Li B, Dewey CN. RSEM: accurate transcript quantification from RNA-Seq data with or without a reference genome. BMC Bioinformatics 2011;12:323.
31. Maag JL, Fisher OM, Levert-Mignon A, Kaczorowski DC, Thomas ML, Hussey DJ, et al. Novel Aberrations Uncovered in Barrett's Esophagus and Esophageal Adenocarcinoma Using Whole Transcriptome Sequencing. Molecular Cancer Research 2017;15(11):1558–1569.
32. Zeng JH, Liang L, He RQ, Tang RX, Cai XY, Chen JQ, et al. Comprehensive investigation of a novel differentially expressed lncRNA expression profile signature to assess the survival of patients with colorectal adenocarcinoma. Oncotarget 2017 Mar;8(10):16811–16828.
33. Zhang C, Zhang B, Lin LL, Zhao S. Evaluation and comparison of computational tools for RNA-seq isoform quantification. BMC Genomics 2017 Aug;18(1):583.
34. Jin H, Wan YW, Liu Z. Comprehensive evaluation of RNA-seq quantification methods for linearity. BMC bioinformatics 2017;18(4):117.
35. Soneson C, Love MI, Robinson MD. Differential analyses for RNA-seq: transcript-level estimates improve gene-level inferences. F1000Research 2015;4.

Figures

Figure 1. The effect of incomplete transcriptome annotation on the expression quantification of lncRNAs.

A) Boxplot of the percentages of expressed lncRNAs (FPKM>=1) detected with each tool, using full annotation or lncRNA-only annotation. The ground truth expression used for data simulation was also plotted for comparison. Each point in the boxplot represents one sample. For each library type, ten samples were included in the analysis. B) The expression profile of lncRNAs in one representative sample from each of the datasets was shown with violin plot (left) and scatter plot (right), which demonstrates the over-estimation effect using lncRNA-only annotation compared with full annotation, in all the three samples for both pseudoalignment and alignment-based methods. PCT, percentage; FPKM, fragments per kilobase million.

A) Boxplot of the percentage of expressed lncRNAs detected

Figure 2. Pseudoalignment methods outperform alignment-based methods.

with each tool. Each point in the boxplot represents one sample. B) Sample-level and C) gene-level comparison of each tool with the ground truth. The calculation of Spearman's correlation and Euclidean distance, and linear regression was performed using log-transformed FPKM values reported by each tool compared with the ground truth. In sample-level comparison, only expressed lncRNAs in each sample were included in the analysis. Each point in the boxplot represents one sample. In gene-level comparison, lncRNAs with median FPKM value above one in the corresponding dataset were included in the analysis. Each point in the boxplot represents one gene. Spearman, Spearman's rank-order correlation; PCT, percentage; MSE, mean squared error; AdjR$\hat{2}$, adjusted R squared.

Figure 3. Similarity matrix of different methods.

Each grid in the matrix is the number of times that two methods were clustered in the same group, which is counted from hierarchical clustering of 1,075 expressed lncRNAs in any of the three datasets. The group number for cutting the hierarchical clustering dendrogram was set as four. Euclidean distance and average linkage were used for single-gene level clustering.

Figure 4. Features of total, expressed and discordant lncRNAs.

The proportion of A) The lncRNA type and B) the number of transcripts of lncRNAs in GENCODE and expressed lncRNAs in samples from the three datasets. Each point in the boxplot represents one sample. C and D) The lncRNA types, number of transcripts, and sequence uniqueness of expressed lncRNAs (median FPKM above one in the dataset). Spearman's correlation was calculated comparing each method and ground truth. Each point in the boxplot represents one gene. Numbers in brackets in x-axis labels are the number of genes in certain category.

Figure 5. Examples of lncRNAs in cancer.

The lncRNAs that were previously reported to play a role in cancer are shown in the three datasets. The heatmaps show the FPKM value (log transformed) obtained from each method and the ground truth. The Euclidean distance and average linkage were used for clustering.

Additional Files

Additional file 1 — The genes and transcripts in GENCODE release 27.

Additional file 2 — The percentage of expressed genes using each method and different gene annotation sets.

Additional file 3 — The effect of incomplete transcriptome annotation on the expression quantification of protein-coding genes.

Additional file 4 — The percentage of expressed lncRNA genes using each method and full annotation.

Additional file 5 — Examples of sample-level comparison of each method and the ground truth.

Additional file 6 — Sample-level comparison of gene expression.

Additional file 7 — Statistical tests for gene-level comparison between pseudoalignment methods and alignment-based methods.

Additional file 8 — Gene-level comparison of gene expression.

Additional file 9 — Features of total and expressed lncRNAs.

Additional file 10 — Feature (lncRNA type) of discordant lncRNAs.

Additional file 11 — Features of discordant lncRNAs.

Additional file 12 — Feature (number of transcripts) of discordant lncRNAs.

Additional file 13 — Feature (transcript length) of discordant lncRNAs.

Additional file 14 — Feature (number of exons) of discordant lncRNAs.

Additional file 15 — Feature (sequence uniqueness) of discordant lncRNAs.

Additional file 16 — Overall feature breakdown of GENCODE, expressed, and discordant lncRNAs.

Additional file 17 — Computational resources required for each method.

Figure 1

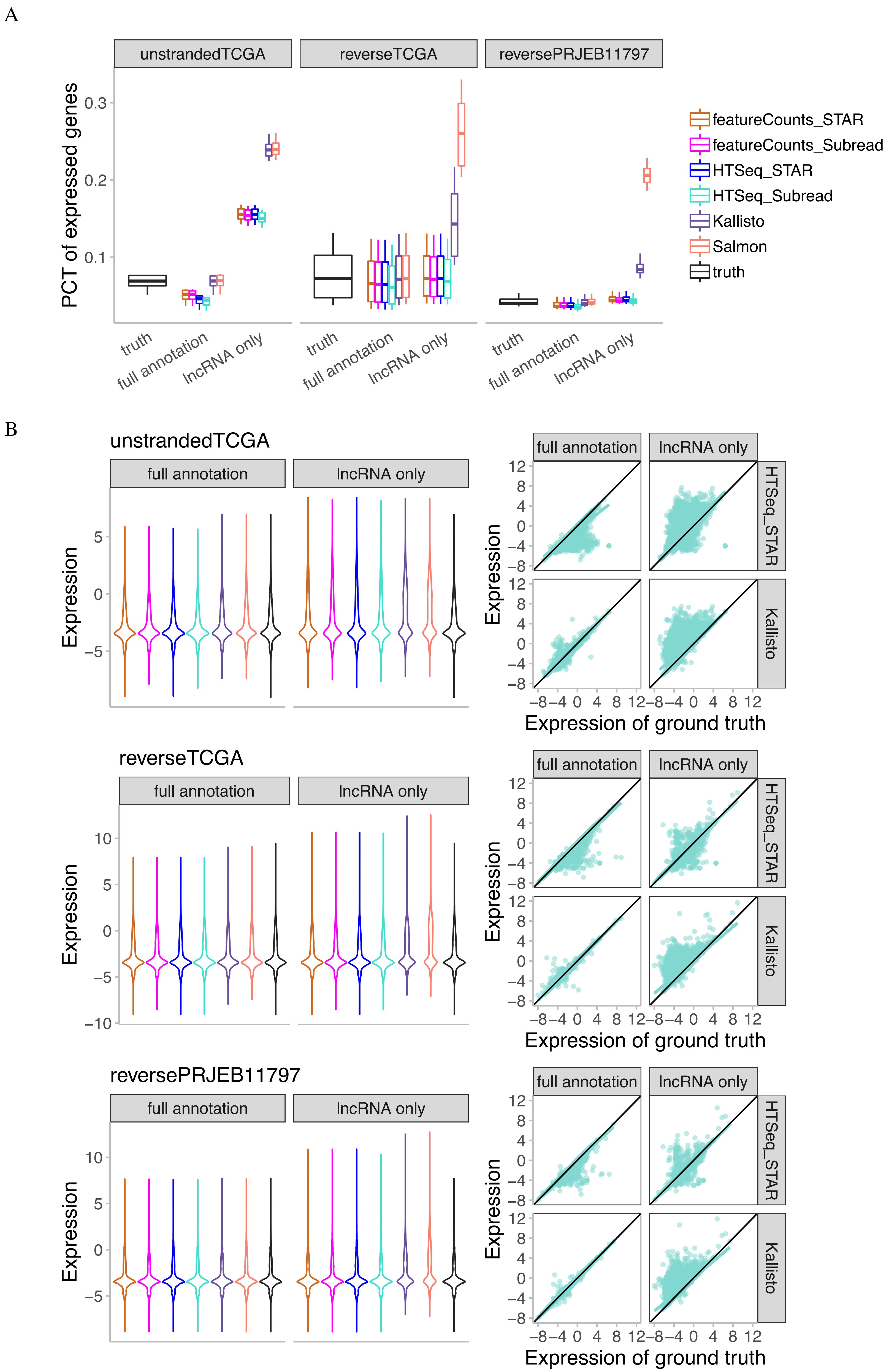
A
unstrandedTCGA
reverseTCGA
reversePRJEB11797
PCT of expressed genes
0.3
0.2
0.1
truth
full annotation
lncRNA only
featureCounts_STAR
featureCounts_Subread
HTSeq_STAR
HTSeq_Subread
Kallisto
Salmon
truth
B
unstrandedTCGA
full annotation
lncRNA only
Expression
HTSeq_STAR
Kallisto
Expression of ground truth
reverseTCGA
reversePRJEB11797

Figure 2

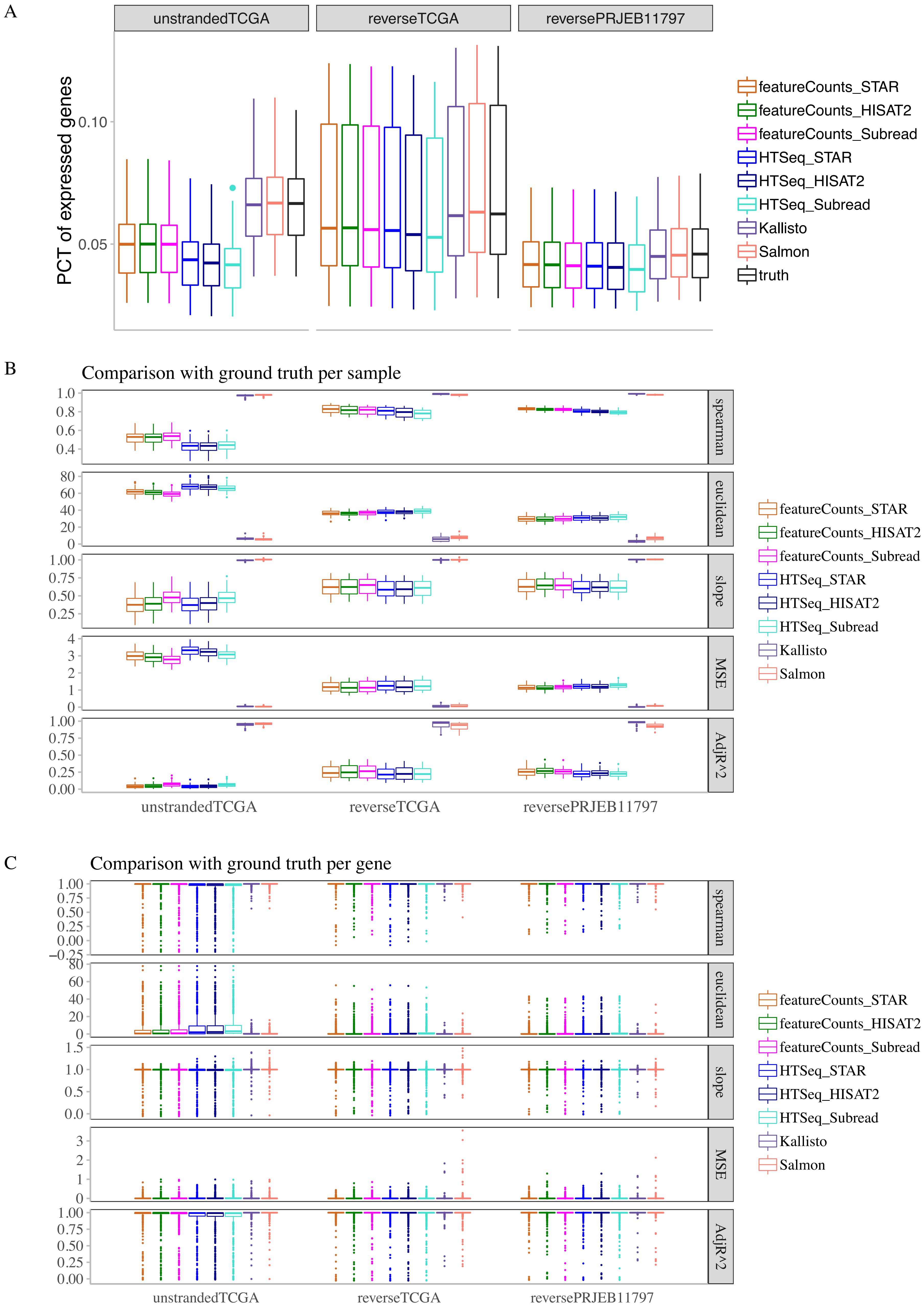
A
unstrandedTCGA
reverseTCGA
reversePRJEB11797
PCT of expressed genes
0.10
0.05
featureCounts_STAR
featureCounts_HISAT2
featureCounts_Subread
HTSeq_STAR
HTSeq_HISAT2
HTSeq_Subread
Kallisto
Salmon
truth
B
Comparison with ground truth per sample
spearman
euclidean
slope
MSE
AdjR^2
unstrandedTCGA
reverseTCGA
reversePRJEB11797
C
Comparison with ground truth per gene
spearman
euclidean
slope
MSE
AdjR^2
unstrandedTCGA
reverseTCGA
reversePRJEB11797

Figure 3

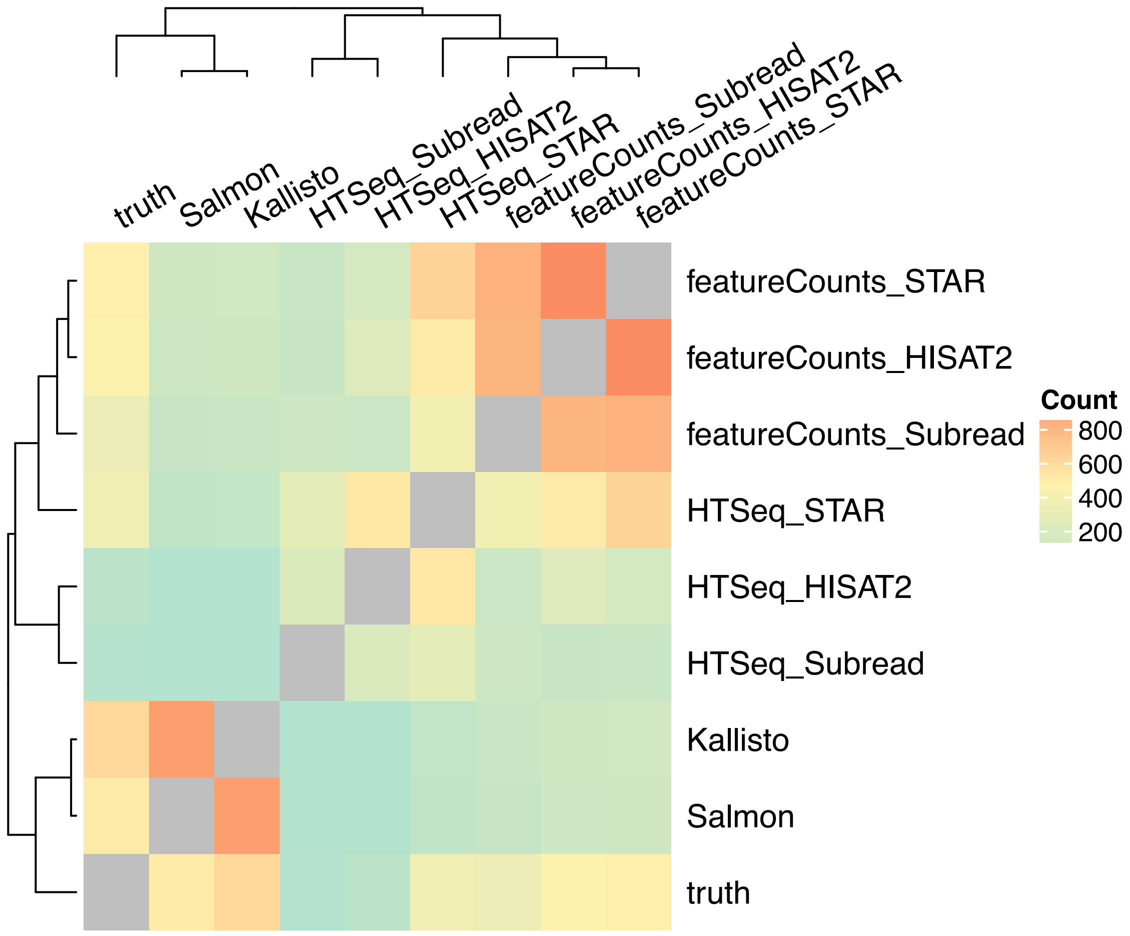
truth
Salmon
Kallisto
HTSeq_Subread
HTSeq_HISAT2
HTSeq_STAR
featureCounts_Subread
featureCounts_HISAT2
featureCounts_STAR
featureCounts_STAR
featureCounts_HISAT2
featureCounts_Subread
HTSeq_STAR
HTSeq_HISAT2
HTSeq_Subread
Kallisto
Salmon
truth
Count
800
600
400
200

Figure 4

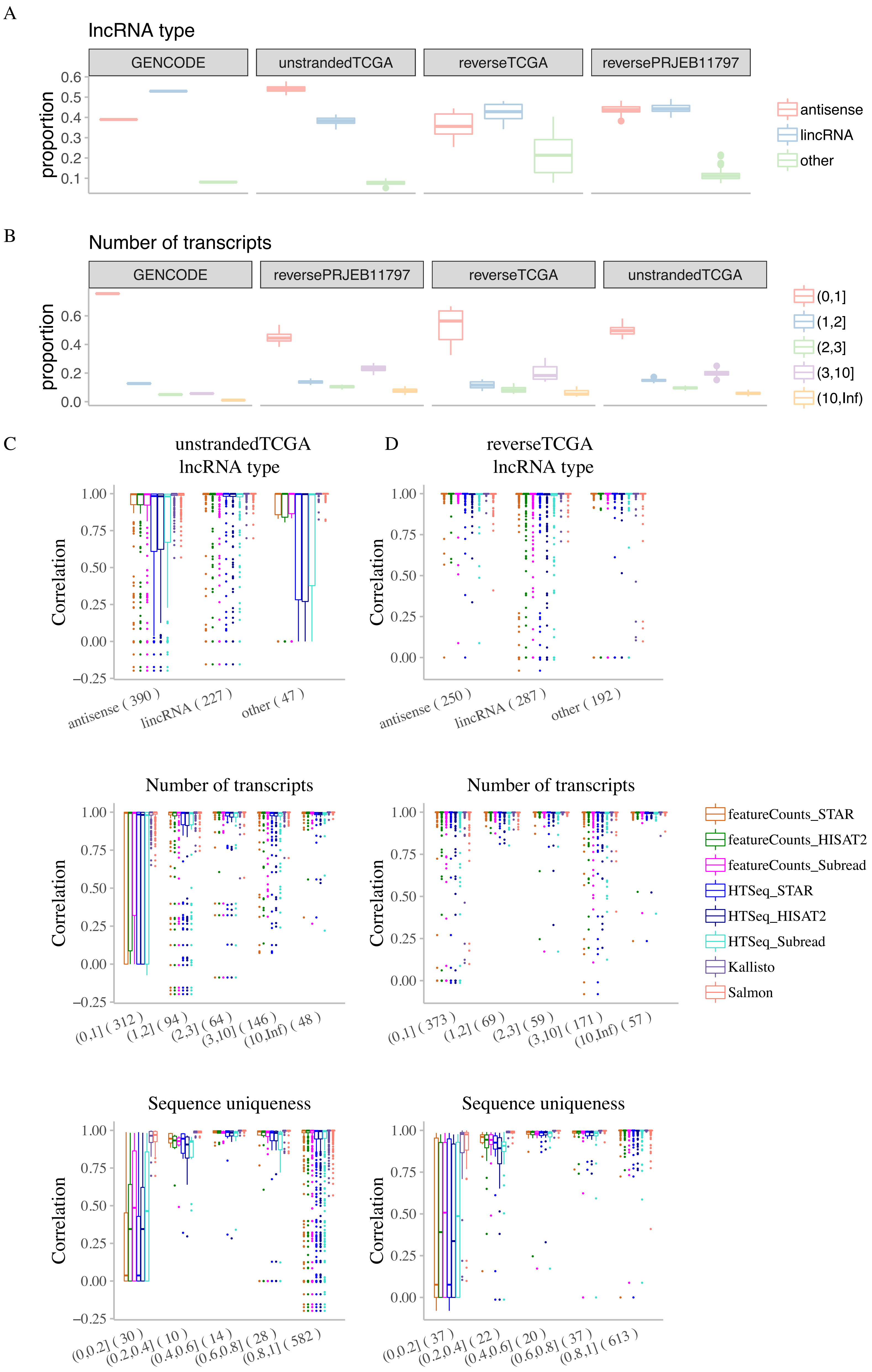
A
lncRNA type
GENCODE
unstrandedTCGA
reverseTCGA
reversePRJEB11797
proportion
antisense
lincRNA
other
B
Number of transcripts
GENCODE
reversePRJEB11797
reverseTCGA
unstrandedTCGA
proportion
(0,1]
(1,2]
(2,3]
(3,10]
(10,Inf)
C
unstrandedTCGA
lncRNA type
Correlation
antisense (390)
lincRNA (227)
other (47)
D
reverseTCGA
lncRNA type
antisense (250)
lincRNA (287)
other (192)
Number of transcripts
(0,1] (312)
(1,2] (94)
(2,3] (64)
(3,10] (146)
(10,Inf) (48)
(0,1] (373)
(1,2] (69)
(2,3] (59)
(3,10] (171)
(10,Inf) (57)
featureCounts_STAR
featureCounts_HISAT2
featureCounts_Subread
HTSeq_STAR
HTSeq_HISAT2
HTSeq_Subread
Kallisto
Salmon
Sequence uniqueness
(0,0.2] (30)
(0.2,0.4] (10)
(0.4,0.6] (14)
(0.6,0.8] (28)
(0.8,1] (582)
(0,0.2] (37)
(0.2,0.4] (22)
(0.4,0.6] (20)
(0.6,0.8] (37)
(0.8,1] (613)

Figure 5

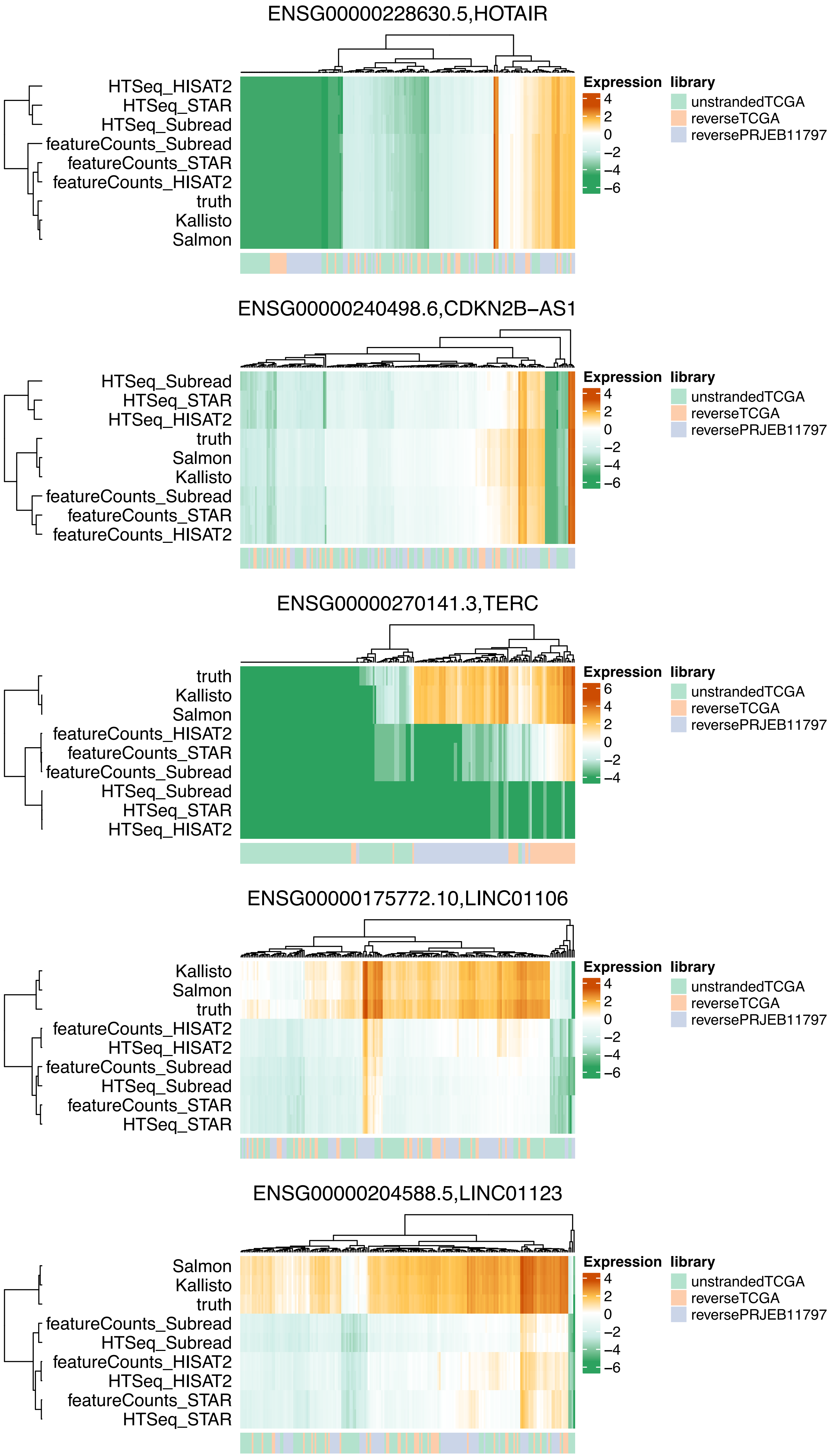
ENSG00000228630.5,HOTAIR
HTSeq_HISAT2
HTSeq_STAR
HTSeq_Subread
featureCounts_Subread
featureCounts_STAR
featureCounts_HISAT2
truth
Kallisto
Salmon
Expression
4
2
0
−2
−4
−6
library
unstrandedTCGA
reverseTCGA
reversePRJEB11797
ENSG00000240498.6,CDKN2B−AS1
HTSeq_Subread
HTSeq_STAR
HTSeq_HISAT2
truth
Salmon
Kallisto
featureCounts_Subread
featureCounts_STAR
featureCounts_HISAT2
Expression
4
2
0
−2
−4
−6
library
unstrandedTCGA
reverseTCGA
reversePRJEB11797
ENSG00000270141.3,TERC
truth
Kallisto
Salmon
featureCounts_HISAT2
featureCounts_STAR
featureCounts_Subread
HTSeq_Subread
HTSeq_STAR
HTSeq_HISAT2
Expression
6
4
2
0
−2
−4
library
unstrandedTCGA
reverseTCGA
reversePRJEB11797
ENSG00000175772.10,LINC01106
Kallisto
Salmon
truth
featureCounts_HISAT2
HTSeq_HISAT2
featureCounts_Subread
HTSeq_Subread
featureCounts_STAR
HTSeq_STAR
Expression
4
2
0
−2
−4
−6
library
unstrandedTCGA
reverseTCGA
reversePRJEB11797
ENSG00000204588.5,LINC01123
Salmon
Kallisto
truth
featureCounts_Subread
HTSeq_Subread
featureCounts_HISAT2
HTSeq_HISAT2
featureCounts_STAR
HTSeq_STAR
Expression
4
2
0
−2
−4
−6
library
unstrandedTCGA
reverseTCGA
reversePRJEB11797

Manuscript

Click here to access/download
Supplementary Material
Giga.supplementary.pdf

Manuscript Click here to download Personal Cover cover_letter_Giga.pdf

April 8, 2019

Dear Editor,

We would like to submit the manuscript "**Benchmark of lncRNA Quantification for RNA-Seq of Cancer Samples**" for consideration to publish in GigaScience.

Long non-coding RNAs (lncRNAs) are important regulators of many biological processes. While many studies have exploited public data such as The Cancer Genome Atlas (TCGA) to study lncRNAs in cancer, there is no accepted gold standard pipeline yet for RNA-Seq analysis of lncRNAs. Thus, it is crucial to evaluate the popular RNA-Seq tools for the purpose.

In this study, we evaluated several key aspects in RNA-Seq analysis:

a) **Tools for quantification**, which are broadly classified into pseudoalignment methods (Kallisto and Salmon) and alignment-based methods (HTSeq and featureCounts in combination with read aligners, STAR, Subread, and HISAT2);
b) **RNA-Seq protocols for library preparation**. We evaluated the tools on both un-stranded data, which is the case for most TCGA samples, and strand-specific data;
c) **Transcriptome annotations**. We examined the lncRNAs in commonly used GENCODE annotation (≥14k lncRNAs) and the NONCODE database(≥ 90k lncRNAs).

Most importantly, we found that pseudoalignment methods outperform alignment-based methods for lncRNA quantification in both sample- and gene-level comparison, regardless of the RNA-Seq protocols, choice of aligners and transcriptome annotation. Pseudoalignment methods detect more lncRNAs than alignment-based methods and correlate highly with simulated ground truth. On the contrary, alignment-based methods often underestimate lncRNA expression. We also found that Kallisto performs slightly better than Salmon, which is particularly interesting, especially in the current heated debate between the developers of both Kallisto and Salmon.

Moreover, among the different types of lncRNAs, antisense lncRNAs are quantified less accurately than lincRNAs in un-stranded samples using alignment-based methods. This can be improved by either adopting a reverse-stranded protocol for RNA-Seq or using pseudoalignment methods for analysis, or a combination of both. If only un-stranded samples are available, as is the case with most TCGA samples, it then becomes vital to choose the right method like Kallisto or Salmon for analysis, since these two methods are seldom affected by the type of lncRNAs nor

the type of RNA-Seq protocols and have very few number of discordant lncRNAs, even for antisense lncRNAs, or lncRNAs with low sequence uniqueness.

Lastly, we observed that incomplete annotation strongly overestimates expression of both lncRNAs and protein-coding genes. Full transcriptome annotation, including both protein coding and noncoding RNAs, greatly improves the specificity of lncRNA expression quantification, a fact that has not been shown before, and is not widely known.

The study has attracted great interest from the research community. After the release of the first version as a pre-print on bioRxiv (biorxiv.org/content/early/2018/01/02/241869), over 60 researchers have shared it on twitter and it was in the top 5% of all research outputs measured by bioRxiv altmetric (biorxiv.altmetric.com/details/31185610). **We haven't updated the bioRxiv version yet**, and we believe the additional results in this manuscript will attract more attention.

It is worth noting that many studies still adopt alignment-based methods for RNA-Seq, which behave worse than the pseudoalignment methods, as shown by our study. For example, **HTSeq**, one of the alignment-based methods, is used by the **default workflow for TCGA data in Genomic Data Commons data portal**. Since more and more researchers are using these datasets to investigate lncRNA expression, this study would provide timely recommendations for the best practice of RNA-Seq analysis for lncRNAs. We have reprocessed the TCGA RNA-Seq data using Kallisto with GENCDOE annotation. The results are deposited online (apps.gevaertlab.stanford.edu/) for researchers studying lncRNAs in TCGA data.

In summary, this work **provides timely and useful recommendations for the research community who are studying lncRNAs**, especially for those who are exploring public resources such as TCGA RNA-Seq data.

We hope this manuscript will receive favorable review from the journal and look forward to hearing from you.

Yours Sincerely,

Olivier Gevaert
Assistant Professor of Medicine (Biomedical Informatics Research) and Biomedical Data Science,

Stanford Center for Biomedical Informatics Research (BMIR)
Department of Medicine, and Department of Biomedical Data Science
Stanford University
1265 Welch Rd, Stanford, CA 94305-5479
Office: 650-721-2378
Mobile: 650-336-8418
gevaertlab.stanford.edu
