# Supplementary material for: Benchmark of long non-coding RNA quantification for RNA sequencing of cancer samples
Source: Gigascience. 2019 Dec 6;8(12):giz145. doi: 10.1093/gigascience/giz145 (PMC6897288; doi:10.1093/gigascience/giz145)
Supplement: giz145_Supplemental_File [file giz145_supplemental_file.pdf]

Additional Files

Additional file 1 — The genes and transcripts in GENCODE release 27.

Additional file 2 — The percentage of expressed genes using each method and different gene annotation sets.

Additional file 3 — The effect of incomplete transcriptome annotation on the expression quantification of protein- coding genes.

Additional file 4 — The percentage of expressed lncRNA genes using each method and full annotation.

Additional file 5 — Examples of sample-level comparison of each method and the ground truth.
Additional file 6 — Sample-level comparison of gene expression.

Additional file 7 — Statistical tests for gene-level comparison between pseudoalignment methods and alignment- based methods.

Additional file 8 — Gene-level comparison of gene expression.

Additional file 9 — Features of total and expressed lncRNAs.

Additional file 10 — Feature (lncRNA type) of discordant lncRNAs. Additional file 11 — Features of discordant lncRNAs.

Additional file 12 — Feature (number of transcripts) of discordant lncRNAs.

Additional file 13 — Feature (transcript length) of discordant lncRNAs.

Additional file 14 — Feature (number of exons) of discordant lncRNAs.

Additional file 15 — Feature (sequence uniqueness) of discordant lncRNAs.

Additional file 16 — Overall feature breakdown of GENCODE, expressed, and discordant lncRNAs.

Additional file 17 — Reads mapping of discordant lncRNAs.

Additional file 1. The genes and transcripts in GENCODE release 27

	genes	transcripts	Median length of transcripts	Median number of exons	Median number of transcripts per gene
**Total**	58,288	200,401	803	2	1
**Protein-coding**	19,836	80,930	1562	5.5	6
**lncRNA**	14,168	25,779	611	2	1
**other**	24,284	93,692	376	1	1

Additional file 2. The percentage of expressed genes using each method and different gene annotation sets

method	library type	lncRNA				protein-coding			
		annotation	mean	median	sd	annotation	mean	median	sd
featureCounts_STAR		all	5.04%	5.21%	0.82%	all	60.53%	60.31%	2.36%
featureCounts_STAR		lncRNA	15.58%	15.56%	0.84%	proteincoding	61.06%	60.83%	2.34%
featureCounts_Subread		all	5.01%	5.21%	0.82%	all	60.61%	60.44%	2.34%
featureCounts_Subread		lncRNA	15.42%	15.39%	0.82%	proteincoding	61.15%	60.95%	2.31%
HTSeq_STAR		all	4.40%	4.61%	0.75%	all	59.81%	59.51%	2.33%
HTSeq_STAR		lncRNA	15.52%	15.51%	0.83%	proteincoding	60.55%	60.27%	2.31%
HTSeq_Subread	unstrandedTCGA	all	4.16%	4.36%	0.69%	all	59.54%	59.21%	2.30%
HTSeq_Subread		lncRNA	15.09%	15.05%	0.77%	proteincoding	60.31%	59.99%	2.28%
Kallisto		all	6.76%	6.93%	0.98%	all	62.53%	62.27%	2.41%
Kallisto		lncRNA	24.05%	23.88%	1.22%	proteincoding	63.00%	62.75%	2.38%
Salmon		all	6.81%	6.98%	1.00%	all	62.54%	62.28%	2.41%
Salmon		lncRNA	24.18%	23.98%	1.20%	proteincoding	63.01%	62.76%	2.38%
truth		truth	6.76%	6.92%	0.99%	truth	62.58%	62.32%	2.43%
featureCounts_STAR		all	7.10%	6.57%	3.28%	all	61.61%	61.44%	1.15%
featureCounts_STAR		lncRNA	7.79%	7.26%	3.27%	proteincoding	61.84%	61.69%	1.17%
featureCounts_Subread		all	7.02%	6.47%	3.24%	all	61.64%	61.47%	1.17%
featureCounts_Subread		lncRNA	7.70%	7.15%	3.25%	proteincoding	61.85%	61.68%	1.18%
HTSeq_STAR		all	6.99%	6.45%	3.27%	all	61.30%	61.14%	1.17%
HTSeq_STAR		lncRNA	7.78%	7.24%	3.28%	proteincoding	61.55%	61.39%	1.19%
HTSeq_Subread	reverseTCGA	all	6.66%	6.10%	3.08%	all	61.13%	61.00%	1.15%
HTSeq_Subread		lncRNA	7.42%	6.86%	3.10%	proteincoding	61.38%	61.24%	1.16%
Kallisto		all	7.67%	7.16%	3.38%	all	63.41%	63.26%	1.07%
Kallisto		lncRNA	14.62%	14.30%	4.85%	proteincoding	63.81%	63.69%	1.09%
Salmon		all	7.76%	7.26%	3.39%	all	63.42%	63.28%	1.07%
Salmon		lncRNA	26.17%	26.05%	4.89%	proteincoding	64.07%	63.98%	1.15%
truth		truth	7.72%	7.23%	3.40%	truth	63.39%	63.19%	1.12%
featureCounts_STAR		all	3.83%	3.66%	0.49%	all	62.42%	62.21%	1.06%
featureCounts_STAR		lncRNA	4.55%	4.40%	0.49%	proteincoding	62.65%	62.44%	1.08%
featureCounts_Subread		all	3.79%	3.61%	0.47%	all	62.52%	62.29%	1.05%
featureCounts_Subread		lncRNA	4.48%	4.33%	0.48%	proteincoding	62.75%	62.52%	1.06%
HTSeq_STAR		all	3.78%	3.61%	0.49%	all	62.23%	62.02%	1.04%
HTSeq_STAR		lncRNA	4.54%	4.39%	0.49%	proteincoding	62.48%	62.28%	1.06%
HTSeq_Subread	reversePRJEB11797	all	3.62%	3.51%	0.46%	all	62.07%	61.92%	1.00%
HTSeq_Subread		lncRNA	4.34%	4.24%	0.48%	proteincoding	62.31%	62.15%	1.02%
Kallisto		all	4.16%	3.98%	0.50%	all	64.20%	64.00%	1.07%
Kallisto		lncRNA	8.57%	8.45%	0.94%	proteincoding	64.51%	64.32%	1.08%
Salmon		all	4.25%	4.09%	0.49%	all	64.20%	63.99%	1.07%
Salmon		lncRNA	20.60%	20.61%	1.28%	proteincoding	64.70%	64.52%	1.09%
truth		truth	4.23%	4.03%	0.53%	truth	64.22%	64.02%	1.08%

The data was based on 10 samples from each library type

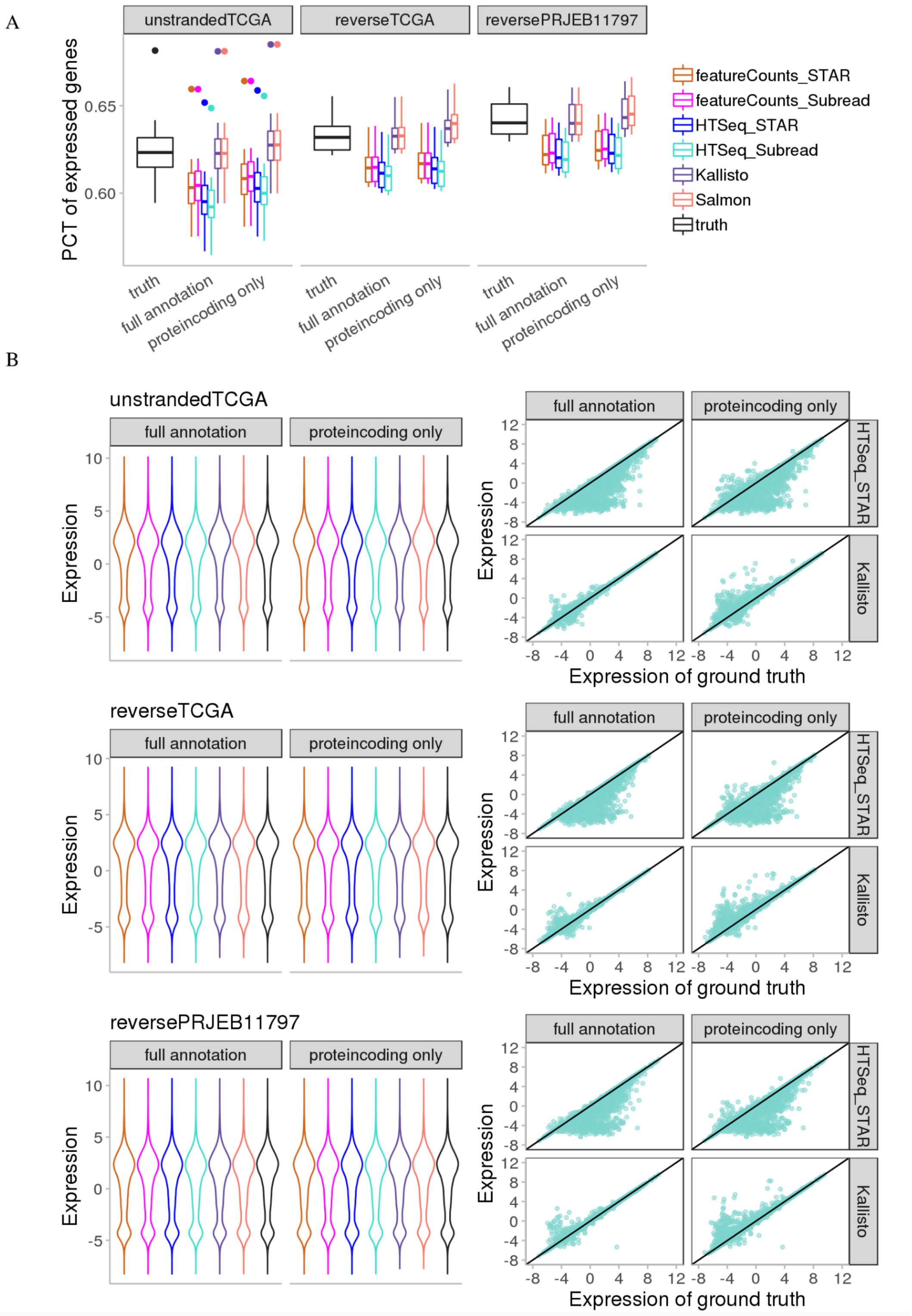

Additional file 3. The effect of incomplete transcriptome annotation on the expression quantification of protein-coding genes. **A)** Boxplot of the percentages of expressed protein-coding genes (FPKM>=1) detected with each tool, using full annotation or protein-coding-only annotation. The ground truth expression used for data simulation was also plotted for comparison. Each point in the boxplot represents one sample. For each library type, 10 samples were included in the analysis. **B)** The expression profile of protein-coding genes in one representative sample from each of the datasets was shown with violin plot (left) and scatter plot (right), which demonstrates the over-estimation effect using protein-coding-only annotation compared with full annotation, in all three samples for both pseudoalignment and alignment-based methods. PCT, percentage.

Additional file 4. The percentage of expressed lncRNA genes using each method and full annotation

library type	method	mean	median	sd	p value
reversePRJEB11797	featureCounts_HISAT2	4.25%	4.15%	1.28%	8.51E-02
	featureCounts_STAR	4.27%	4.16%	1.28%	9.61E-02
	featureCounts_Subread	4.23%	4.11%	1.26%	6.97E-02
	HTSeq_HISAT2	4.19%	4.05%	1.27%	4.98E-02
	HTSeq_STAR	4.22%	4.10%	1.28%	6.55E-02
	HTSeq_Subread	4.10%	3.96%	1.24%	2.08E-02
	Kallisto	4.62%	4.50%	1.34%	7.25E-01
	Salmon	4.69%	4.55%	1.34%	9.28E-01
	truth	4.68%	4.59%	1.36%	
reverseTCGA	featureCounts_HISAT2	6.79%	5.66%	3.04%	2.13E-01
	featureCounts_STAR	6.80%	5.65%	3.04%	2.20E-01
	featureCounts_Subread	6.73%	5.59%	3.01%	1.83E-01
	HTSeq_HISAT2	6.50%	5.39%	2.93%	9.03E-02
	HTSeq_STAR	6.71%	5.55%	3.03%	1.66E-01
	HTSeq_Subread	6.39%	5.28%	2.88%	6.68E-02
	Kallisto	7.34%	6.16%	3.15%	8.14E-01
	Salmon	7.47%	6.31%	3.16%	8.40E-01
	truth	7.40%	6.23%	3.17%	
unstrandedTCGA	featureCounts_HISAT2	4.95%	5.00%	1.28%	6.32E-13
	featureCounts_STAR	4.96%	4.99%	1.28%	6.79E-13
	featureCounts_Subread	4.94%	4.99%	1.26%	3.30E-13
	HTSeq_HISAT2	4.24%	4.23%	1.14%	1.88E-22
	HTSeq_STAR	4.34%	4.36%	1.17%	2.50E-21
	HTSeq_Subread	4.14%	4.15%	1.11%	8.98E-24
	Kallisto	6.60%	6.61%	1.52%	8.93E-01
	Salmon	6.66%	6.68%	1.52%	8.20E-01
	truth	6.61%	6.66%	1.47%	

The data was based on all the samples.
The p values were calculated from Mann-Whitney U test comparing the percentage of expressed genes from each tool and ground truth.

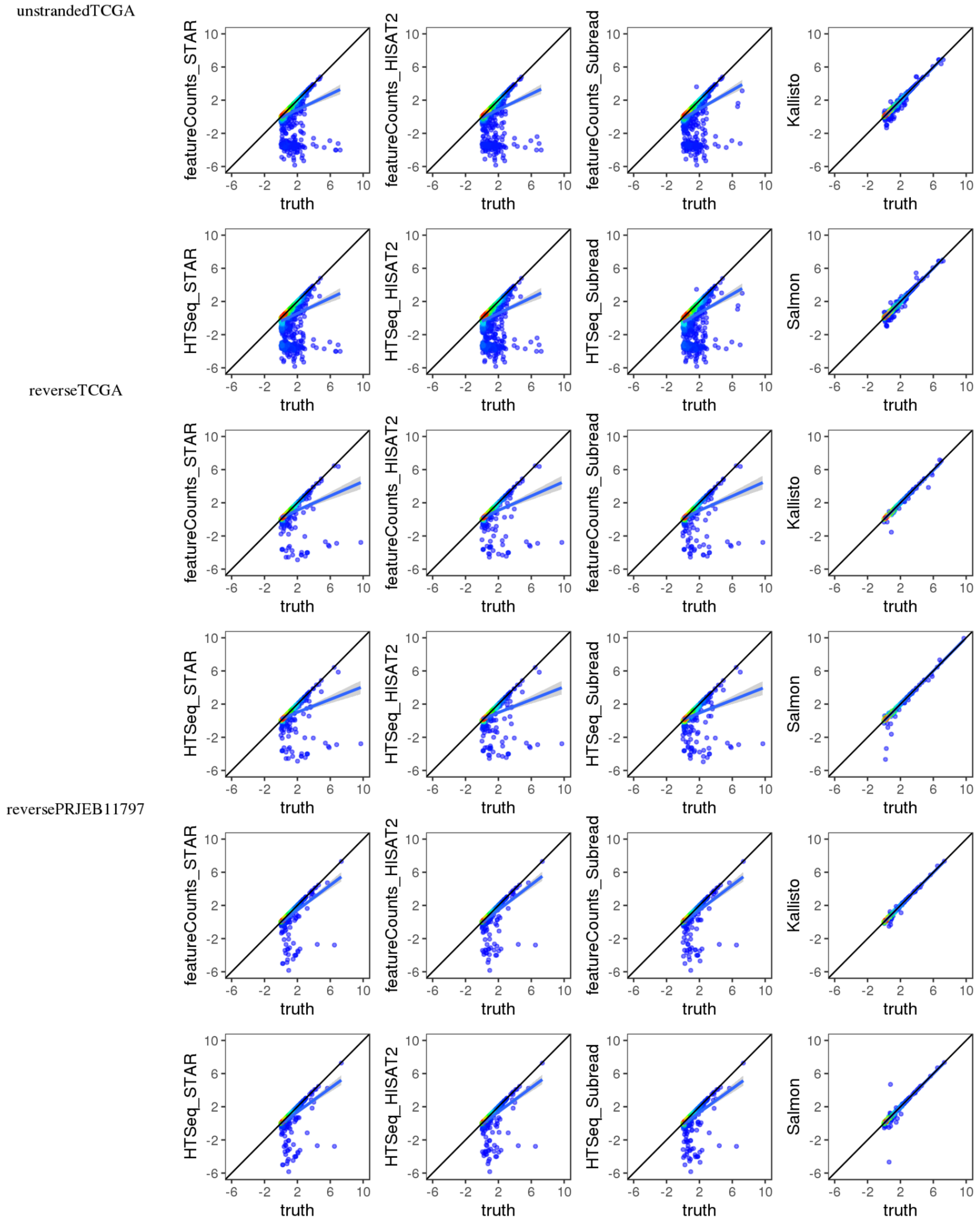

Additional file 5. Examples of sample-level comparison of each method and the ground truth. The expression profile of lncRNAs in one representative sample from each of the datasets was shown with scatter plot, which demonstrates the under-estimation effect of alignment-based methods, compared to the ground truth. Only expressed lncRNAs in the ground truth were included the analysis. The linear regression line between each method and the ground truth was plotted in blue.

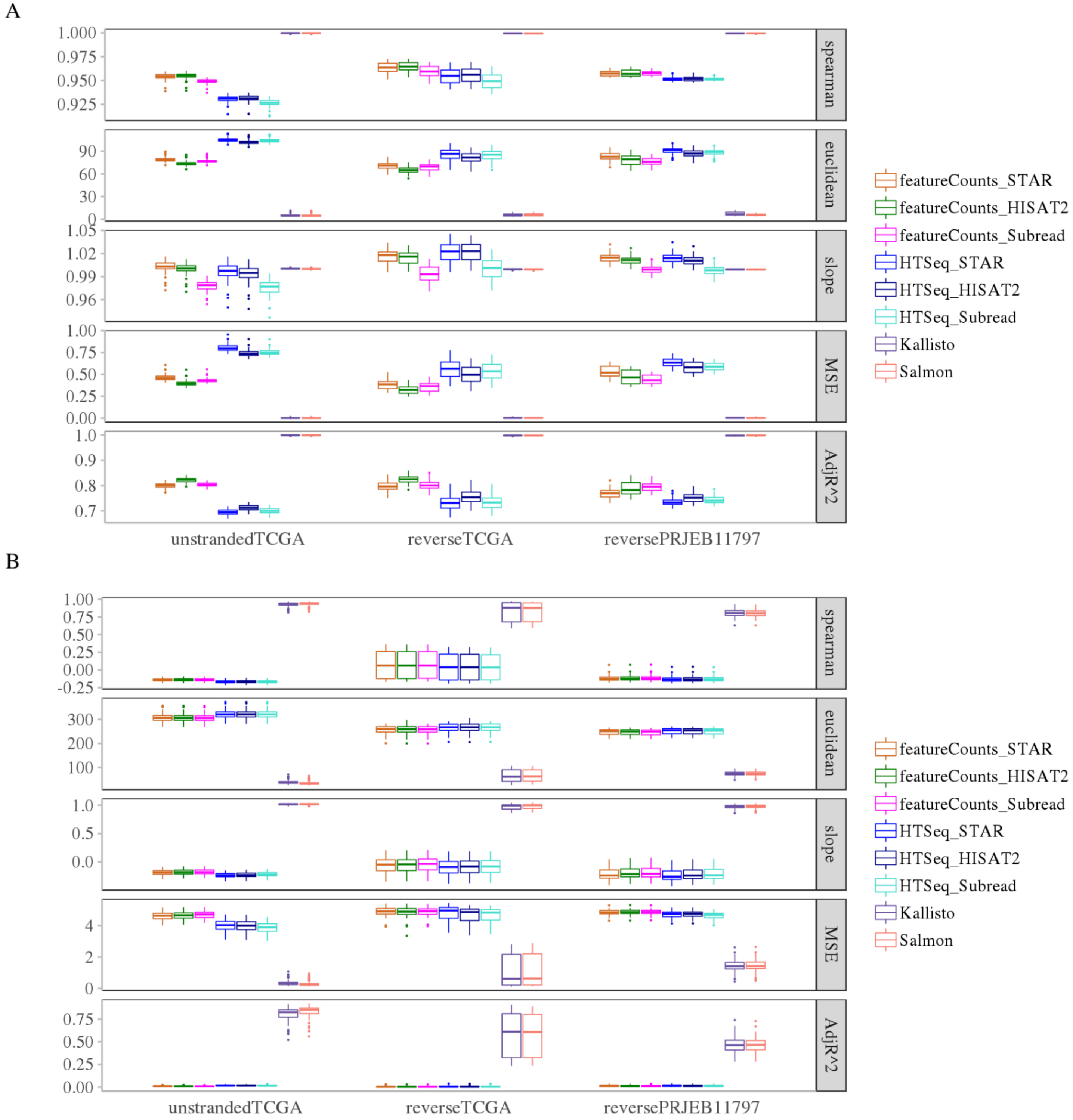

Additional file 6. Sample-level comparison of gene expression. Sample-level comparison for **A)** protein-coding genes in GENCODE and **B)** lncRNAs in GENCODE+NONCODE. The calculation of Spearman's correlation and Euclidean distance, and linear regression were performed using log-transformed FPKM values reported by each tool compared with ground truth. Only expressed lncRNAs in each sample were included in the analysis. Each point in the boxplot represents one sample. Spearman, Spearman's rank-order correlation; PCT, percentage; MSE, mean squared error; AdjR^2, adjusted R squared.

Additional file 7. Statistical tests for gene-level comparison between pseudoalignment methods and alignment-based methods

	Spearman's correlation			Euclidean distance			slope			MSE			AdjRSquared		
	unstrandedTCGA	reverseTCGA	reversePRJEB11797	unstrandedTCGA	reverseTCGA	reversePRJEB11797	unstrandedTCGA	reverseTCGA	reversePRJEB11797	unstrandedTCGA	reverseTCGA	reversePRJEB11797	unstrandedTCGA	reverseTCGA	reversePRJEB11797
Kallisto	/	/	/	/	/	/	/	/	/	/	/	/	/	/	/
Salmon	9.42E-01	4.70E-05	7.55E-05	8.71E-01	6.43E-07	6.40E-04	7.90E-01	4.96E-01	5.00E-01	9.46E-01	2.03E-07	1.01E-04	9.44E-01	1.59E-06	1.08E-04
featureCounts_STAR	6.29E-04	5.47E-05	2.39E-08	1.86E-12	9.92E-07	1.78E-07	7.01E-06	1.02E-07	1.19E-02	7.41E-03	5.42E-11	1.56E-09	8.63E-04	2.18E-08	1.90E-08
featureCounts_HISAT2	1.06E-03	2.83E-04	1.07E-03	3.40E-11	1.22E-07	6.81E-03	9.09E-08	7.79E-08	2.61E-06	1.17E-02	6.48E-11	3.57E-04	1.82E-03	1.38E-07	7.00E-04
featureCounts_Subread	1.40E-07	1.55E-04	1.14E-01	9.42E-21	4.55E-02	5.33E-01	3.79E-06	1.09E-07	8.06E-02	4.04E-06	1.05E-05	3.30E-02	1.36E-07	7.66E-05	6.77E-02
HTSeq_STAR	4.01E-17	3.97E-03	3.35E-05	1.20E-32	1.43E-04	3.10E-04	2.81E-03	3.56E-07	1.67E-03	5.01E-15	7.24E-08	9.43E-06	1.56E-17	7.18E-06	6.13E-05
HTSeq_HISAT2	3.55E-61	1.09E-80	3.13E-07	3.23E-94	5.70E-139	2.22E-09	2.92E-09	1.85E-24	9.68E-08	5.38E-56	4.72E-116	7.00E-09	3.77E-59	2.39E-96	1.06E-08
HTSeq_Subread	2.05E-56	5.64E-21	2.00E-32	1.09E-91	2.65E-39	2.78E-36	2.02E-09	2.05E-01	2.70E-05	9.56E-53	9.51E-25	2.74E-37	1.22E-54	2.19E-23	3.08E-34

The p values were calculated from Mann-Whitney U test comparing Kallisto output with other tools.

A

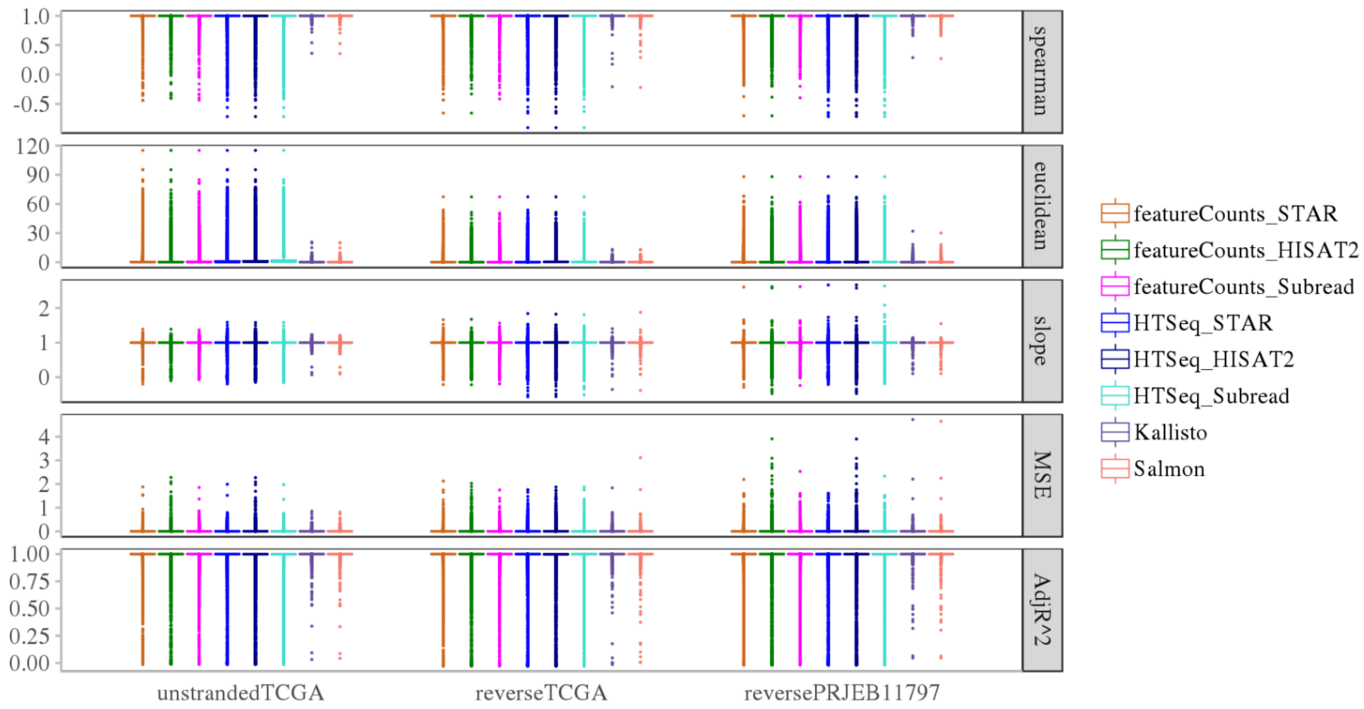

B

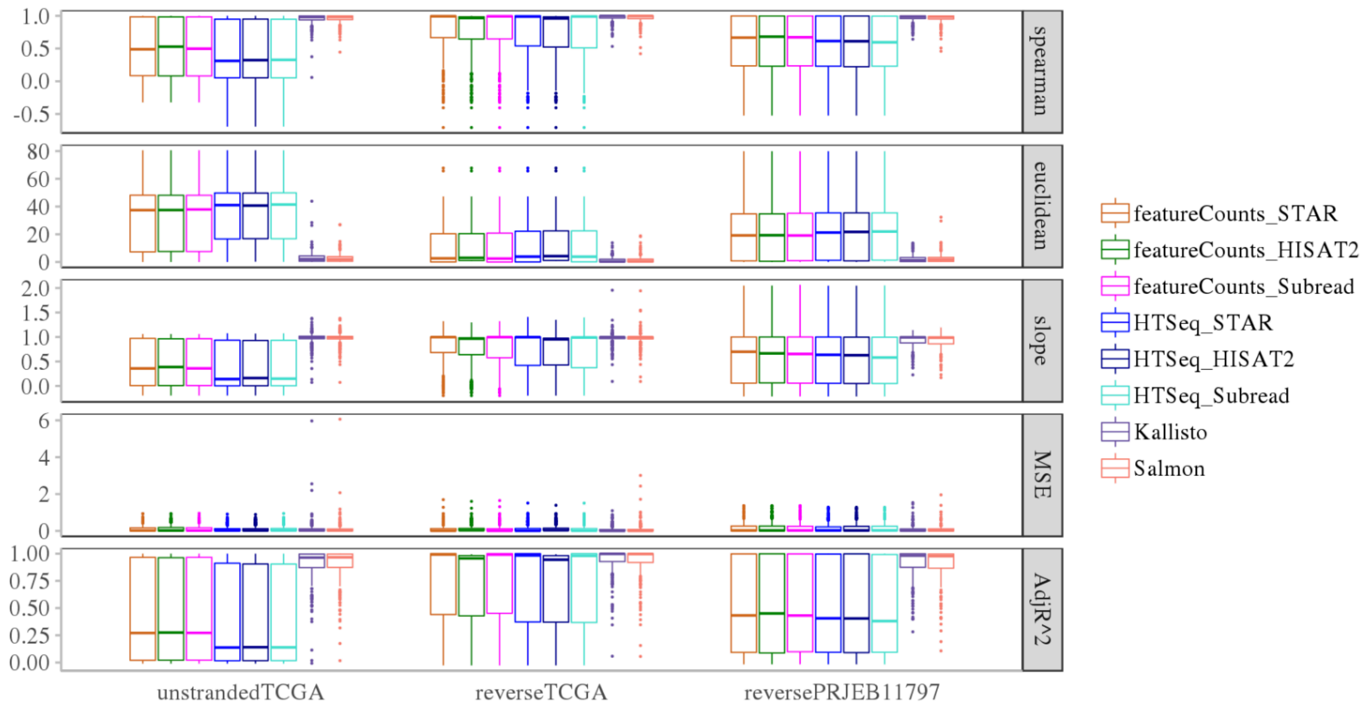

Additional file 8. Gene-level comparison of gene expression. Gene-level comparison for **A)** protein-coding genes in GENCODE and **B)** lncRNAs in GENCODE+NONCODE. The calculation of Spearman's correlation and Euclidean distance, and linear regression were performed using log-transformed FPKM values reported by each tool compared with the ground truth. Only lncRNAs with median FPKM value above one in the corresponding dataset were included in the analysis. Each point in the boxplot represents one gene. Spearman, Spearman's rank-order correlation; PCT, percentage; MSE, mean squared error; AdjR^2, adjusted R squared.

A

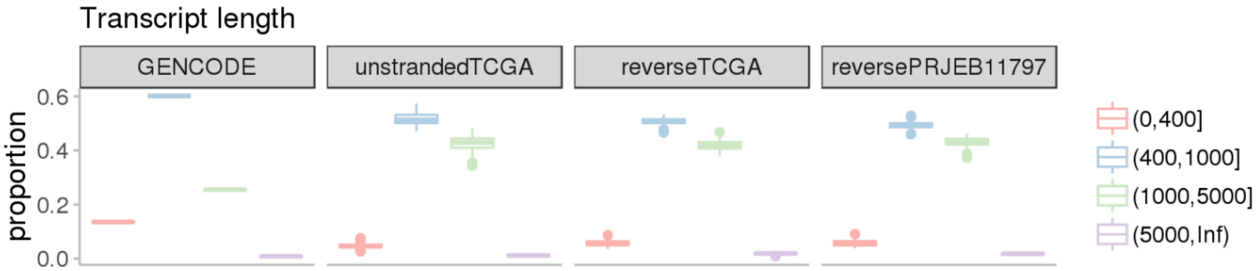

B

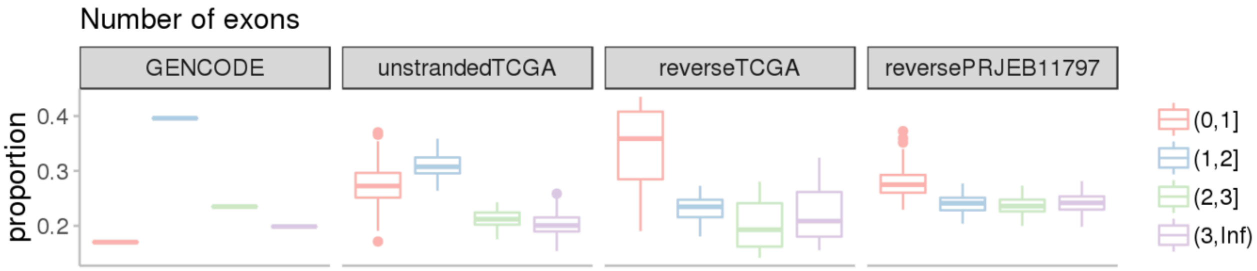

Additional file 9. Features of total and expressed lncRNAs. The proportion of **A)** the transcript length and **B)** the average number of exons of lncRNAs in GENCODE and expressed lncRNAs in samples from the three datasets. Each point in the boxplot represents one sample.

Additional file 10. Feature (lncRNA type) of discordant lncRNAs

methods	type	total	expressed	discordant	expressedPCT	discordantPCT	library
featureCounts_HISAT2	antisense	5521	390	81	7.06%	20.77%	unstrandedTCGA
featureCounts_STAR	antisense	5521	390	81	7.06%	20.77%	unstrandedTCGA
featureCounts_Subread	antisense	5521	390	79	7.06%	20.26%	unstrandedTCGA
HTSeq_HISAT2	antisense	5521	390	100	7.06%	25.64%	unstrandedTCGA
HTSeq_STAR	antisense	5521	390	101	7.06%	25.90%	unstrandedTCGA
HTSeq_Subread	antisense	5521	390	99	7.06%	25.38%	unstrandedTCGA
Kallisto	antisense	5521	390	3	7.06%	0.77%	unstrandedTCGA
Salmon	antisense	5521	390	3	7.06%	0.77%	unstrandedTCGA
featureCounts_HISAT2	lincRNA	7499	227	20	3.03%	8.81%	unstrandedTCGA
featureCounts_STAR	lincRNA	7499	227	19	3.03%	8.37%	unstrandedTCGA
featureCounts_Subread	lincRNA	7499	227	16	3.03%	7.05%	unstrandedTCGA
HTSeq_HISAT2	lincRNA	7499	227	22	3.03%	9.69%	unstrandedTCGA
HTSeq_STAR	lincRNA	7499	227	22	3.03%	9.69%	unstrandedTCGA
HTSeq_Subread	lincRNA	7499	227	18	3.03%	7.93%	unstrandedTCGA
Kallisto	lincRNA	7499	227	1	3.03%	0.44%	unstrandedTCGA
Salmon	lincRNA	7499	227	1	3.03%	0.44%	unstrandedTCGA
featureCounts_HISAT2	other	1148	47	11	4.09%	23.40%	unstrandedTCGA
featureCounts_STAR	other	1148	47	11	4.09%	23.40%	unstrandedTCGA
featureCounts_Subread	other	1148	47	11	4.09%	23.40%	unstrandedTCGA
HTSeq_HISAT2	other	1148	47	13	4.09%	27.66%	unstrandedTCGA
HTSeq_STAR	other	1148	47	13	4.09%	27.66%	unstrandedTCGA
HTSeq_Subread	other	1148	47	13	4.09%	27.66%	unstrandedTCGA
Kallisto	other	1148	47	0	4.09%	0.00%	unstrandedTCGA
Salmon	other	1148	47	0	4.09%	0.00%	unstrandedTCGA
featureCounts_HISAT2	antisense	5521	250	2	4.53%	0.80%	reverseTCGA
featureCounts_STAR	antisense	5521	250	3	4.53%	1.20%	reverseTCGA
featureCounts_Subread	antisense	5521	250	3	4.53%	1.20%	reverseTCGA
HTSeq_HISAT2	antisense	5521	250	2	4.53%	0.80%	reverseTCGA
HTSeq_STAR	antisense	5521	250	3	4.53%	1.20%	reverseTCGA
HTSeq_Subread	antisense	5521	250	3	4.53%	1.20%	reverseTCGA
Kallisto	antisense	5521	250	0	4.53%	0.00%	reverseTCGA
Salmon	antisense	5521	250	1	4.53%	0.40%	reverseTCGA
featureCounts_HISAT2	lincRNA	7499	287	16	3.83%	5.57%	reverseTCGA
featureCounts_STAR	lincRNA	7499	287	14	3.83%	4.88%	reverseTCGA
featureCounts_Subread	lincRNA	7499	287	16	3.83%	5.57%	reverseTCGA
HTSeq_HISAT2	lincRNA	7499	287	17	3.83%	5.92%	reverseTCGA
HTSeq_STAR	lincRNA	7499	287	15	3.83%	5.23%	reverseTCGA
HTSeq_Subread	lincRNA	7499	287	20	3.83%	6.97%	reverseTCGA
Kallisto	lincRNA	7499	287	0	3.83%	0.00%	reverseTCGA
Salmon	lincRNA	7499	287	0	3.83%	0.00%	reverseTCGA
featureCounts_HISAT2	other	1148	192	10	16.72%	5.21%	reverseTCGA
featureCounts_STAR	other	1148	192	10	16.72%	5.21%	reverseTCGA
featureCounts_Subread	other	1148	192	10	16.72%	5.21%	reverseTCGA
HTSeq_HISAT2	other	1148	192	11	16.72%	5.73%	reverseTCGA
HTSeq_STAR	other	1148	192	11	16.72%	5.73%	reverseTCGA
HTSeq_Subread	other	1148	192	11	16.72%	5.73%	reverseTCGA
Kallisto	other	1148	192	4	16.72%	2.08%	reverseTCGA
Salmon	other	1148	192	3	16.72%	1.56%	reverseTCGA
featureCounts_HISAT2	antisense	5521	201	1	3.64%	0.50%	reversePRJEB11797
featureCounts_STAR	antisense	5521	201	1	3.64%	0.50%	reversePRJEB11797
featureCounts_Subread	antisense	5521	201	1	3.64%	0.50%	reversePRJEB11797
HTSeq_HISAT2	antisense	5521	201	1	3.64%	0.50%	reversePRJEB11797
HTSeq_STAR	antisense	5521	201	0	3.64%	0.00%	reversePRJEB11797
HTSeq_Subread	antisense	5521	201	1	3.64%	0.50%	reversePRJEB11797
Kallisto	antisense	5521	201	0	3.64%	0.00%	reversePRJEB11797
Salmon	antisense	5521	201	1	3.64%	0.50%	reversePRJEB11797
featureCounts_HISAT2	lincRNA	7499	210	12	2.80%	5.71%	reversePRJEB11797
featureCounts_STAR	lincRNA	7499	210	10	2.80%	4.76%	reversePRJEB11797
featureCounts_Subread	lincRNA	7499	210	12	2.80%	5.71%	reversePRJEB11797
HTSeq_HISAT2	lincRNA	7499	210	14	2.80%	6.67%	reversePRJEB11797
HTSeq_STAR	lincRNA	7499	210	11	2.80%	5.24%	reversePRJEB11797
HTSeq_Subread	lincRNA	7499	210	15	2.80%	7.14%	reversePRJEB11797
Kallisto	lincRNA	7499	210	1	2.80%	0.48%	reversePRJEB11797
Salmon	lincRNA	7499	210	1	2.80%	0.48%	reversePRJEB11797
featureCounts_HISAT2	other	1148	53	6	4.62%	11.32%	reversePRJEB11797
featureCounts_STAR	other	1148	53	6	4.62%	11.32%	reversePRJEB11797
featureCounts_Subread	other	1148	53	6	4.62%	11.32%	reversePRJEB11797
HTSeq_HISAT2	other	1148	53	7	4.62%	13.21%	reversePRJEB11797
HTSeq_STAR	other	1148	53	7	4.62%	13.21%	reversePRJEB11797
HTSeq_Subread	other	1148	53	6	4.62%	11.32%	reversePRJEB11797
Kallisto	other	1148	53	0	4.62%	0.00%	reversePRJEB11797
Salmon	other	1148	53	0	4.62%	0.00%	reversePRJEB11797

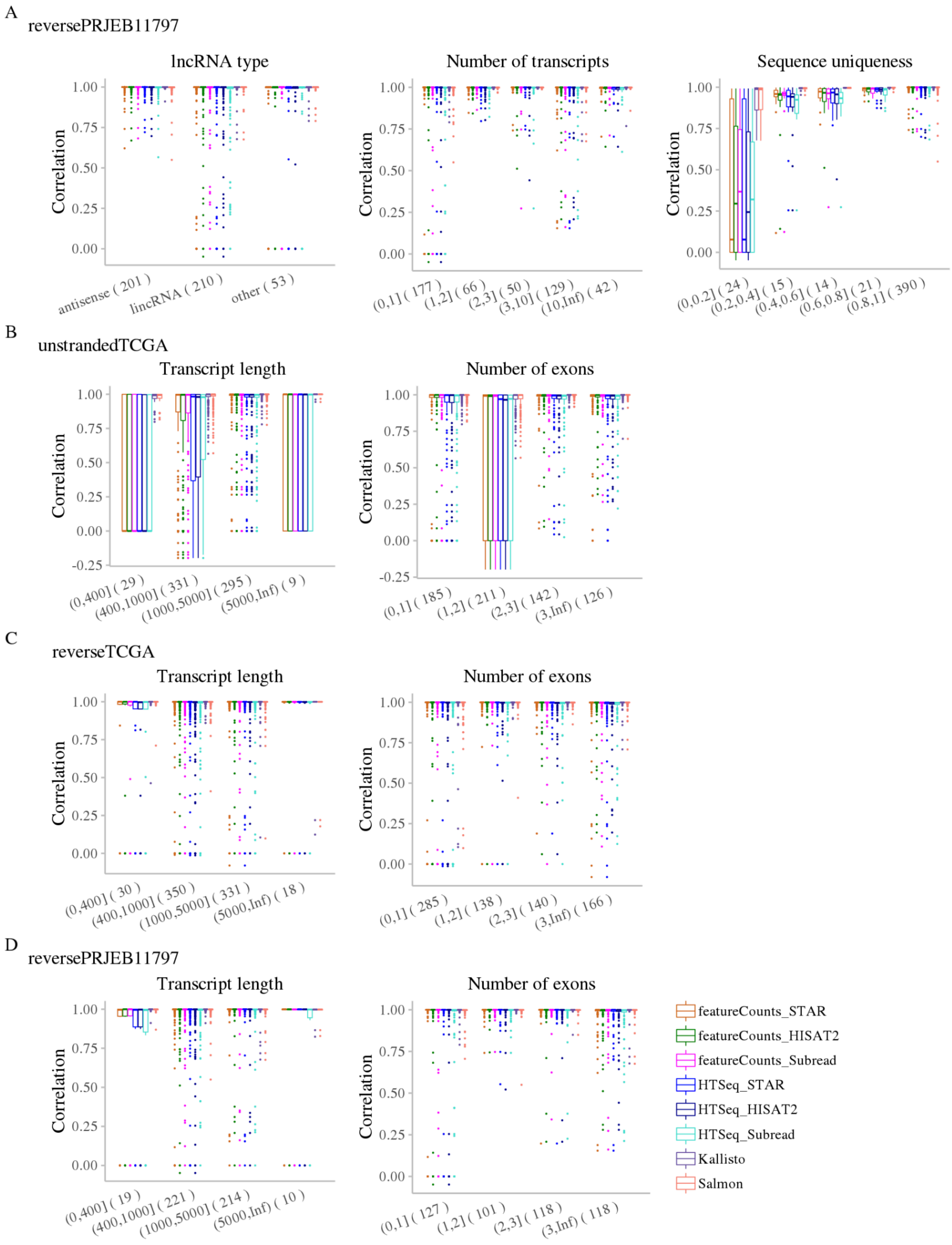

Additional file 11. Features of discordant lncRNAs. **A)** The lncRNA type, number of transcripts, and sequence uniqueness of expressed lncRNAs (median FPKM above one) in the reversePRJEB11797 dataset. The transcript length and number of exons for expressed lncRNAs for the three datasets are shown in **B-D**. Spearman's correlation was calculated comparing each method and the ground truth. Each point in the boxplot represents one gene. Numbers in brackets in the x-axis labels are the number of genes in certain category.

Additional file 12. Feature (number of transcripts) of discordant lncRNAs

methods	type	total	expressed	discordant	expressedPCT	discordantPCT	library
featureCounts_HISAT2	(0,1]	10703	312	86	2.92%	27.56%	unstrandedTCGA
featureCounts_STAR	(0,1]	10703	312	87	2.92%	27.88%	unstrandedTCGA
featureCounts_Subread	(0,1]	10703	312	82	2.92%	26.28%	unstrandedTCGA
HTSeq_HISAT2	(0,1]	10703	312	100	2.92%	32.05%	unstrandedTCGA
HTSeq_STAR	(0,1]	10703	312	101	2.92%	32.37%	unstrandedTCGA
HTSeq_Subread	(0,1]	10703	312	95	2.92%	30.45%	unstrandedTCGA
Kallisto	(0,1]	10703	312	2	2.92%	0.64%	unstrandedTCGA
Salmon	(0,1]	10703	312	2	2.92%	0.64%	unstrandedTCGA
featureCounts_HISAT2	(1,2]	1798	94	12	5.23%	12.77%	unstrandedTCGA
featureCounts_STAR	(1,2]	1798	94	12	5.23%	12.77%	unstrandedTCGA
featureCounts_Subread	(1,2]	1798	94	11	5.23%	11.70%	unstrandedTCGA
HTSeq_HISAT2	(1,2]	1798	94	18	5.23%	19.15%	unstrandedTCGA
HTSeq_STAR	(1,2]	1798	94	19	5.23%	20.21%	unstrandedTCGA
HTSeq_Subread	(1,2]	1798	94	18	5.23%	19.15%	unstrandedTCGA
Kallisto	(1,2]	1798	94	0	5.23%	0.00%	unstrandedTCGA
Salmon	(1,2]	1798	94	0	5.23%	0.00%	unstrandedTCGA
featureCounts_HISAT2	(2,3]	708	64	4	9.04%	6.25%	unstrandedTCGA
featureCounts_STAR	(2,3]	708	64	3	9.04%	4.69%	unstrandedTCGA
featureCounts_Subread	(2,3]	708	64	3	9.04%	4.69%	unstrandedTCGA
HTSeq_HISAT2	(2,3]	708	64	5	9.04%	7.81%	unstrandedTCGA
HTSeq_STAR	(2,3]	708	64	5	9.04%	7.81%	unstrandedTCGA
HTSeq_Subread	(2,3]	708	64	5	9.04%	7.81%	unstrandedTCGA
Kallisto	(2,3]	708	64	1	9.04%	1.56%	unstrandedTCGA
Salmon	(2,3]	708	64	1	9.04%	1.56%	unstrandedTCGA
featureCounts_HISAT2	(3,10]	807	146	9	18.09%	6.16%	unstrandedTCGA
featureCounts_STAR	(3,10]	807	146	8	18.09%	5.48%	unstrandedTCGA
featureCounts_Subread	(3,10]	807	146	9	18.09%	6.16%	unstrandedTCGA
HTSeq_HISAT2	(3,10]	807	146	10	18.09%	6.85%	unstrandedTCGA
HTSeq_STAR	(3,10]	807	146	9	18.09%	6.16%	unstrandedTCGA
HTSeq_Subread	(3,10]	807	146	10	18.09%	6.85%	unstrandedTCGA
Kallisto	(3,10]	807	146	1	18.09%	0.68%	unstrandedTCGA
Salmon	(3,10]	807	146	1	18.09%	0.68%	unstrandedTCGA
featureCounts_HISAT2	(10,Inf)	152	48	1	31.58%	2.08%	unstrandedTCGA
featureCounts_STAR	(10,Inf)	152	48	1	31.58%	2.08%	unstrandedTCGA
featureCounts_Subread	(10,Inf)	152	48	1	31.58%	2.08%	unstrandedTCGA
HTSeq_HISAT2	(10,Inf)	152	48	2	31.58%	4.17%	unstrandedTCGA
HTSeq_STAR	(10,Inf)	152	48	2	31.58%	4.17%	unstrandedTCGA
HTSeq_Subread	(10,Inf)	152	48	2	31.58%	4.17%	unstrandedTCGA
Kallisto	(10,Inf)	152	48	0	31.58%	0.00%	unstrandedTCGA
Salmon	(10,Inf)	152	48	0	31.58%	0.00%	unstrandedTCGA
featureCounts_HISAT2	(0,1]	10703	373	19	3.49%	5.09%	reverseTCGA
featureCounts_STAR	(0,1]	10703	373	19	3.49%	5.09%	reverseTCGA
featureCounts_Subread	(0,1]	10703	373	20	3.49%	5.36%	reverseTCGA
HTSeq_HISAT2	(0,1]	10703	373	21	3.49%	5.63%	reverseTCGA
HTSeq_STAR	(0,1]	10703	373	21	3.49%	5.63%	reverseTCGA
HTSeq_Subread	(0,1]	10703	373	23	3.49%	6.17%	reverseTCGA
Kallisto	(0,1]	10703	373	4	3.49%	1.07%	reverseTCGA
Salmon	(0,1]	10703	373	3	3.49%	0.80%	reverseTCGA
featureCounts_HISAT2	(1,2]	1798	69	0	3.84%	0.00%	reverseTCGA
featureCounts_STAR	(1,2]	1798	69	0	3.84%	0.00%	reverseTCGA
featureCounts_Subread	(1,2]	1798	69	0	3.84%	0.00%	reverseTCGA
HTSeq_HISAT2	(1,2]	1798	69	0	3.84%	0.00%	reverseTCGA
HTSeq_STAR	(1,2]	1798	69	0	3.84%	0.00%	reverseTCGA
HTSeq_Subread	(1,2]	1798	69	0	3.84%	0.00%	reverseTCGA
Kallisto	(1,2]	1798	69	0	3.84%	0.00%	reverseTCGA
Salmon	(1,2]	1798	69	0	3.84%	0.00%	reverseTCGA
featureCounts_HISAT2	(2,3]	708	59	2	8.33%	3.39%	reverseTCGA
featureCounts_STAR	(2,3]	708	59	0	8.33%	0.00%	reverseTCGA
featureCounts_Subread	(2,3]	708	59	1	8.33%	1.69%	reverseTCGA
HTSeq_HISAT2	(2,3]	708	59	2	8.33%	3.39%	reverseTCGA
HTSeq_STAR	(2,3]	708	59	0	8.33%	0.00%	reverseTCGA
HTSeq_Subread	(2,3]	708	59	1	8.33%	1.69%	reverseTCGA
Kallisto	(2,3]	708	59	0	8.33%	0.00%	reverseTCGA
Salmon	(2,3]	708	59	0	8.33%	0.00%	reverseTCGA

featureCounts_HISAT2	(3,10]	807	171	6	21.19%	3.51%	reverseTCGA
featureCounts_STAR	(3,10]	807	171	7	21.19%	4.09%	reverseTCGA
featureCounts_Subread	(3,10]	807	171	7	21.19%	4.09%	reverseTCGA
HTSeq_HISAT2	(3,10]	807	171	6	21.19%	3.51%	reverseTCGA
HTSeq_STAR	(3,10]	807	171	7	21.19%	4.09%	reverseTCGA
HTSeq_Subread	(3,10]	807	171	9	21.19%	5.26%	reverseTCGA
Kallisto	(3,10]	807	171	0	21.19%	0.00%	reverseTCGA
Salmon	(3,10]	807	171	1	21.19%	0.58%	reverseTCGA
featureCounts_HISAT2	(10,Inf)	152	57	1	37.50%	1.75%	reverseTCGA
featureCounts_STAR	(10,Inf)	152	57	1	37.50%	1.75%	reverseTCGA
featureCounts_Subread	(10,Inf)	152	57	1	37.50%	1.75%	reverseTCGA
HTSeq_HISAT2	(10,Inf)	152	57	1	37.50%	1.75%	reverseTCGA
HTSeq_STAR	(10,Inf)	152	57	1	37.50%	1.75%	reverseTCGA
HTSeq_Subread	(10,Inf)	152	57	1	37.50%	1.75%	reverseTCGA
Kallisto	(10,Inf)	152	57	0	37.50%	0.00%	reverseTCGA
Salmon	(10,Inf)	152	57	0	37.50%	0.00%	reverseTCGA
featureCounts_HISAT2	(0,1]	10703	177	12	1.65%	6.78%	reversePRJEB11797
featureCounts_STAR	(0,1]	10703	177	13	1.65%	7.34%	reversePRJEB11797
featureCounts_Subread	(0,1]	10703	177	13	1.65%	7.34%	reversePRJEB11797
HTSeq_HISAT2	(0,1]	10703	177	14	1.65%	7.91%	reversePRJEB11797
HTSeq_STAR	(0,1]	10703	177	14	1.65%	7.91%	reversePRJEB11797
HTSeq_Subread	(0,1]	10703	177	13	1.65%	7.34%	reversePRJEB11797
Kallisto	(0,1]	10703	177	0	1.65%	0.00%	reversePRJEB11797
Salmon	(0,1]	10703	177	1	1.65%	0.56%	reversePRJEB11797
featureCounts_HISAT2	(1,2]	1798	66	0	3.67%	0.00%	reversePRJEB11797
featureCounts_STAR	(1,2]	1798	66	0	3.67%	0.00%	reversePRJEB11797
featureCounts_Subread	(1,2]	1798	66	0	3.67%	0.00%	reversePRJEB11797
HTSeq_HISAT2	(1,2]	1798	66	0	3.67%	0.00%	reversePRJEB11797
HTSeq_STAR	(1,2]	1798	66	0	3.67%	0.00%	reversePRJEB11797
HTSeq_Subread	(1,2]	1798	66	0	3.67%	0.00%	reversePRJEB11797
Kallisto	(1,2]	1798	66	0	3.67%	0.00%	reversePRJEB11797
Salmon	(1,2]	1798	66	0	3.67%	0.00%	reversePRJEB11797
featureCounts_HISAT2	(2,3]	708	50	1	7.06%	2.00%	reversePRJEB11797
featureCounts_STAR	(2,3]	708	50	0	7.06%	0.00%	reversePRJEB11797
featureCounts_Subread	(2,3]	708	50	1	7.06%	2.00%	reversePRJEB11797
HTSeq_HISAT2	(2,3]	708	50	1	7.06%	2.00%	reversePRJEB11797
HTSeq_STAR	(2,3]	708	50	0	7.06%	0.00%	reversePRJEB11797
HTSeq_Subread	(2,3]	708	50	2	7.06%	4.00%	reversePRJEB11797
Kallisto	(2,3]	708	50	0	7.06%	0.00%	reversePRJEB11797
Salmon	(2,3]	708	50	0	7.06%	0.00%	reversePRJEB11797
featureCounts_HISAT2	(3,10]	807	129	5	15.99%	3.88%	reversePRJEB11797
featureCounts_STAR	(3,10]	807	129	4	15.99%	3.10%	reversePRJEB11797
featureCounts_Subread	(3,10]	807	129	5	15.99%	3.88%	reversePRJEB11797
HTSeq_HISAT2	(3,10]	807	129	6	15.99%	4.65%	reversePRJEB11797
HTSeq_STAR	(3,10]	807	129	4	15.99%	3.10%	reversePRJEB11797
HTSeq_Subread	(3,10]	807	129	6	15.99%	4.65%	reversePRJEB11797
Kallisto	(3,10]	807	129	1	15.99%	0.78%	reversePRJEB11797
Salmon	(3,10]	807	129	1	15.99%	0.78%	reversePRJEB11797
featureCounts_HISAT2	(10,Inf)	152	42	1	27.63%	2.38%	reversePRJEB11797
featureCounts_STAR	(10,Inf)	152	42	0	27.63%	0.00%	reversePRJEB11797
featureCounts_Subread	(10,Inf)	152	42	0	27.63%	0.00%	reversePRJEB11797
HTSeq_HISAT2	(10,Inf)	152	42	1	27.63%	2.38%	reversePRJEB11797
HTSeq_STAR	(10,Inf)	152	42	0	27.63%	0.00%	reversePRJEB11797
HTSeq_Subread	(10,Inf)	152	42	1	27.63%	2.38%	reversePRJEB11797
Kallisto	(10,Inf)	152	42	0	27.63%	0.00%	reversePRJEB11797
Salmon	(10,Inf)	152	42	0	27.63%	0.00%	reversePRJEB11797

Additional file 13. Feature (transcript length) of discordant lncRNAs

methods	type	total	expressed	discordant	expressedPCT	discordantPCT	library
featureCounts_HISAT2	(0,400]	1919	29	16	1.51%	55.17%	unstrandedTCGA
featureCounts_STAR	(0,400]	1919	29	15	1.51%	51.72%	unstrandedTCGA
featureCounts_Subread	(0,400]	1919	29	16	1.51%	55.17%	unstrandedTCGA
HTSeq_HISAT2	(0,400]	1919	29	16	1.51%	55.17%	unstrandedTCGA
HTSeq_STAR	(0,400]	1919	29	15	1.51%	51.72%	unstrandedTCGA
HTSeq_Subread	(0,400]	1919	29	16	1.51%	55.17%	unstrandedTCGA
Kallisto	(0,400]	1919	29	0	1.51%	0.00%	unstrandedTCGA
Salmon	(0,400]	1919	29	0	1.51%	0.00%	unstrandedTCGA
featureCounts_HISAT2	(400,1000]	8524	331	78	3.88%	23.56%	unstrandedTCGA
featureCounts_STAR	(400,1000]	8524	331	76	3.88%	22.96%	unstrandedTCGA
featureCounts_Subread	(400,1000]	8524	331	72	3.88%	21.75%	unstrandedTCGA
HTSeq_HISAT2	(400,1000]	8524	331	92	3.88%	27.79%	unstrandedTCGA
HTSeq_STAR	(400,1000]	8524	331	91	3.88%	27.49%	unstrandedTCGA
HTSeq_Subread	(400,1000]	8524	331	88	3.88%	26.59%	unstrandedTCGA
Kallisto	(400,1000]	8524	331	4	3.88%	1.21%	unstrandedTCGA
Salmon	(400,1000]	8524	331	4	3.88%	1.21%	unstrandedTCGA
featureCounts_HISAT2	(1000,5000]	3609	295	15	8.17%	5.08%	unstrandedTCGA
featureCounts_STAR	(1000,5000]	3609	295	17	8.17%	5.76%	unstrandedTCGA
featureCounts_Subread	(1000,5000]	3609	295	15	8.17%	5.08%	unstrandedTCGA
HTSeq_HISAT2	(1000,5000]	3609	295	24	8.17%	8.14%	unstrandedTCGA
HTSeq_STAR	(1000,5000]	3609	295	27	8.17%	9.15%	unstrandedTCGA
HTSeq_Subread	(1000,5000]	3609	295	23	8.17%	7.80%	unstrandedTCGA
Kallisto	(1000,5000]	3609	295	0	8.17%	0.00%	unstrandedTCGA
Salmon	(1000,5000]	3609	295	0	8.17%	0.00%	unstrandedTCGA
featureCounts_HISAT2	(5000,Inf)	116	9	3	7.76%	33.33%	unstrandedTCGA
featureCounts_STAR	(5000,Inf)	116	9	3	7.76%	33.33%	unstrandedTCGA
featureCounts_Subread	(5000,Inf)	116	9	3	7.76%	33.33%	unstrandedTCGA
HTSeq_HISAT2	(5000,Inf)	116	9	3	7.76%	33.33%	unstrandedTCGA
HTSeq_STAR	(5000,Inf)	116	9	3	7.76%	33.33%	unstrandedTCGA
HTSeq_Subread	(5000,Inf)	116	9	3	7.76%	33.33%	unstrandedTCGA
Kallisto	(5000,Inf)	116	9	0	7.76%	0.00%	unstrandedTCGA
Salmon	(5000,Inf)	116	9	0	7.76%	0.00%	unstrandedTCGA
featureCounts_HISAT2	(0,400]	1919	30	6	1.56%	20.00%	reverseTCGA
featureCounts_STAR	(0,400]	1919	30	5	1.56%	16.67%	reverseTCGA
featureCounts_Subread	(0,400]	1919	30	6	1.56%	20.00%	reverseTCGA
HTSeq_HISAT2	(0,400]	1919	30	6	1.56%	20.00%	reverseTCGA
HTSeq_STAR	(0,400]	1919	30	5	1.56%	16.67%	reverseTCGA
HTSeq_Subread	(0,400]	1919	30	6	1.56%	20.00%	reverseTCGA
Kallisto	(0,400]	1919	30	1	1.56%	3.33%	reverseTCGA
Salmon	(0,400]	1919	30	0	1.56%	0.00%	reverseTCGA
featureCounts_HISAT2	(400,1000]	8524	350	13	4.11%	3.71%	reverseTCGA
featureCounts_STAR	(400,1000]	8524	350	11	4.11%	3.14%	reverseTCGA
featureCounts_Subread	(400,1000]	8524	350	11	4.11%	3.14%	reverseTCGA
HTSeq_HISAT2	(400,1000]	8524	350	15	4.11%	4.29%	reverseTCGA
HTSeq_STAR	(400,1000]	8524	350	13	4.11%	3.71%	reverseTCGA
HTSeq_Subread	(400,1000]	8524	350	15	4.11%	4.29%	reverseTCGA
Kallisto	(400,1000]	8524	350	0	4.11%	0.00%	reverseTCGA
Salmon	(400,1000]	8524	350	1	4.11%	0.29%	reverseTCGA

featureCounts_HISAT2	(1000,5000]	3609	331	7	9.17%	2.11%	reverseTCGA
featureCounts_STAR	(1000,5000]	3609	331	9	9.17%	2.72%	reverseTCGA
featureCounts_Subread	(1000,5000]	3609	331	10	9.17%	3.02%	reverseTCGA
HTSeq_HISAT2	(1000,5000]	3609	331	7	9.17%	2.11%	reverseTCGA
HTSeq_STAR	(1000,5000]	3609	331	9	9.17%	2.72%	reverseTCGA
HTSeq_Subread	(1000,5000]	3609	331	11	9.17%	3.32%	reverseTCGA
Kallisto	(1000,5000]	3609	331	1	9.17%	0.30%	reverseTCGA
Salmon	(1000,5000]	3609	331	1	9.17%	0.30%	reverseTCGA
featureCounts_HISAT2	(5000,Inf)	116	18	2	15.52%	11.11%	reverseTCGA
featureCounts_STAR	(5000,Inf)	116	18	2	15.52%	11.11%	reverseTCGA
featureCounts_Subread	(5000,Inf)	116	18	2	15.52%	11.11%	reverseTCGA
HTSeq_HISAT2	(5000,Inf)	116	18	2	15.52%	11.11%	reverseTCGA
HTSeq_STAR	(5000,Inf)	116	18	2	15.52%	11.11%	reverseTCGA
HTSeq_Subread	(5000,Inf)	116	18	2	15.52%	11.11%	reverseTCGA
Kallisto	(5000,Inf)	116	18	2	15.52%	11.11%	reverseTCGA
Salmon	(5000,Inf)	116	18	2	15.52%	11.11%	reverseTCGA
featureCounts_HISAT2	(0,400]	1919	19	4	0.99%	21.05%	reversePRJEB11797
featureCounts_STAR	(0,400]	1919	19	4	0.99%	21.05%	reversePRJEB11797
featureCounts_Subread	(0,400]	1919	19	4	0.99%	21.05%	reversePRJEB11797
HTSeq_HISAT2	(0,400]	1919	19	4	0.99%	21.05%	reversePRJEB11797
HTSeq_STAR	(0,400]	1919	19	4	0.99%	21.05%	reversePRJEB11797
HTSeq_Subread	(0,400]	1919	19	4	0.99%	21.05%	reversePRJEB11797
Kallisto	(0,400]	1919	19	0	0.99%	0.00%	reversePRJEB11797
Salmon	(0,400]	1919	19	0	0.99%	0.00%	reversePRJEB11797
featureCounts_HISAT2	(400,1000]	8524	221	7	2.59%	3.17%	reversePRJEB11797
featureCounts_STAR	(400,1000]	8524	221	7	2.59%	3.17%	reversePRJEB11797
featureCounts_Subread	(400,1000]	8524	221	8	2.59%	3.62%	reversePRJEB11797
HTSeq_HISAT2	(400,1000]	8524	221	10	2.59%	4.52%	reversePRJEB11797
HTSeq_STAR	(400,1000]	8524	221	8	2.59%	3.62%	reversePRJEB11797
HTSeq_Subread	(400,1000]	8524	221	9	2.59%	4.07%	reversePRJEB11797
Kallisto	(400,1000]	8524	221	0	2.59%	0.00%	reversePRJEB11797
Salmon	(400,1000]	8524	221	1	2.59%	0.45%	reversePRJEB11797
featureCounts_HISAT2	(1000,5000]	3609	214	6	5.93%	2.80%	reversePRJEB11797
featureCounts_STAR	(1000,5000]	3609	214	4	5.93%	1.87%	reversePRJEB11797
featureCounts_Subread	(1000,5000]	3609	214	5	5.93%	2.34%	reversePRJEB11797
HTSeq_HISAT2	(1000,5000]	3609	214	6	5.93%	2.80%	reversePRJEB11797
HTSeq_STAR	(1000,5000]	3609	214	4	5.93%	1.87%	reversePRJEB11797
HTSeq_Subread	(1000,5000]	3609	214	7	5.93%	3.27%	reversePRJEB11797
Kallisto	(1000,5000]	3609	214	1	5.93%	0.47%	reversePRJEB11797
Salmon	(1000,5000]	3609	214	1	5.93%	0.47%	reversePRJEB11797
featureCounts_HISAT2	(5000,Inf)	116	10	2	8.62%	20.00%	reversePRJEB11797
featureCounts_STAR	(5000,Inf)	116	10	2	8.62%	20.00%	reversePRJEB11797
featureCounts_Subread	(5000,Inf)	116	10	2	8.62%	20.00%	reversePRJEB11797
HTSeq_HISAT2	(5000,Inf)	116	10	2	8.62%	20.00%	reversePRJEB11797
HTSeq_STAR	(5000,Inf)	116	10	2	8.62%	20.00%	reversePRJEB11797
HTSeq_Subread	(5000,Inf)	116	10	2	8.62%	20.00%	reversePRJEB11797
Kallisto	(5000,Inf)	116	10	0	8.62%	0.00%	reversePRJEB11797
Salmon	(5000,Inf)	116	10	0	8.62%	0.00%	reversePRJEB11797

Additional file 14. Feature (number of exons) of discordant lncRNAs

methods	type	total	expressed	discordant	expressedPCT	discordantPCT	library
featureCounts_HISAT2	(0,1]	2413	185	24	7.67%	12.97%	unstrandedTCGA
featureCounts_STAR	(0,1]	2413	185	25	7.67%	13.51%	unstrandedTCGA
featureCounts_Subread	(0,1]	2413	185	22	7.67%	11.89%	unstrandedTCGA
HTSeq_HISAT2	(0,1]	2413	185	33	7.67%	17.84%	unstrandedTCGA
HTSeq_STAR	(0,1]	2413	185	34	7.67%	18.38%	unstrandedTCGA
HTSeq_Subread	(0,1]	2413	185	31	7.67%	16.76%	unstrandedTCGA
Kallisto	(0,1]	2413	185	0	7.67%	0.00%	unstrandedTCGA
Salmon	(0,1]	2413	185	0	7.67%	0.00%	unstrandedTCGA
featureCounts_HISAT2	(1,2]	5610	211	75	3.76%	35.55%	unstrandedTCGA
featureCounts_STAR	(1,2]	5610	211	75	3.76%	35.55%	unstrandedTCGA
featureCounts_Subread	(1,2]	5610	211	73	3.76%	34.60%	unstrandedTCGA
HTSeq_HISAT2	(1,2]	5610	211	84	3.76%	39.81%	unstrandedTCGA
HTSeq_STAR	(1,2]	5610	211	84	3.76%	39.81%	unstrandedTCGA
HTSeq_Subread	(1,2]	5610	211	83	3.76%	39.34%	unstrandedTCGA
Kallisto	(1,2]	5610	211	3	3.76%	1.42%	unstrandedTCGA
Salmon	(1,2]	5610	211	3	3.76%	1.42%	unstrandedTCGA
featureCounts_HISAT2	(2,3]	3330	142	6	4.26%	4.23%	unstrandedTCGA
featureCounts_STAR	(2,3]	3330	142	3	4.26%	2.11%	unstrandedTCGA
featureCounts_Subread	(2,3]	3330	142	5	4.26%	3.52%	unstrandedTCGA
HTSeq_HISAT2	(2,3]	3330	142	8	4.26%	5.63%	unstrandedTCGA
HTSeq_STAR	(2,3]	3330	142	7	4.26%	4.93%	unstrandedTCGA
HTSeq_Subread	(2,3]	3330	142	8	4.26%	5.63%	unstrandedTCGA
Kallisto	(2,3]	3330	142	0	4.26%	0.00%	unstrandedTCGA
Salmon	(2,3]	3330	142	0	4.26%	0.00%	unstrandedTCGA
featureCounts_HISAT2	(3,Inf)	2815	126	7	4.48%	5.56%	unstrandedTCGA
featureCounts_STAR	(3,Inf)	2815	126	8	4.48%	6.35%	unstrandedTCGA
featureCounts_Subread	(3,Inf)	2815	126	6	4.48%	4.76%	unstrandedTCGA
HTSeq_HISAT2	(3,Inf)	2815	126	10	4.48%	7.94%	unstrandedTCGA
HTSeq_STAR	(3,Inf)	2815	126	11	4.48%	8.73%	unstrandedTCGA
HTSeq_Subread	(3,Inf)	2815	126	8	4.48%	6.35%	unstrandedTCGA
Kallisto	(3,Inf)	2815	126	1	4.48%	0.79%	unstrandedTCGA
Salmon	(3,Inf)	2815	126	1	4.48%	0.79%	unstrandedTCGA
featureCounts_HISAT2	(0,1]	2413	285	16	11.81%	5.61%	reverseTCGA
featureCounts_STAR	(0,1]	2413	285	17	11.81%	5.96%	reverseTCGA
featureCounts_Subread	(0,1]	2413	285	16	11.81%	5.61%	reverseTCGA
HTSeq_HISAT2	(0,1]	2413	285	17	11.81%	5.96%	reverseTCGA
HTSeq_STAR	(0,1]	2413	285	18	11.81%	6.32%	reverseTCGA
HTSeq_Subread	(0,1]	2413	285	18	11.81%	6.32%	reverseTCGA
Kallisto	(0,1]	2413	285	4	11.81%	1.40%	reverseTCGA
Salmon	(0,1]	2413	285	3	11.81%	1.05%	reverseTCGA
featureCounts_HISAT2	(1,2]	5610	138	2	2.46%	1.45%	reverseTCGA
featureCounts_STAR	(1,2]	5610	138	2	2.46%	1.45%	reverseTCGA
featureCounts_Subread	(1,2]	5610	138	2	2.46%	1.45%	reverseTCGA
HTSeq_HISAT2	(1,2]	5610	138	3	2.46%	2.17%	reverseTCGA
HTSeq_STAR	(1,2]	5610	138	3	2.46%	2.17%	reverseTCGA
HTSeq_Subread	(1,2]	5610	138	3	2.46%	2.17%	reverseTCGA
Kallisto	(1,2]	5610	138	0	2.46%	0.00%	reverseTCGA
Salmon	(1,2]	5610	138	1	2.46%	0.72%	reverseTCGA

featureCounts_HISAT2	(2,3]	3330	140	4	4.20%	2.86%	reverseTCGA
featureCounts_STAR	(2,3]	3330	140	1	4.20%	0.71%	reverseTCGA
featureCounts_Subread	(2,3]	3330	140	4	4.20%	2.86%	reverseTCGA
HTSeq_HISAT2	(2,3]	3330	140	4	4.20%	2.86%	reverseTCGA
HTSeq_STAR	(2,3]	3330	140	1	4.20%	0.71%	reverseTCGA
HTSeq_Subread	(2,3]	3330	140	5	4.20%	3.57%	reverseTCGA
Kallisto	(2,3]	3330	140	0	4.20%	0.00%	reverseTCGA
Salmon	(2,3]	3330	140	0	4.20%	0.00%	reverseTCGA
featureCounts_HISAT2	(3,Inf)	2815	166	6	5.90%	3.61%	reverseTCGA
featureCounts_STAR	(3,Inf)	2815	166	7	5.90%	4.22%	reverseTCGA
featureCounts_Subread	(3,Inf)	2815	166	7	5.90%	4.22%	reverseTCGA
HTSeq_HISAT2	(3,Inf)	2815	166	6	5.90%	3.61%	reverseTCGA
HTSeq_STAR	(3,Inf)	2815	166	7	5.90%	4.22%	reverseTCGA
HTSeq_Subread	(3,Inf)	2815	166	8	5.90%	4.82%	reverseTCGA
Kallisto	(3,Inf)	2815	166	0	5.90%	0.00%	reverseTCGA
Salmon	(3,Inf)	2815	166	0	5.90%	0.00%	reverseTCGA
featureCounts_HISAT2	(0,1]	2413	127	12	5.26%	9.45%	reversePRJEB11797
featureCounts_STAR	(0,1]	2413	127	13	5.26%	10.24%	reversePRJEB11797
featureCounts_Subread	(0,1]	2413	127	13	5.26%	10.24%	reversePRJEB11797
HTSeq_HISAT2	(0,1]	2413	127	13	5.26%	10.24%	reversePRJEB11797
HTSeq_STAR	(0,1]	2413	127	13	5.26%	10.24%	reversePRJEB11797
HTSeq_Subread	(0,1]	2413	127	13	5.26%	10.24%	reversePRJEB11797
Kallisto	(0,1]	2413	127	0	5.26%	0.00%	reversePRJEB11797
Salmon	(0,1]	2413	127	0	5.26%	0.00%	reversePRJEB11797
featureCounts_HISAT2	(1,2]	5610	101	0	1.80%	0.00%	reversePRJEB11797
featureCounts_STAR	(1,2]	5610	101	0	1.80%	0.00%	reversePRJEB11797
featureCounts_Subread	(1,2]	5610	101	0	1.80%	0.00%	reversePRJEB11797
HTSeq_HISAT2	(1,2]	5610	101	1	1.80%	0.99%	reversePRJEB11797
HTSeq_STAR	(1,2]	5610	101	1	1.80%	0.99%	reversePRJEB11797
HTSeq_Subread	(1,2]	5610	101	0	1.80%	0.00%	reversePRJEB11797
Kallisto	(1,2]	5610	101	0	1.80%	0.00%	reversePRJEB11797
Salmon	(1,2]	5610	101	1	1.80%	0.99%	reversePRJEB11797
featureCounts_HISAT2	(2,3]	3330	118	2	3.54%	1.69%	reversePRJEB11797
featureCounts_STAR	(2,3]	3330	118	1	3.54%	0.85%	reversePRJEB11797
featureCounts_Subread	(2,3]	3330	118	2	3.54%	1.69%	reversePRJEB11797
HTSeq_HISAT2	(2,3]	3330	118	2	3.54%	1.69%	reversePRJEB11797
HTSeq_STAR	(2,3]	3330	118	1	3.54%	0.85%	reversePRJEB11797
HTSeq_Subread	(2,3]	3330	118	2	3.54%	1.69%	reversePRJEB11797
Kallisto	(2,3]	3330	118	0	3.54%	0.00%	reversePRJEB11797
Salmon	(2,3]	3330	118	0	3.54%	0.00%	reversePRJEB11797
featureCounts_HISAT2	(3,Inf)	2815	118	5	4.19%	4.24%	reversePRJEB11797
featureCounts_STAR	(3,Inf)	2815	118	3	4.19%	2.54%	reversePRJEB11797
featureCounts_Subread	(3,Inf)	2815	118	4	4.19%	3.39%	reversePRJEB11797
HTSeq_HISAT2	(3,Inf)	2815	118	6	4.19%	5.08%	reversePRJEB11797
HTSeq_STAR	(3,Inf)	2815	118	3	4.19%	2.54%	reversePRJEB11797
HTSeq_Subread	(3,Inf)	2815	118	7	4.19%	5.93%	reversePRJEB11797
Kallisto	(3,Inf)	2815	118	1	4.19%	0.85%	reversePRJEB11797
Salmon	(3,Inf)	2815	118	1	4.19%	0.85%	reversePRJEB11797

Additional file 15. Feature (sequence uniqueness) of discordant lncRNAs

methods	type	total	expressed	discordant	expressedPCT	discordantPCT	library
featureCounts_HISAT2	(0,0.2]	350	30	23	8.57%	76.67%	unstrandedTCGA
featureCounts_STAR	(0,0.2]	350	30	23	8.57%	76.67%	unstrandedTCGA
featureCounts_Subread	(0,0.2]	350	30	19	8.57%	63.33%	unstrandedTCGA
HTSeq_HISAT2	(0,0.2]	350	30	23	8.57%	76.67%	unstrandedTCGA
HTSeq_STAR	(0,0.2]	350	30	23	8.57%	76.67%	unstrandedTCGA
HTSeq_Subread	(0,0.2]	350	30	19	8.57%	63.33%	unstrandedTCGA
Kallisto	(0,0.2]	350	30	1	8.57%	3.33%	unstrandedTCGA
Salmon	(0,0.2]	350	30	1	8.57%	3.33%	unstrandedTCGA
featureCounts_HISAT2	(0.2,0.4]	149	10	1	6.71%	10.00%	unstrandedTCGA
featureCounts_STAR	(0.2,0.4]	149	10	0	6.71%	0.00%	unstrandedTCGA
featureCounts_Subread	(0.2,0.4]	149	10	1	6.71%	10.00%	unstrandedTCGA
HTSeq_HISAT2	(0.2,0.4]	149	10	2	6.71%	20.00%	unstrandedTCGA
HTSeq_STAR	(0.2,0.4]	149	10	1	6.71%	10.00%	unstrandedTCGA
HTSeq_Subread	(0.2,0.4]	149	10	2	6.71%	20.00%	unstrandedTCGA
Kallisto	(0.2,0.4]	149	10	0	6.71%	0.00%	unstrandedTCGA
Salmon	(0.2,0.4]	149	10	0	6.71%	0.00%	unstrandedTCGA
featureCounts_HISAT2	(0.4,0.6]	157	14	0	8.92%	0.00%	unstrandedTCGA
featureCounts_STAR	(0.4,0.6]	157	14	0	8.92%	0.00%	unstrandedTCGA
featureCounts_Subread	(0.4,0.6]	157	14	0	8.92%	0.00%	unstrandedTCGA
HTSeq_HISAT2	(0.4,0.6]	157	14	1	8.92%	7.14%	unstrandedTCGA
HTSeq_STAR	(0.4,0.6]	157	14	1	8.92%	7.14%	unstrandedTCGA
HTSeq_Subread	(0.4,0.6]	157	14	1	8.92%	7.14%	unstrandedTCGA
Kallisto	(0.4,0.6]	157	14	0	8.92%	0.00%	unstrandedTCGA
Salmon	(0.4,0.6]	157	14	0	8.92%	0.00%	unstrandedTCGA
featureCounts_HISAT2	(0.6,0.8]	451	28	5	6.21%	17.86%	unstrandedTCGA
featureCounts_STAR	(0.6,0.8]	451	28	4	6.21%	14.29%	unstrandedTCGA
featureCounts_Subread	(0.6,0.8]	451	28	4	6.21%	14.29%	unstrandedTCGA
HTSeq_HISAT2	(0.6,0.8]	451	28	5	6.21%	17.86%	unstrandedTCGA
HTSeq_STAR	(0.6,0.8]	451	28	6	6.21%	21.43%	unstrandedTCGA
HTSeq_Subread	(0.6,0.8]	451	28	5	6.21%	17.86%	unstrandedTCGA
Kallisto	(0.6,0.8]	451	28	0	6.21%	0.00%	unstrandedTCGA
Salmon	(0.6,0.8]	451	28	0	6.21%	0.00%	unstrandedTCGA
featureCounts_HISAT2	(0.8,1]	13061	582	83	4.46%	14.26%	unstrandedTCGA
featureCounts_STAR	(0.8,1]	13061	582	84	4.46%	14.43%	unstrandedTCGA
featureCounts_Subread	(0.8,1]	13061	582	82	4.46%	14.09%	unstrandedTCGA
HTSeq_HISAT2	(0.8,1]	13061	582	104	4.46%	17.87%	unstrandedTCGA
HTSeq_STAR	(0.8,1]	13061	582	105	4.46%	18.04%	unstrandedTCGA
HTSeq_Subread	(0.8,1]	13061	582	103	4.46%	17.70%	unstrandedTCGA
Kallisto	(0.8,1]	13061	582	3	4.46%	0.52%	unstrandedTCGA
Salmon	(0.8,1]	13061	582	3	4.46%	0.52%	unstrandedTCGA
featureCounts_HISAT2	(0,0.2]	350	37	25	10.57%	67.57%	reverseTCGA
featureCounts_STAR	(0,0.2]	350	37	25	10.57%	67.57%	reverseTCGA
featureCounts_Subread	(0,0.2]	350	37	24	10.57%	64.86%	reverseTCGA
HTSeq_HISAT2	(0,0.2]	350	37	25	10.57%	67.57%	reverseTCGA
HTSeq_STAR	(0,0.2]	350	37	25	10.57%	67.57%	reverseTCGA
HTSeq_Subread	(0,0.2]	350	37	26	10.57%	70.27%	reverseTCGA
Kallisto	(0,0.2]	350	37	4	10.57%	10.81%	reverseTCGA
Salmon	(0,0.2]	350	37	3	10.57%	8.11%	reverseTCGA
featureCounts_HISAT2	(0.2,0.4]	149	22	2	14.77%	9.09%	reverseTCGA
featureCounts_STAR	(0.2,0.4]	149	22	1	14.77%	4.55%	reverseTCGA
featureCounts_Subread	(0.2,0.4]	149	22	1	14.77%	4.55%	reverseTCGA
HTSeq_HISAT2	(0.2,0.4]	149	22	4	14.77%	18.18%	reverseTCGA
HTSeq_STAR	(0.2,0.4]	149	22	3	14.77%	13.64%	reverseTCGA
HTSeq_Subread	(0.2,0.4]	149	22	3	14.77%	13.64%	reverseTCGA
Kallisto	(0.2,0.4]	149	22	0	14.77%	0.00%	reverseTCGA
Salmon	(0.2,0.4]	149	22	0	14.77%	0.00%	reverseTCGA

featureCounts_HISAT2	(0.4,0.6]	157	20	1	12.74%	5.00%	reverseTCGA
featureCounts_STAR	(0.4,0.6]	157	20	0	12.74%	0.00%	reverseTCGA
featureCounts_Subread	(0.4,0.6]	157	20	1	12.74%	5.00%	reverseTCGA
HTSeq_HISAT2	(0.4,0.6]	157	20	1	12.74%	5.00%	reverseTCGA
HTSeq_STAR	(0.4,0.6]	157	20	0	12.74%	0.00%	reverseTCGA
HTSeq_Subread	(0.4,0.6]	157	20	1	12.74%	5.00%	reverseTCGA
Kallisto	(0.4,0.6]	157	20	0	12.74%	0.00%	reverseTCGA
Salmon	(0.4,0.6]	157	20	0	12.74%	0.00%	reverseTCGA
featureCounts_HISAT2	(0.6,0.8]	451	37	0	8.20%	0.00%	reverseTCGA
featureCounts_STAR	(0.6,0.8]	451	37	0	8.20%	0.00%	reverseTCGA
featureCounts_Subread	(0.6,0.8]	451	37	2	8.20%	5.41%	reverseTCGA
HTSeq_HISAT2	(0.6,0.8]	451	37	0	8.20%	0.00%	reverseTCGA
HTSeq_STAR	(0.6,0.8]	451	37	0	8.20%	0.00%	reverseTCGA
HTSeq_Subread	(0.6,0.8]	451	37	2	8.20%	5.41%	reverseTCGA
Kallisto	(0.6,0.8]	451	37	0	8.20%	0.00%	reverseTCGA
Salmon	(0.6,0.8]	451	37	0	8.20%	0.00%	reverseTCGA
featureCounts_HISAT2	(0.8,1]	13061	613	0	4.69%	0.00%	reverseTCGA
featureCounts_STAR	(0.8,1]	13061	613	1	4.69%	0.16%	reverseTCGA
featureCounts_Subread	(0.8,1]	13061	613	1	4.69%	0.16%	reverseTCGA
HTSeq_HISAT2	(0.8,1]	13061	613	0	4.69%	0.00%	reverseTCGA
HTSeq_STAR	(0.8,1]	13061	613	1	4.69%	0.16%	reverseTCGA
HTSeq_Subread	(0.8,1]	13061	613	2	4.69%	0.33%	reverseTCGA
Kallisto	(0.8,1]	13061	613	0	4.69%	0.00%	reverseTCGA
Salmon	(0.8,1]	13061	613	1	4.69%	0.16%	reverseTCGA
featureCounts_HISAT2	(0,0.2]	350	24	17	6.86%	70.83%	reversePRJEB11797
featureCounts_STAR	(0,0.2]	350	24	16	6.86%	66.67%	reversePRJEB11797
featureCounts_Subread	(0,0.2]	350	24	17	6.86%	70.83%	reversePRJEB11797
HTSeq_HISAT2	(0,0.2]	350	24	18	6.86%	75.00%	reversePRJEB11797
HTSeq_STAR	(0,0.2]	350	24	15	6.86%	62.50%	reversePRJEB11797
HTSeq_Subread	(0,0.2]	350	24	19	6.86%	79.17%	reversePRJEB11797
Kallisto	(0,0.2]	350	24	1	6.86%	4.17%	reversePRJEB11797
Salmon	(0,0.2]	350	24	1	6.86%	4.17%	reversePRJEB11797
featureCounts_HISAT2	(0.2,0.4]	149	15	1	10.07%	6.67%	reversePRJEB11797
featureCounts_STAR	(0.2,0.4]	149	15	1	10.07%	6.67%	reversePRJEB11797
featureCounts_Subread	(0.2,0.4]	149	15	1	10.07%	6.67%	reversePRJEB11797
HTSeq_HISAT2	(0.2,0.4]	149	15	2	10.07%	13.33%	reversePRJEB11797
HTSeq_STAR	(0.2,0.4]	149	15	2	10.07%	13.33%	reversePRJEB11797
HTSeq_Subread	(0.2,0.4]	149	15	1	10.07%	6.67%	reversePRJEB11797
Kallisto	(0.2,0.4]	149	15	0	10.07%	0.00%	reversePRJEB11797
Salmon	(0.2,0.4]	149	15	0	10.07%	0.00%	reversePRJEB11797
featureCounts_HISAT2	(0.4,0.6]	157	14	1	8.92%	7.14%	reversePRJEB11797
featureCounts_STAR	(0.4,0.6]	157	14	0	8.92%	0.00%	reversePRJEB11797
featureCounts_Subread	(0.4,0.6]	157	14	1	8.92%	7.14%	reversePRJEB11797
HTSeq_HISAT2	(0.4,0.6]	157	14	1	8.92%	7.14%	reversePRJEB11797
HTSeq_STAR	(0.4,0.6]	157	14	0	8.92%	0.00%	reversePRJEB11797
HTSeq_Subread	(0.4,0.6]	157	14	1	8.92%	7.14%	reversePRJEB11797
Kallisto	(0.4,0.6]	157	14	0	8.92%	0.00%	reversePRJEB11797
Salmon	(0.4,0.6]	157	14	0	8.92%	0.00%	reversePRJEB11797
featureCounts_HISAT2	(0.6,0.8]	451	21	0	4.66%	0.00%	reversePRJEB11797
featureCounts_STAR	(0.6,0.8]	451	21	0	4.66%	0.00%	reversePRJEB11797
featureCounts_Subread	(0.6,0.8]	451	21	0	4.66%	0.00%	reversePRJEB11797
HTSeq_HISAT2	(0.6,0.8]	451	21	0	4.66%	0.00%	reversePRJEB11797
HTSeq_STAR	(0.6,0.8]	451	21	0	4.66%	0.00%	reversePRJEB11797
HTSeq_Subread	(0.6,0.8]	451	21	0	4.66%	0.00%	reversePRJEB11797
Kallisto	(0.6,0.8]	451	21	0	4.66%	0.00%	reversePRJEB11797
Salmon	(0.6,0.8]	451	21	0	4.66%	0.00%	reversePRJEB11797
featureCounts_HISAT2	(0.8,1]	13061	390	0	2.99%	0.00%	reversePRJEB11797
featureCounts_STAR	(0.8,1]	13061	390	0	2.99%	0.00%	reversePRJEB11797
featureCounts_Subread	(0.8,1]	13061	390	0	2.99%	0.00%	reversePRJEB11797
HTSeq_HISAT2	(0.8,1]	13061	390	1	2.99%	0.26%	reversePRJEB11797
HTSeq_STAR	(0.8,1]	13061	390	1	2.99%	0.26%	reversePRJEB11797
HTSeq_Subread	(0.8,1]	13061	390	1	2.99%	0.26%	reversePRJEB11797
Kallisto	(0.8,1]	13061	390	0	2.99%	0.00%	reversePRJEB11797
Salmon	(0.8,1]	13061	390	1	2.99%	0.26%	reversePRJEB11797

Additional file 16. Overall feature breakdown of GENCODE, expressed, and discordant lncRNAs

			Number of transcripts					Transcript length				Number of exons				Fraction of uniqueness				
			(0,1]	(1,2]	(2,3]	(3,10]	(10,Inf)	(0,400]	(400,1k]	(1k,5k]	(5k,Inf)	(0,1]	(1,2]	(2,3]	(3,Inf)	(0,0.2]	(0.2,0.4]	(0.4,0.6]	(0.6,0.8]	(0.8,1]
GENCODE		antisense	4033	733	298	368	89	767	3396	1332	26	697	2468	1324	1032	67	33	38	159	5224
		lincRNA	5607	1008	397	426	61	953	4468	2014	64	1241	2642	1908	1708	241	100	107	231	6820
		other	1063	57	13	13	2	199	660	263	26	475	500	98	75	42	16	12	61	1017
unstranded	expressed	antisense	176	58	41	89	26	16	217	157	0	75	153	87	75	2	3	4	15	366
		lincRNA	98	31	21	55	22	10	95	116	6	81	44	52	50	19	5	10	12	181
		other	38	5	2	2	0	3	19	22	3	29	14	3	1	9	2	0	1	35
	discordant	antisense	75	16	5	5	0	13	71	17	0	18	74	5	4	2	0	1	5	93
		lincRNA	14	3	0	5	2	1	15	8	0	8	5	4	7	13	1	0	1	9
		other	13	0	0	0	0	2	6	2	3	8	5	0	0	9	1	0	0	3
reverseTCGA	expressed	antisense	75	30	25	92	28	4	129	115	2	55	42	71	82	2	3	5	12	228
		lincRNA	121	31	31	75	29	12	126	139	10	99	43	65	80	24	13	13	19	218
		other	177	8	3	4	0	14	95	77	6	131	53	4	4	11	6	2	6	167
	discordant	antisense	1	0	0	3	0	0	3	1	0	1	1	0	2	2	0	0	0	2
		lincRNA	11	0	2	9	1	3	11	9	0	9	0	6	8	15	4	1	2	1
		other	11	0	0	0	0	3	4	2	2	8	3	0	0	10	1	0	0	0
reversePRJEB11797	expressed	antisense	64	28	25	65	19	4	104	91	2	37	49	59	56	1	1	5	10	184
		lincRNA	70	32	24	61	23	11	93	101	5	52	42	57	59	17	8	9	11	165
		other	43	6	1	3	0	4	24	22	3	38	10	2	3	6	6	0	0	41
	discordant	antisense	1	0	0	1	0	0	2	0	0	0	1	0	1	1	0	0	0	1
		lincRNA	7	0	2	6	1	2	8	6	0	7	0	2	7	12	1	1	0	2
		other	7	0	0	0	0	2	2	1	2	6	1	0	0	6	1	0	0	0

Additional file 17. Reads mapping of discordant lncRNAs

Gene	Chr	Start	End	Strand	Type	Properly aligned	Poorly aligned	Misaligned
ENSG00000232940.5	chr6	33249534	33254989	+	antisense	46.46%	5.45%	48.09%
ENSG00000242539.2	chr3	179396961	179399191	+	antisense	5.09%	0.45%	94.46%
ENSG00000253174.2	chr8	41540381	41545044	-	antisense	14.36%	1.34%	84.30%
ENSG00000254452.1	chr11	66276779	66277492	-	antisense	6.37%	4.03%	89.60%
ENSG00000254721.1	chr11	70206291	70207390	-	antisense	13.23%	1.21%	85.56%
ENSG00000256690.1	chr11	62832234	62834043	+	antisense	91.31%	8.59%	0.10%
ENSG00000258504.2	chr14	100291117	100294656	+	lincRNA	91.44%	8.44%	0.12%
ENSG00000259049.1	chr14	52775237	52777740	+	antisense	91.68%	7.99%	0.33%
ENSG00000259884.1	chr12	52058459	52059503	-	lincRNA	89.69%	10.15%	0.15%
ENSG00000261822.1	chr15	42567031	42569994	-	antisense	90.77%	7.95%	1.28%
ENSG00000266642.2	chr17	28897738	28899402	+	antisense	91.50%	8.36%	0.14%
ENSG00000269604.1	chr19	4791745	4795559	-	antisense	82.61%	17.21%	0.17%
ENSG00000272758.5	chr3	122416207	122443180	+	antisense	90.48%	8.99%	0.53%
ENSG00000275496.4	chr21	6228966	6267317	-	lincRNA	0.01%	99.87%	0.13%
ENSG00000276077.4	chr21	7430659	7469007	+	lincRNA	0.01%	99.84%	0.16%

The genome alignment of lncRNA reads was checked using bam files generated by Subread. A total of 15 lncRNAs that are quantified not accurately by HTSeq and featureCounts in the Polyester-simulated unstranded sample cohort were examined.
Properly aligned: Reads were aligned to the correct genome location with mapping quality >=20
Poorly aligned: Reads were aligned to the correct genome location with mapping quality <20
Misaligned: Reads were not aligned to the correct genome location.
