# Supplementary material for: Benchmark of long non-coding RNA quantification for RNA sequencing of cancer samples
Source: Gigascience. 2019 Dec 6;8(12):giz145. doi: 10.1093/gigascience/giz145 (PMC6897288; doi:10.1093/gigascience/giz145)
Supplement: giz145_Response_to_Reviewer_Comments_Original_Submission [file giz145_response_to_reviewer_comments_original_submission.pdf]

Author's Response To Reviewer Comments

Close

We thank all the reviewers for their constructive comments. We have addressed or clarified all the points raised by the reviewers in the revised manuscript. Our point-to-point response are below. The manuscript has been revised. We hope to receive favorable consideration from the editor and reviewers.

Reviewer #1: This manuscript described a careful benchmarking design to evaluate the lncRNA quantification performance of several pipelines for RNA-Seq data of cancer samples. The authors showed that methods that only utilize unique reads performed badly for lincRNA quantification. This result is important and timely since HTSeq is the current default workflow for TCGA data and it is crucial for the cancer research community to understand its strengths and weaknesses.

I strongly recommend the authors to address the 3 major comments below, which can significantly improve their manuscript:

1) The conclusion that "pseudoalignment methods outperform alignment-based methods" is inappropriate.

As the authors mentioned, the plausible reason that Kallisto and Salmon outperforms HTSeq and featureCounts is Kallisto and Salmon models multi-mapping reads better. In fact, RSEM is also an alignment-based method and can allocate multi-mapping reads appropriately. I suggest the authors to change the conclusion to "methods utilizing multi-mapping reads outperforms methods that only use unique reads".

Thanks for the suggestion. We have revised the abstract and the main text according to the suggestion and the new benchmark results, stating that "Pseudoalignment methods and RSEM outperform HTSeq and featureCounts for lncRNA quantification at both sample- and gene-level comparison".

In the revision, we have added RSEM in the benchmark (Figure 3), and observed similar performance with pseudoalignment methods Kallisto and Salmon, all of them performed better than HTSeq and featureCounts.

We refer to HTSeq and featureCounts as "alignment-based gene quantification methods" to differentiate from RSEM, which is also alignment-based, but focuses on transcript quantification. We didn't describe HTSeq and featureCounts as "methods that only use unique reads", since there are options in their parameter settings regarding how to deal with multi-mapping reads in quantification. We have tested these different parameters and found that fine-tuning these parameters didn't improve performance (results not shown).

2) Please include RSEM in the benchmarking.

RSEM is also an alignment-based method. Please also benchmark RSEM + STAR, RSEM + HISAT2, and RSEM + Subread.

We have included RSEM in the benchmarking in the revised manuscript (Figure 3 and the highlighted main text). RSEM integrates bowtie as the default aligner to map the reads to the transcriptome, which is also the workflow that we evaluated. We didn't include the other aligners, since 1) the default bowtie option is recommended by the RSEM author, and 2) HISAT2 and Subread were developed for genome mapping. The new benchmarking results show that RSEM performs as good as pseudoalignment methods.

3) Please consider to conduct some benchmark experiments using real data.

The benchmark conducted in this manuscript is based on simulated data sets using the RSEM simulator. Since both Kallisto and Salmon use graphical models that are highly similar to RSEM's model, there might be a bias in the benchmarking. Thus, it will be great if we can see some benchmarking results purely based on real datasets.

We agree with the reviewer that using only RSEM for simulating data is not sufficient. Thus, we have evaluated the methods on another dataset generated using a different simulation method, Polyester, and reached similar conclusions.

However, there is no good “real data” for evaluating RNA-Seq experiments. Some studies generated data from other platforms like RT-PCR and compare with RNA-Seq expression. However, the discrepancy between RT-PCR and RNA-Seq expression measures may be large and even greater than the difference between the RNA-Seq analysis tools themselves. In addition, it would require extensive laboratory work to generate such data, which is out of the scope of the current study. We added this concern in the discussion as part of the limitation of this study.

Nevertheless, in the current study, we have used two different simulation tools to generate both un-stranded and strand-specific RNA-Seq data from different sources, compared the performance of the most widely used tools in RNA-Seq analysis, and also evaluated the effect of the choices of different transcriptome annotations and read aligners. Thus, this study is of great value for the research community to choose suitable tools in RNA-Seq analysis, and also provides insights for more comprehensive benchmarking studies in the future.

Reviewer #2: Major comments

The methods chosen for benchmarking do not include the EM-based methods to estimate gene expressions: e..g RSEM or IsoEM. RSEM was only used to generate gold standard gene expressions. This might explain low accuracy of mapping based gene quantification methods.

We agree with the reviewer that using only RSEM for simulating data may not be sufficient. Thus, we have re-evaluated the methods on another dataset using a different simulation method, Polyester.

We have also added RSEM in the benchmark (Figure 3), and observed similar performance with pseudoalignment methods Kallisto and Salmon, all of them performed better than other alignment-based gene quantification methods.

The manuscripts also lack details of which mapping methods provided the best results. Was is the same method for both htseq and featureCount

We have added the comparison between mapping tools, as well as HTSeq and featureCounts in the discussion part. featureCounts performs slightly better than HTSeq, and also takes much shorter CPU time. Subread performs slightly better than the other two aligners for unstranded samples.

It would be beneficial to explore the effect of parameters (of the mapping tools) on the accuracy of quantification. It was suggested here that the tuned parameters usually provide better results: Baruzzo, Giacomo, et al. "Simulation-based comprehensive benchmarking of RNA-seq aligners." Nature methods 14.2 (2017): 135.

We thank the reviewer to raise this point that fine-tuning with the mapping parameters may affect downstream expression quantification. However, in the "Simulation-based comprehensive benchmarking of RNA-seq aligners" paper the authors stated that “the algorithm that benefits most dramatically from parameter tuning is TopHat2, while CLC, Novoalign, GSNAP, MapSplice2, and STAR perform the best with defaults.” Since we have included STAR in the evaluation with the default setting, the inferior performance of the STAR workflow is not due to the mapping step. As is explained in the discussion, the inferior performance of alignment-based tools are more likely due to the imperfect handling of ambiguous reads by HTSeq and featureCounts.

On a similar note, it would be interesting to explore what is causing low performance of mapping-based tool. Is it an incorrect alignment? To quantify this, authors can use .bam files with true alignment where reads are "mapped" to the position from where they were simulated. It will be interesting to compare

this across un stranded and reverse standard libraries.

As suggested, we have checked the bam files to see the alignment of the reads. We have checked the top 15 lncRNAs that are not quantified accurately by HTSeq and featureCounts. We have added a supplementary table (Additional file 17) summarizing the results. These genes are either not properly aligned to the genome, or even if they are aligned, due to the annotation overlapping with other genes, HTSeq and featureCounts cannot determine where the reads should be assigned. We have added this to the discussion.

The study does not explore the important sequencing features including read length and throughput (number of reads). It would be beneficial to add this at least for several datasets and report how the accuracy of gene quantification depends on these parameters.

Authors use RSEM to generate which will be used for simulating the reads. Why RSEM was chosen? Also, do the reads contain sequencing errors and SNPs? Were the reads paired-end reads? If so what was the fragment length distribution?

More details regarding read length and throughput were provided in the “Data Descriptome” part. The read length in the dataset we generated ranges from 50 bp (TCGA samples) to 100bp (the PRJEB11797 dataset), and the conclusion holds true for both occasions.

Originally we choose RSEM for simulating the reads since it uses the model information and quantification results of real samples. Regarding sequencing errors, RSEM ‘rsem-simulate-reads learns a parameter called theta0 from the real data, which determines the fraction of reads that are coming from background “noise” and is used in the simulation. The reads are paired-end reads. The fragment length distribution is 178±60 bp (mean±sd) for TCGA samples and 155±51 bp for the other dataset. The information has been added in the revised manuscript.

We have now included another simulator, Polyester, to generate additional datasets. The conclusion still holds true for the new dataset.

The main limitation of the study is that only simulated reads were used. Authors need to mention the limitation of the simulated data as such data usually the reality. This was recently highlighted in a recent paper about benchmarking: Mangul, Serghei, et al. "Systematic benchmarking of omics computational tools." Nature communications 10 (2019).

We agree with the reviewer that using only simulating data may not be sufficient. We addressed this concern in the discussion.

It is not clear what authors mean by sample-level and gene-level. More details needs to be provided.

As suggested by the reviewer, we have provided more details in the methods part.

Discussion section lack discussion on which alignment was the best. And in general what was causing low performance of mapping based methods. If it will be determined to be mapping quality (see point point 4) when such discussion needs to be added. Or maybe it is due to the fact that both mapping-based methods were non-EM. and RSEM might show much better results.

As suggested by the reviewer, we have added the comparison between mapping tools, as well as HTSeq and featureCounts in the discussion part.

We have also added RSEM in the benchmark with new dataset (not generated by RSEM), and, as predicted by the reviewer, RSEM indeed shows much better results than HTSeq and featureCounts, and it is comparable to pseudoalignment methods. We agree with the reviewer that the inferior performance of HTSeq and featureCounts may be due to their non-EM nature. We have added this point in the discussion.

The formal comparison of max RAM and CPU time for each method needs to be added into a separate figure. The running time for mapping based methods needs to be split into method itself and mapping.

As suggested by the reviewer, we have added the RAM and CPU time for each method into a seperate figure (Figure 7). The measurements for the mapping tools and the methods are displayed separately.

In addition to used measured it would be beneficial to use: error fraction (EF) and median percent error (MPE) measures as was suggested here Nicolae, Marius, et al. "Estimation of alternative splicing isoform frequencies from RNA-Seq data." Algorithms for molecular biology 6.1 (2011): 9. Those measures are particularly sensitive for low expressed genes.

Thanks for the suggestion. We have added the MPE and EF measures (Figure 2, Figure 3 and the main text) in the comparison of the methods. These additional measurements are in agreement with other measures, supporting the conclusion that pseudoaliment methods outperform HTSeq and featureCounts.

Minor comments

Page 2. Line 50. I think the authors mean that the majority of TCGA samples were prepared

We thank the reviewer for pointing out the typo. We have revised it in the manuscript.

Page3. Line 7. Effect of incomplete annotation is known and there is extensive literature on this. For example Mangul, Serghei, et al. "Improved transcriptome quantification and reconstruction from RNA-Seq reads using partial annotations." In silico biology 11.5, 6 (2011): 251-261.

Thanks for pointing this out. Our study validated previous findings and also extended the recommendation of using full annotations to a broader scenario, including both traditional alignment-based methods as well as the pesudoalignment methods.

P 3. Line 57 Is it not clear what the three pipelines are referring to

The “three pipelines” means three read aligners (STAR, HISAT2, and Subread) in combination with HTSeq or featureCounts. We have added additional description to clarify it in the main text.

Reviewer #3:
General comments
The described work is devoted to the comparison of different methods for quantifying lncRNAs using RNA-Seq. Since quantification pipelines selected for the benchmark are widely used and appear to be the most popular among all the available tools, I consider this work to be valuable for the community. Authors use publicly available datasets, provide exact versions and command lines for all tools and also upload supplementary scripts to public repository, which make the research completely reproducible. Benchmarking methods are transparent and provide various insights on different quantification tools and sequencing protocols. Metrics used for the comparison seem to be relevant and enough to make conclusions stated in the paper.
The manuscript itself is well-structured and easy to follow.
I summarize a few minor questions below.

Minor comments
- Based on my experience, RSEM simulator is the best tool for generating RNA-Seq reads. However, I have a suspicion that introduced sequencing errors may somehow differ from the ones in real Illumina reads. Although benchmarking and testing a third-party simulator may be out of scope of this work, quality of simulated data may be essential, especially for alignment-based methods. Did you perform any checks, e.g. compared mapping rates, error profiles and nucleotide substitution frequency matrices for real and simulated data? Unfortunately, I couldn’t find such information in the original RSEM paper [1].

We checked the mapping rates between simulated data and real data, and found no significant difference. We also applied the methods on real datasets and compared the expression measures obtained from real data and simulated data for the same sample. Expression measures from real data and simulated data are highly concordant for all the methods evaluated (median and mean Spearman’s correlation above 0.98 in sample-wise comparison). Therefore, although simulated data may be different from the real data, the difference doesn’t influence the gene expression measurements.

More importantly, we agree with the reviewer that using only RSEM for simulating data may not be sufficient. Thus, we have evaluated the methods on another dataset using a different simulation method,

Polyester, and reached similar conclusions.

- Authors compare different methods with ground truth using multiple metrics based on FPKM values. Some studies, however, recommend to use TPM values for quantification [2]. It would be interesting to know whether using TPM instead of FPKM changes the results in any way (my guess that it won't result in any significant changes in this particular case).

We have actually used both TPM and FPKM values for evaluation, and conclusion holds true for both occasions. For simplicity reasons we showed the results for FPKM values.

- In "Availability of source code and requirements" section. I'm not sure that the benchmarking pipeline is "platform independent" as stated, since the repository contains Linux sh scripts and binaries. I would also exactly point out programming languages used in the project — Shell, python, perl, R.

Thanks for the suggestion. We have changed the "platform independent" statement and also stated clearly the programming languages used in the project.

Close
