# Supplementary material for: Benchmark of long non-coding RNA quantification for RNA sequencing of cancer samples
Source: Gigascience. 2019 Dec 6;8(12):giz145. doi: 10.1093/gigascience/giz145 (PMC6897288; doi:10.1093/gigascience/giz145)
Supplement: giz145_Response_to_Reviewer_Comments_Revision_1 [file giz145_response_to_reviewer_comments_revision_1.pdf]

Author's Response To Reviewer Comments

Close

We thank the reviewers for the constructive comments. We have addressed or clarified all the points raised by the reviewers in the revised manuscript. Our point-to-point response are below. The manuscript has been revised. We hope to receive favorable consideration from the editor and reviewers.

1. The answers prepared by the authors were not properly formatted. It lacks numbering and having the added text or figure directly in the answer will be extremely useful

Reply: We apologize for the inconvenience. In this revision the answers are labeled by number. The revised texts are highlighted in the manuscript and we also include them in the replies.

2. RSEM is in the wrong category. Since it requires the mapping it should be in a similar category as other Quantification tools

Reply: Thanks for the suggestion. We have modified Figure 7 and put RSEM in the category of quantification tools.

3. It will be useful to plot CPU time vs accuracy to emphasise the ability of pseudo alignment methods to deliver similar results in a short amount of time

Reply: Thanks for the suggestion. We have also added the accuracy of each tool (measured by Spearman's correlation) in Figure 7.

4. Similar methods as RSEM need to be cited. For example IsoEM

Reply: We added similar methods using expectation-maximization algorithm such as IsoEM in the discussion:

"Other methods using expectation-maximization algorithm such as IsoEM {\cite{nicolae2011}} might achieve similar accuracy as pseudoalignment methods and RSEM."

5. This comment was not fully addressed: Page3. Line 7. Effect of the incomplete annotation is known and there is extensive literature on this. For example Mangul, Serghei, et al. "Improved transcriptome quantification and reconstruction from RNA-Seq reads using partial annotations." In silico biology 11.5. The proposed or similar paper need to be cited,. The authors need to discuss the effect of completeness of annotations on the quantification accuracy. Long read technologies are able to discover novel transcripts that are not part of the reference and thus suggesting incompleteness of the current annotation databases.

Reply: Thanks for the suggestion. We have added more discussion:

"We further addressed the problem of using partial transcriptome annotation (Figure 1, Additional file 3). Full transcriptome annotation is always recommended for RNA-Seq analysis when it is available to improve accuracy. When studying organisms with poorly-annotated transcriptome, it is advisable to assemble and reconstruct the transcriptome first. Furthermore, with the advent of long read sequencing technologies, more novel transcripts are expected to be identified even for well-annotated transcriptome such as human's. Several methods have been developed for transcriptome assembly and reconstruction, including Cufflinks {\cite{trapnell2012}}, Trinity {\cite{grabherr2011}}, TransPS {\cite{liu2014}}, and DRUT {\cite{mangul2011}}. }"

6. This comment was not addressed. "The main limitation of the study is that only simulated reads were used. Authors need to mention the limitation of the simulated data as such data usually the reality. This was recently highlighted in a recent paper about benchmarking: Mangul, Serghei, et al. "Systematic benchmarking of omics computational tools." Nature communications 10 (2019). " Authors need to

mention what are the limitations of the simulated data and cite the corresponding literature.

Reply: We have further addressed the limitation of the study in the discussion:

“Another limitation is that only simulated data is used in the study. Simulated data may not capture the complexity of real data and true experimental variability. A more comprehensive approach to complement the simulated data with experimental data should be considered in further benchmark studies {\cite{mangul2019}}. More vigorous evaluation using real expression data from other platforms and experimental validations such as reverse transcriptase PCR can be carried out in future.”

Close
