# Supplementary material for: Benchmark of long non-coding RNA quantification for RNA sequencing of cancer samples
Source: Gigascience. 2019 Dec 6;8(12):giz145. doi: 10.1093/gigascience/giz145 (PMC6897288; doi:10.1093/gigascience/giz145)
Supplement: giz145_Response_to_Reviewer_Comments_Revision_2 [file giz145_response_to_reviewer_comments_revision_2.pdf]

Author's Response To Reviewer Comments

Close

Dear Editor and Reviewers,

Thanks for accepting our study for publication in GigaScience. We have finished preparing the GigaDB entry for the study and also cited it in the data availability section in the updated manuscript. We look forward to getting the manuscript published!

Close
