# Supplementary material for: Benchmark of long non-coding RNA quantification for RNA sequencing of cancer samples
Source: Gigascience. 2019 Dec 6;8(12):giz145. doi: 10.1093/gigascience/giz145 (PMC6897288; doi:10.1093/gigascience/giz145)
Supplement: giz145_Response_to_Reviewer_Comments_Revision_3 [file giz145_response_to_reviewer_comments_revision_3.pdf]

Author's Response To Reviewer Comments

Close

Dear Editor,

We have made the changes:

1) Corrected the GigaDB link in citation (http://dx.doi.org/10.5524/100671)
2) Added "licence: MIT" in the code availability section
3) Included the "Stanford medicine box" and web interface urls in the bibliography and cited them by number.

Any further changes needed, please let us know!
Best regard,
Olivier

Close
