# Supplementary material for: Benchmark of long non-coding RNA quantification for RNA sequencing of cancer samples
Source: Gigascience. 2019 Dec 6;8(12):giz145. doi: 10.1093/gigascience/giz145 (PMC6897288; doi:10.1093/gigascience/giz145)
Supplement: giz145_Reviewer_1_Report_Original_Submission — Bo Li, Ph.D. -- 6/2/2019 Reviewed [file giz145_reviewer_1_report_original_submission.pdf]

Reviewer Report

Title: Benchmark of lncRNA Quantification for RNA-Seq of Cancer Samples

Version: Original Submission **Date:** 6/2/2019

Reviewer name: Bo Li, Ph.D.

Reviewer Comments to Author:

This manuscript described a careful benchmarking design to evalucate the lncRNA quantification performance of several pipelines for RNA-Seq data of cancer samples. The authors showed that methods that only utilize unique reads performed badly for lincRNA quantification. This result is important and timely since HTSeq is the current default workflow for TCGA data and it is crucial for the cancer research community to understand its strenghs and weakness.
I strongly recommend the authors to address the 3 major comments below, which can significantly improve their manuscript:
1) The conclusion that "pseudoalignment methods outperform alignment-based methods" is inappropriate.
As the authors mentioned, the plausible reason that Kallisto and Salmon outperforms HTSeq and featureCounts is Kallisto and Salmon models multi-mapping reads better. In fact, RSEM is also an alignment-based method and can allocate multi-mapping reads appropriately. I suggest the authors to change the conclusion to "methods utilizing multi-mapping reads outperforms methods that only use unique reads".
2) Please include RSEM in the benchmarking.
RSEM is also an alignment-based method. Please also benchmark RSEM + STAR, RSEM + HISAT2, and RSEM + Subread.
3) Please consider to conduct some benchmark experiments using real data.
The benchmark conducted in this manuscript is based on simulated data sets using the RSEM simulator. Since both Kallisto and Salmon use graphical models that are highly similar to RSEM's model, there might be a bias in the benchmarking. Thus, it will be greate if we can see some benchmarking results purely based on real datasets.

Methods

Are the methods appropriate to the aims of the study, are they well described, and are necessary controls included? Choose an item.

Conclusions

Are the conclusions adequately supported by the data shown? Choose an item.

Reporting Standards

Does the manuscript adhere to the journal's guidelines on [minimum standards of reporting?] Choose an item.

Choose an item.

Statistics

Are you able to assess all statistics in the manuscript, including the appropriateness of statistical tests used? Choose an item.

Quality of Written English

Please indicate the quality of language in the manuscript: Choose an item.

Declaration of Competing Interests

Please complete a declaration of competing interests, considering the following questions:

- Have you in the past five years received reimbursements, fees, funding, or salary from an organisation that may in any way gain or lose financially from the publication of this manuscript, either now or in the future?
- Do you hold any stocks or shares in an organisation that may in any way gain or lose financially from the publication of this manuscript, either now or in the future?
- Do you hold or are you currently applying for any patents relating to the content of the manuscript?
- Have you received reimbursements, fees, funding, or salary from an organization that holds or has applied for patents relating to the content of the manuscript?
- Do you have any other financial competing interests?
- Do you have any non-financial competing interests in relation to this paper?

If you can answer no to all of the above, write 'I declare that I have no competing interests' below. If your reply is yes to any, please give details below.

I declare that I have no competing interests.

I agree to the open peer review policy of the journal. I understand that my name will be included on my report to the authors and, if the manuscript is accepted for publication, my named report including any attachments I upload will be posted on the website along with the authors' responses. I agree for my report to be made available under an Open Access Creative Commons CC-BY license (http://creativecommons.org/licenses/by/4.0/). I understand that any comments which I do not wish to be included in my named report can be included as confidential comments to the editors, which will not be published.

Choose an item.

To further support our reviewers, we have joined with Publons, where you can gain additional credit to further highlight your hard work (see: https://publons.com/journal/530/gigascience). On publication of this paper, your review will be automatically added to Publons, you can then choose whether or not to claim your Publons credit. I understand this statement.

Yes Choose an item.
