# Supplementary material for: Benchmark of long non-coding RNA quantification for RNA sequencing of cancer samples
Source: Gigascience. 2019 Dec 6;8(12):giz145. doi: 10.1093/gigascience/giz145 (PMC6897288; doi:10.1093/gigascience/giz145)
Supplement: giz145_Reviewer_2_Report_Original_Submission — Serghei Mangul -- 6/5/2019 Reviewed [file giz145_reviewer_2_report_original_submission.pdf]

Reviewer Report

Title: Benchmark of lncRNA Quantification for RNA-Seq of Cancer Samples

Version: Original Submission **Date:** 6/5/2019

Reviewer name: Serghei Mangul

Reviewer Comments to Author:

Major comments
The methods chosen for benchmarking do not include the EM-based methods to estimate gene expressions: e..g RSEM or IsoEM. RSEM was only used to generate gold standard gene expressions. This might explain low accuracy of mapping based gene quantification methods.
The manuscripts also lack details of which mapping methods provided the best results. Was is the same method for both htseq and featureCount
It would be beneficial to explore the effect of parameters (of the mapping tools) on the accuracy of quantification. It was suggested here that the tuned parameters usually provide better results: Baruzzo, Giacomo, et al. "Simulation-based comprehensive benchmarking of RNA-seq aligners." Nature methods 14.2 (2017): 135.
On a similar note, it would be interesting to explore what is causing low performance of mapping -based tool. Is it an incorrect alignment? To quantify this, authors can use .bam files with true alignment where reads are "mapped" to the position from where they were simulated. It will be interesting to compare this across un stranded and reverse standard libraries.
The study does not explore the important sequencing features including read length and throughput (number of reads). It would be beneficial to add this at least for several datasets and report how the accuracy of gene quantification depends in these parameters.
Authors use RSEM to generate which will be used for simulating the reads. Why RSEM was chosen? Also, do the reads contain sequencing errors and SNPs? Were the reads paired-end reads? If so what was the fragment length distribution?
The main limitation of the study is that only simulated reads were used. Authors need to mention the limitation of the simulated data as such data usually the reality. This was recently highlighted in a recent paper about benchmarking: Mangul, Serghei, et al. "Systematic benchmarking of omics computational tools." Nature communications 10 (2019).
It is not clear what authors mean by sample-level and gene-level. More details needs to be provided.
Discussion section lack discussion on which alignment was the best. And in general what was causing low performance of mapping based methods. If it will be determined to be mapping quality (see point point 4) when such discussion needs to be added. Or maybe it is due to the fact that both mapping-based methods were non-EM. and RSEM might show much better results.
The formal comparison of max RAM and CPU time for each method needs to be added into a separate figure. The running time for mapping based methods needs to be split into method itself and mapping.
In addition to used measured it would be beneficial to use: error fraction (EF) and median percent error (MPE) measures as was suggested here Nicolae, Marius, et al. "Estimation of alternative splicing isoform

frequencies from RNA-Seq data." Algorithms for molecular biology 6.1 (2011): 9. Those measures are particularly sensitive for low expressed genes.
Minor comments
Page 2. Line 50. I think the authors mean that the majority of TCGA samples were prepared
Page3. Line 7. Effect of incomplete annotation is know and there is extensive litarature on this. For example Mangul, Serghei, et al. "Improved transcriptome quantification and reconstruction from RNA-Seq reads using partial annotations." In silico biology 11.5, 6 (2011): 251-261.
P 3. Line 57 Is it not clear what the three pipelines are referring to

Methods

Are the methods appropriate to the aims of the study, are they well described, and are necessary controls included? Choose an item.

Conclusions

Are the conclusions adequately supported by the data shown? Choose an item.

Reporting Standards

Does the manuscript adhere to the journal's guidelines on minimum standards of reporting? Choose an item.

Choose an item.

Statistics

Are you able to assess all statistics in the manuscript, including the appropriateness of statistical tests used? Choose an item.

Quality of Written English

Please indicate the quality of language in the manuscript: Choose an item.

Declaration of Competing Interests

Please complete a declaration of competing interests, considering the following questions:

- Have you in the past five years received reimbursements, fees, funding, or salary from an organisation that may in any way gain or lose financially from the publication of this manuscript, either now or in the future?
- Do you hold any stocks or shares in an organisation that may in any way gain or lose financially from the publication of this manuscript, either now or in the future?

- Do you hold or are you currently applying for any patents relating to the content of the manuscript?
- Have you received reimbursements, fees, funding, or salary from an organization that holds or has applied for patents relating to the content of the manuscript?
- Do you have any other financial competing interests?
- Do you have any non-financial competing interests in relation to this paper?

If you can answer no to all of the above, write 'I declare that I have no competing interests' below. If your reply is yes to any, please give details below.

I declare that I have no competing interests

I agree to the open peer review policy of the journal. I understand that my name will be included on my report to the authors and, if the manuscript is accepted for publication, my named report including any attachments I upload will be posted on the website along with the authors' responses. I agree for my report to be made available under an Open Access Creative Commons CC-BY license (http://creativecommons.org/licenses/by/4.0/). I understand that any comments which I do not wish to be included in my named report can be included as confidential comments to the editors, which will not be published.

Choose an item.

To further support our reviewers, we have joined with Publons, where you can gain additional credit to further highlight your hard work (see: https://publons.com/journal/530/gigascience). On publication of this paper, your review will be automatically added to Publons, you can then choose whether or not to claim your Publons credit. I understand this statement.

Yes Choose an item.
