# Supplementary material for: Benchmark of long non-coding RNA quantification for RNA sequencing of cancer samples
Source: Gigascience. 2019 Dec 6;8(12):giz145. doi: 10.1093/gigascience/giz145 (PMC6897288; doi:10.1093/gigascience/giz145)
Supplement: giz145_Reviewer_2_Report_Revision_1 — Serghei Mangul -- 10/23/2019 Reviewed [file giz145_reviewer_2_report_revision_1.pdf]

Reviewer Report

Title: Benchmark of lncRNA Quantification for RNA-Seq of Cancer Samples

Version: Revision 1 **Date:** 10/23/2019

Reviewer name: Serghei Mangul

Reviewer Comments to Author:

1. The answers prepared by the authors were not properly formatted. It lacks numbering and having the added text or figure directly in the answer will be extremely useful
2. RSEM is in the wrong category. Since it requires the mapping it should be in a similar category as other Quantification tools
3. It will be useful to plot CPU time vs accuracy to emphasise the ability of pseudo alignment methods to deliver similar results in a short amount of time
4. Similar methods as RSEM need to be cited. For example IsoEM
5. This comment was not fully addressed: Page3. Line 7. Effect of the incomplete annotation is known and there is extensive literature on this. For example Mangul, Serghei, et al. "Improved transcriptome quantification and reconstruction from RNA-Seq reads using partial annotations." In silico biology 11.5. The proposed or similar paper need to be cited,. The authors need to discuss the effect of completeness of annotations on the quantification accuracy. Long read technologies are able to discover novel transcripts that are not part of the reference and thus suggesting incompleteness of the current annotation databases.
6. This comment was not addressed. "The main limitation of the study is that only simulated reads were used. Authors need to mention the limitation of the simulated data as such data usually the reality. This was recently highlighted in a recent paper about benchmarking: Mangul, Serghei, et al. "Systematic benchmarking of omics computational tools." Nature communications 10 (2019). " Authors need to mention what are the limitations of the simulated data and cite the corresponding literature.

Methods

Are the methods appropriate to the aims of the study, are they well described, and are necessary controls included? Choose an item.

Conclusions

Are the conclusions adequately supported by the data shown? Choose an item.

Reporting Standards

Does the manuscript adhere to the journal's guidelines on [minimum standards of reporting?]() Choose an item.

Choose an item.

Statistics

Are you able to assess all statistics in the manuscript, including the appropriateness of statistical tests used? Choose an item.

Quality of Written English

Please indicate the quality of language in the manuscript: Choose an item.

Declaration of Competing Interests

Please complete a declaration of competing interests, considering the following questions:

- Have you in the past five years received reimbursements, fees, funding, or salary from an organisation that may in any way gain or lose financially from the publication of this manuscript, either now or in the future?
- Do you hold any stocks or shares in an organisation that may in any way gain or lose financially from the publication of this manuscript, either now or in the future?
- Do you hold or are you currently applying for any patents relating to the content of the manuscript?
- Have you received reimbursements, fees, funding, or salary from an organization that holds or has applied for patents relating to the content of the manuscript?
- Do you have any other financial competing interests?
- Do you have any non-financial competing interests in relation to this paper?

If you can answer no to all of the above, write 'I declare that I have no competing interests' below. If your reply is yes to any, please give details below.

I declare that I have no competing interests

I agree to the open peer review policy of the journal. I understand that my name will be included on my report to the authors and, if the manuscript is accepted for publication, my named report including any attachments I upload will be posted on the website along with the authors' responses. I agree for my

report to be made available under an Open Access Creative Commons CC-BY license (http://creativecommons.org/licenses/by/4.0/). I understand that any comments which I do not wish to be included in my named report can be included as confidential comments to the editors, which will not be published.

Choose an item.

To further support our reviewers, we have joined with Publons, where you can gain additional credit to further highlight your hard work (see: https://publons.com/journal/530/gigascience). On publication of this paper, your review will be automatically added to Publons, you can then choose whether or not to claim your Publons credit. I understand this statement.

Yes Choose an item.
